# Global prevalence of mental health issues among the general population during the coronavirus disease-2019 pandemic: a systematic review and meta-analysis

**DOI:** 10.1038/s41598-021-89700-8

**Published:** 2021-05-13

**Authors:** Surapon Nochaiwong, Chidchanok Ruengorn, Kednapa Thavorn, Brian Hutton, Ratanaporn Awiphan, Chabaphai Phosuya, Yongyuth Ruanta, Nahathai Wongpakaran, Tinakon Wongpakaran

**Affiliations:** 1 Department of Pharmaceutical Care, Faculty of Pharmacy, Chiang Mai University, Chiang Mai, 50200 Thailand; 2 Pharmacoepidemiology and Statistics Research Center (PESRC), Faculty of Pharmacy, Chiang Mai University, Chiang Mai, 50200 Thailand; 3 Ottawa Hospital Research Institute, Ottawa Hospital, Ottawa, ON K1H 8L6 Canada; 4 Institute of Clinical and Evaluative Sciences, ICES uOttawa, Ottawa, ON K1Y 4E9 Canada; 5 School of Epidemiology and Public Health, Faculty of Medicine, University of Ottawa, Ottawa, ON K1G 5Z3 Canada; 6 Department of Psychiatry, Faculty of Medicine, Chiang Mai University, Chiang Mai, 50200 Thailand

**Keywords:** Post-traumatic stress disorder, Depression, Anxiety

## Abstract

To provide a contemporary global prevalence of mental health issues among the general population amid the coronavirus disease-2019 (COVID-19) pandemic. We searched electronic databases, preprint databases, grey literature, and unpublished studies from January 1, 2020, to June 16, 2020 (updated on July 11, 2020), with no language restrictions. Observational studies using validated measurement tools and reporting data on mental health issues among the general population were screened to identify all relevant studies. We have included information from 32 different countries and 398,771 participants. The pooled prevalence of mental health issues amid the COVID-19 pandemic varied widely across countries and regions and was higher than previous reports before the COVID-19 outbreak began. The global prevalence estimate was 28.0% for depression; 26.9% for anxiety; 24.1% for post-traumatic stress symptoms; 36.5% for stress; 50.0% for psychological distress; and 27.6% for sleep problems. Data are limited for other aspects of mental health issues. Our findings highlight the disparities between countries in terms of the poverty impacts of COVID-19, preparedness of countries to respond, and economic vulnerabilities that impact the prevalence of mental health problems. Research on the social and economic burden is needed to better manage mental health problems during and after epidemics or pandemics. *Systematic review registration*: PROSPERO CRD 42020177120.

## Introduction

After the World Health Organisation (WHO) declared the rapid worldwide spread of coronavirus disease-2019 (COVID-19) to be a pandemic, there has been a dramatic rise in the prevalence of mental health problems both nationally and globally^1–3^. Early international evidence and reviews have reported the psychological effects of the COVID-19 outbreak on patients and healthcare workers, particularly those in direct contact with affected patients^4–8^. Besides patients with COVID-19, negative emotions and psychosocial distress may occur among the general population due to the wider social impact and public health and governmental response, including strict infection control, quarantine, physical distancing, and national lockdowns^2,9,10^.

Amid the COVID-19 pandemic, several mental health and psychosocial problems, for instance, depressive symptoms, anxiety, stress, post-traumatic stress symptoms (PTSS), sleep problems, and other psychological conditions are of increasing concern and likely to be significant^5,10,11^. Public psychological consequences can arise through direct effects of the COVID-19 pandemic that are sequelae related to fear of contagion and perception of danger^2^. However, financial and economic issues also contribute to mental health problems among the general population in terms of indirect effects^12,13^. Indeed, economic shutdowns have disrupted economies worldwide, particularly in countries with larger domestic outbreaks, low health system preparedness, and high economic vulnerability^14–16^.

The COVID-19 pandemic may affect the mental health of the general population differently based on national health and governmental policies implemented and the public resilience and social norms of each country. Unfortunately, little is known about the global prevalence of mental health problems in the general population during the COVID-19 pandemic. Previous systematic reviews have been limited by the number of participants included, and attention has been focussed on particular conditions and countries, with the majority of studies being conducted in mainland China^5,8,11,17,18^. To the best of our knowledge, evidence on mental health problems among the general population worldwide has not been comprehensively documented in the current COVID-19 pandemic. Therefore, a systematic review and meta-analysis at a global level is needed to provide robust and contemporary evidence to inform public health policies and long-term responses to the COVID-19 pandemic.

As such, we have performed a rigorous systematic review and meta-analysis of all available observational studies to shed light on the effects of the global COVID-19 pandemic on mental health problems among the general population. We aimed to: (1) summarise the prevalence of mental health problems nationally and globally, and (2) describe the prevalence of mental health problems by each WHO region, World Bank income group, and the global index and economic indices responses to the COVID-19 pandemic.

## Methods

This systematic review and meta-analysis was performed in accordance with the Preferred Reporting Items for Systematic Reviews and Meta-Analyses guidelines^19^ and reported in line with the Meta-analysis of Observational Studies in Epidemiology statement (Appendix, Table S1)^20^. The pre-specified protocol was registered in the International Prospective Register of Systematic Reviews (PROSPERO: CRD42020177120).

### Search strategy

We searched electronic databases in collaboration with an experienced medical librarian using an iterative process. PubMed, Medline, Embase, PsycINFO, Web of Science, Scopus, CINAHL, and the Cochrane Library were used to identify all relevant abstracts. As the WHO declared the COVID-19 outbreak to be a public health emergency of international concern on January 30, 2020, we limited the search from January 1, 2020, to June 16, 2020, without any language restrictions. The main keywords used in the search strategy included “coronavirus” or “COVID-19” or “SARS-CoV-2”, AND “mental health” or “psychosocial problems” or “depression” or “anxiety” or “stress” or “distress” or “post-traumatic stress symptoms” or “suicide” or “insomnia” or “sleep problems” (search strategy for each database is provided in the Appendix, Table S2). Relevant articles were also identified from the reference lists of the included studies and previous systematic reviews. To updated and provide comprehensive, evidence-based data during the COVID-19 pandemic, grey literature from Google Scholar and the preprint reports from medRxiv, bioRxiv, and PsyArXiv were supplemented to the bibliographic database searches. A targeted manual search of grey literature and unpublished studies was performed through to July 11, 2020.

### Study selection and data screening

We included observational studies (cross-sectional, case–control, or cohort) that (1) reported the occurrence or provided sufficient data to estimate the prevalence of mental health problems among the general population, and (2) used validated measurement tools for mental health assessment. The pre-specified protocol was amended to permit the inclusion of studies the recruited participants aged 12 years or older and college students as many colleges and universities were closed due to national lockdowns. We excluded studies that (1) were case series/case reports, reviews, or studies with small sample sizes (less than 50 participants); (2) included participants who had currently confirmed with the COVID-19 infection; and (3) surveyed individuals under hospital-based settings. If studies had overlapping participants and survey periods, then the study with the most detailed and relevant information was used.

Eligible titles and abstracts of articles identified by the literature search were screened independently by two reviewers (SN and CR). Then, potentially relevant full-text articles were assessed against the selection criteria for the final set of included studies. Potentially eligible articles that were not written in English were translated before the full-text appraisal. Any disagreement was resolved by discussion.

### Outcomes

The primary outcomes were key parameters that reflect the global mental health status during the COVID-19 pandemic, including depression, anxiety, PTSS, stress, psychological distress, and sleep problems (insomnia or poor sleep). To deliver more evidence regarding the psychological consequences, secondary outcomes of interest included psychological symptoms, suicidal ideation, suicide attempts, loneliness, somatic symptoms, wellbeing, alcohol drinking problems, obsessive–compulsive symptoms, panic disorder, phobia anxiety, and adjustment disorder.

### Data extraction and risk of bias assessment

Two reviewers (SN and YR) independently extracted the pre-specified data using a standardised approach to gather information on the study characteristics (the first author’s name, study design [cross-sectional survey, longitudinal survey, case–control, or cohort], study country, article type [published article, short report/letters/correspondence, or preprint reporting data], the data collection period), participant characteristics (mean or median age of the study population, the proportion of females, proportion of unemployment, history of mental illness, financial problems, and quarantine status [never, past, or current]), and predefined outcomes of interest (including assessment outcome definitions, measurement tool, and diagnostic cut-off criteria). For international studies, data were extracted based on the estimates within each country. For studies that had incomplete data or unclear information, the corresponding author was contacted by email for further clarification. The final set of data was cross-checked by the two reviewers (RA and CP), and discrepancies were addressed through a discussion.

Two reviewers (SN and CR) independently assessed and appraised the methodological quality of the included studies using the Hoy and colleagues Risk of Bias Tool-10 items^21^. A score of 1 (no) or 0 (yes) was assigned to each item. The higher the score, the greater the overall risk of bias of the study, with scores ranging from 0 to 10. The included studies were then categorised as having a low (0–3 points), moderate (4–6 points), or high (7 or 10 points) risk of bias. A pair of reviewers (RA and CP) assessed the risk of bias of each study. Any disagreements were resolved by discussion.

### Data synthesis and statistical methods

A two-tailed *P* value of less than 0.05 was considered statistically significant. We used Stata software version 16.0 (StataCorp, College Station, TX, USA) for all analyses and generated forest plots of the summary pooled prevalence. Inter-rater agreements between reviewers for the study selection and risk of bias assessment were tested using the kappa (κ) coefficient of agreement^22^. Based on the crude information data, we recalculated and estimated the unadjusted prevalence of mental health and psychological problems using the crude numerators and denominators reported by each of the included studies. Unadjusted pooled prevalence with corresponding 95% confidence intervals (CIs) was reported for each WHO regions (Africa, America, South-East Asia, Europe, Eastern Mediterranean, and Western Pacific) and World Bank income group (low-, lower-middle-, upper-middle-, and high-income).

We employed the variance of the study-specific prevalence using the Freeman–Tukey double arcsine methods for transforming the crude data before pooling the effect estimates with a random-effect model to account for the effects of studies with extreme (small or large) prevalence estimates^23^. Heterogeneity was evaluated using the Cochran’s *Q* test, with a *p* value of less than 0.10^24^. The degree of inconsistency was quantified using *I*^2^ values, in which a value greater than 60–70% indicated the presence of substantial heterogeneity^25^.

Pre-planned subgroup analyses were performed based on the participant (i.e., age, the proportion of female sex, the proportion of unemployment, history of mental illness, financial problems, and quarantine status) and study characteristics (article type, study design, data collection, and sample size). To explore the inequality and poverty impacts across countries, subgroup analyses based on the global index and economic indices responses to the COVID-19 pandemic were performed, including (1) human development index (HDI) 2018 (low, medium, high, and very high)^26^; (2) gender inequality index 2018 (below vs above world average [0.439])^27^; (3) the COVID-19-government response stringency index during the survey (less- [less than 75%], moderate- [75–85%], and very stringent [more than 85%]) according to the Oxford COVID-19 Government Response Tracker reports^28^; (4) the preparedness of countries in terms of hospital beds per 10,000 people, 2010–2018 (low, medium–low, medium, medium–high, and high)^15^; (5) the preparedness of countries in terms of current health expenditure (% of gross domestic product [GDP] 2016; low, medium–low, medium, medium–high, and high)^15^; (6) estimated percent change of real GDP growth based on the International Monetary Fund, April 2020 (below vs above world average [− 3.0])^29^; (7) the resilience of countries’ business environment based on the 2020 global resilience index reports (first-, second-, third-, and fourth-quartile)^30^; and (8) immediate economic vulnerability in terms of inbound tourism expenditure (% of GDP 2016–2018; low, medium–low, medium, medium–high, and high)^15^.

To address the robustness of our findings, we conducted a sensitivity analysis by restricting the analysis to studies with a low risk of bias (Hoy and Colleagues-Tool, 0–3 points). Furthermore, a random-effects univariate meta-regression analysis was used to explore the effect of participant and study characteristics, and the global index and economic indices responses to the COVID-19 pandemic as described above on the prevalence estimates.

The visual inspection of funnel plots was performed when there was sufficient data and tested for asymmetry using the Begg’s and Egger’s tests for each specific. A *P* value of less than 0.10 was considered to indicate statistical publication bias^31,32^. If the publication bias was detected by the Begg’s and Egger’s regression test, the trim and fill method was then performed to calibrate for publication bias^33^.

## Results

Initially, the search strategy retrieved 4642 records. From these, 2682 duplicate records were removed, and 1960 records remained. Based on the title and abstract screening, we identified 498 articles that seemed to be relevant to the study question (the κ statistic for agreement between reviewers was 0.81). Of these, 107 studies fulfilled the study selection criteria and were included in the meta-analysis (Appendix, Figure S1). The inter-rater agreement between reviewers on the study selection and data extraction was 0.86 and 0.75, respectively. The reference list of all included studies in this review is provided in the Appendix, Table S3.

### Characteristics of included studies

In total, 398,771 participants from 32 different countries were included. The mean age was 33.5 ± 9.5 years, and the proportion of female sex was 60.9% (range, 16.0–51.6%). Table 1 summarises the characteristics of all the included studies according to World Bank income group, the global index of COVID-19 pandemic preparedness, and economic vulnerability indices. The included studies were conducted in the Africa (2 studies^34,35^ [1.9%], n = 723), America (12 studies^36–47^ [11.2%], n = 18,440), South-East Asia (10 studies^48–57^ [9.4%], n = 11,953), Europe (27 studies^58–84^ [25.2%], n = 148,430), Eastern Mediterranean (12 studies^85–96^ [11.2%], n = 23,396), and Western Pacific WHO regions (44 studies^97–140^ [41.1%], n = 195,829). Most of the included studies were cross-sectional (96 studies, 89.7%), used an online-based survey (101 studies, 95.3%), conducted in mainland China (34 studies, 31.8%), and were conducted in countries with upper-middle (49 studies, 45.8%) and high-incomes (44 studies, 41.1%). Detailed characteristics of the 107 included studies, measurement tools for evaluating the mental health status and psychological consequences, and the diagnostic cut-off criteria are described in Appendix, Table S4. Of the 107 included studies, 76 (71.0%) had a low risk, 31 (29.0%) had a moderate risk, and no studies had a high risk of bias (Appendix, Table S5).

**Table 1:** Region, global index of COVID-19 pandemic and economic, and characteristics of the 107 included studies.

Characteristics of included studies	Number of studies (%)
**WHO Region: Country (no. of studies)**	
Africa Region: Nigeria (1), South Africa (1)	2 (1.9)
Region of the Americas: Brazil (4), Mexico (1), United States (7)	12 (11.2)
South-East Asia Region: Bangladesh (3), India (5), Nepal (1), Thailand (1)	10 (9.4)
European Region: Germany (1), Greece (1), Ireland (1), Italy (6), Norway (1), Portugal (1), Spain (6), Sweden (1), Turkey (2), United Kingdom (7)	27 (25.2)
Eastern Mediterranean Region: Egypt (1), Iran (3), Jordan (2), Pakistan (1), Saudi Arabia (2), Tunisia (1), United Arab Emirates (2)	12 (11.2)
Western Pacific Region: Australia (4), China (including Hong Kong, Macau, Taiwan; 36), Japan (1), Malaysia (1), New Zealand (1), Vietnam (1)	44 (41.1)
**World Bank, by income groups**	
Low income	…
Lower-middle income	14 (13.1)
Upper-middle income	49 (45.8)
High income	44 (41.1)
**Human development groups, 2018**^**a**^	
Low	1 (0.9)
Medium	11 (10.3)
High	48 (44.9)
Very high	47 (43.9)
**Gender Inequality Index, 2018 (world average, 0.439)**^**a**^	
Below world average gender inequality index	88 (82.2)
Above world average gender inequality index	16 (15.0)
No data	3 (2.8)
**COVID-19: Government Response Stringency Index during the survey**^**b**^	
Less stringent (< 75)	23 (21.5)
Moderate stringent (75–85)	53 (49.5)
Very stringent (> 85)	29 (27.1)
No data	2 (1.9)
**Preparedness of countries to respond to COVID-19: hospital beds per 10,000 people, 2010–2018**^**c**^	
Low	10 (9.4)
Medium–low	9 (8.4)
Medium	22 (20.6)
Medium–high	60 (56.1)
High	2 (1.9)
No data	4 (3.7)
**Preparedness of countries to respond to COVID-19: current health expenditure (% of GDP), 2016**^**c**^	
Low	14 (13.1)
Medium–low	40 (37.4)
Medium	5 (4.7)
Medium–high	18 (16.8)
High	28 (26.2)
Not data	2 (1.9)
**Real GDP growth 2020, estimates percent change (world average, -3.0)**^**d**^	
Below GDP world average growth	58 (54.2)
Above GDP world average growth	49 (45.8)
**Resilience of a country's business environment**^**e**^	
First quartile	41 (38.3)
Second quartile	17 (15.9)
Third quartile	40 (37.4)
Fourth quartile	9 (8.4)
**Immediate economic vulnerability: inbound tourism expenditure (% of GDP 2016–2018)**^**c**^	
Low	52 (48.6)
Medium–low	19 (17.8)
Medium	15 (14.0)
Medium–high	14 (13.1)
High	7 (6.5)
**Article type**	
Published article	48 (44.9)
Preprint reporting data	47 (43.9)
Short communication/letter to editor/correspondence	12 (11.2)
**Study design**	
Cross-sectional study	96 (89.7)
Longitudinal survey	12 (10.3)
**Data collection**	
Online survey	101 (95.3)
Online and telephone survey	1 (0.9)
Telephone survey	1 (0.9)
Paper-based survey	1 (0.9)
No data	2 (1.9)
**Risk of bias**	
Low	76 (71.0)
Moderate	31 (29.0)
High	…
Mean age in year, grand mean ± S.D.; range (min–max); missing data for 39 studies (36.4%)	33.5 ± 9.5 (16.0 – 51.6)
% Female, mean; range (min–max); missing data for 3 studies (2.8%)	60.9 (24.8 – 85.8)
Sample size, median (min–max)	1255 (66 – 56,679)
**Outcomes reported**	
Depression	75 (70.1)
Anxiety	75 (70.1)
Post-traumatic stress symptoms	28 (26.2)
Stress	22 (20.6)
Psychological distress	18 (16.8)
Sleep problems (insomnia/poor sleep)	15 (14.0)
Psychological symptoms	4 (3.7)
Suicide ideation	4 (3.7)
Loneliness	3 (2.8)
Somatic symptoms	3 (2.8)
Wellbeing	3 (2.8)
Alcohol drinking problems	2 (1.9)
Obsessive–compulsive symptoms	2 (1.9)
Panic disorder	1 (0.9)
Phobia anxiety	1 (0.9)
Adjustment disorder	1 (0.9)
Suicide attempts	1 (0.9)

*COVID-19* coronavirus disease-2019, *GDP* gross domestic product, *WHO* World Health Organisation.

^a^Based on the 2019 Human Development Report by the United Nations Development Programme.

^b^Based on the Oxford COVID-19 Government Response Tracker—highest value during the surveys.

^c^Based on the 2020 global preparedness and vulnerability to respond to COVID-19 pandemic by the United Nations Development Programme.

^d^Based on the World Economic Outlook, April 2020 by the International Monetary Fund.

^e^Based on the 2020 FM Global Resilience Index.

### Global prevalence of mental health issues among the general population amid the COVID-19 pandemic

**Figure 1:**
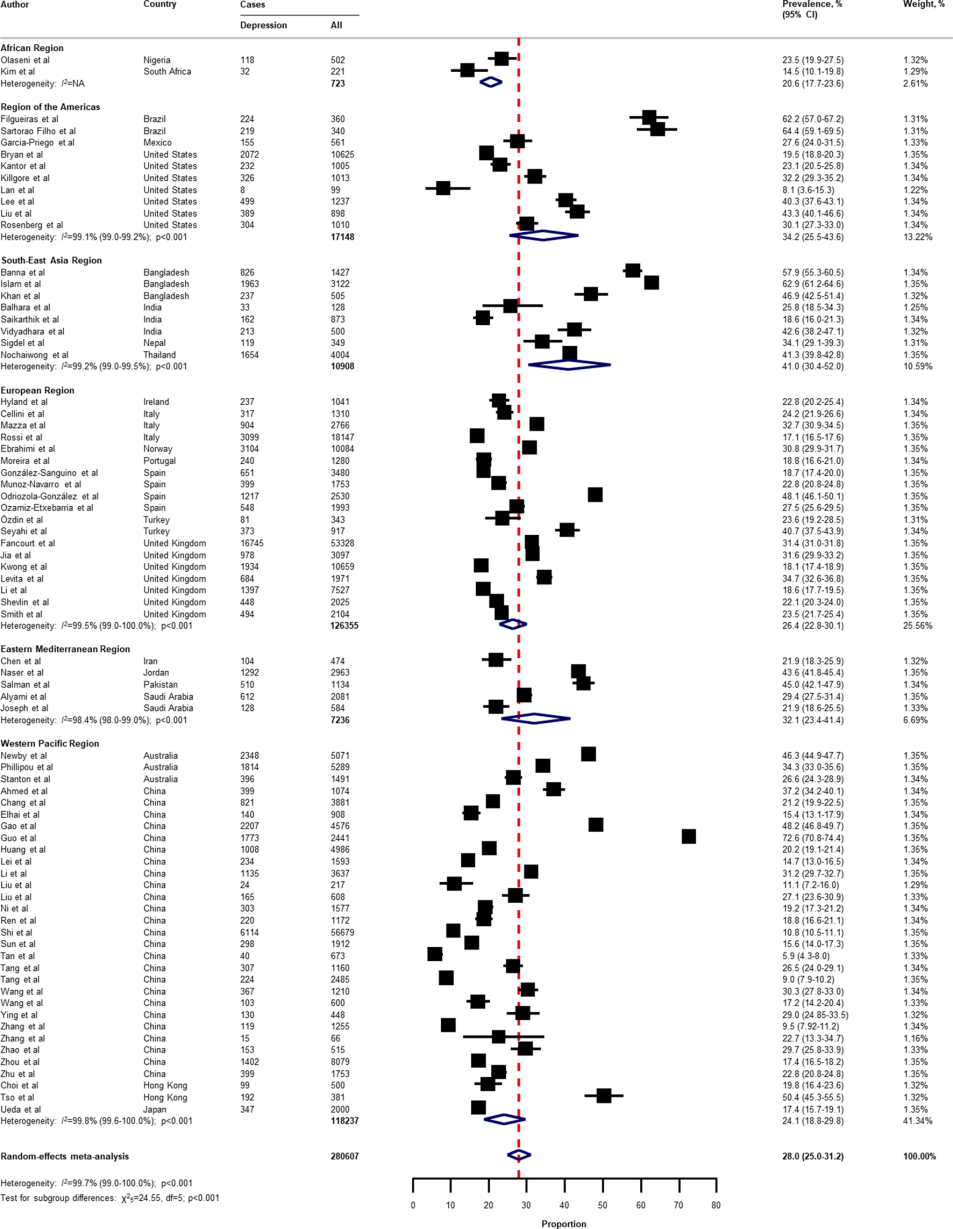
Pooled prevalence of depression among the general population amid the COVID-19 pandemic. *COVID-19* coronavirus disease 2019, *CI* confidence interval, *df* degree of freedom, *NA* not applicable. References are listed according to WHO region in the appendix, Table S3.

**Figure 2:**
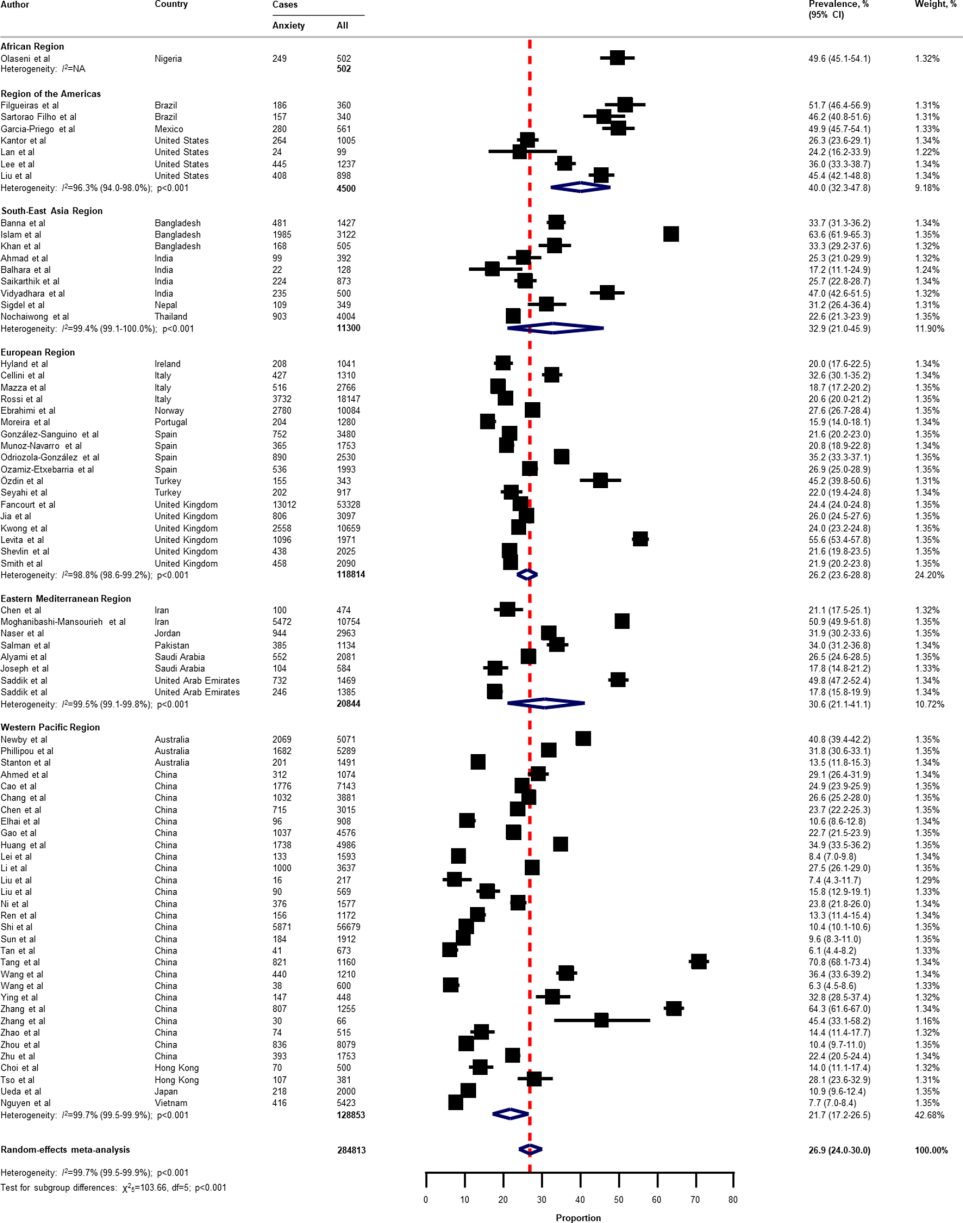
Pooled prevalence of anxiety among the general population amid the COVID-19 pandemic. *COVID-19* coronavirus disease 2019, *CI* confidence interval, *df* degree of freedom, *NA* not applicable. References are listed according to WHO region in the appendix, Table S3.

**Figure 3:**
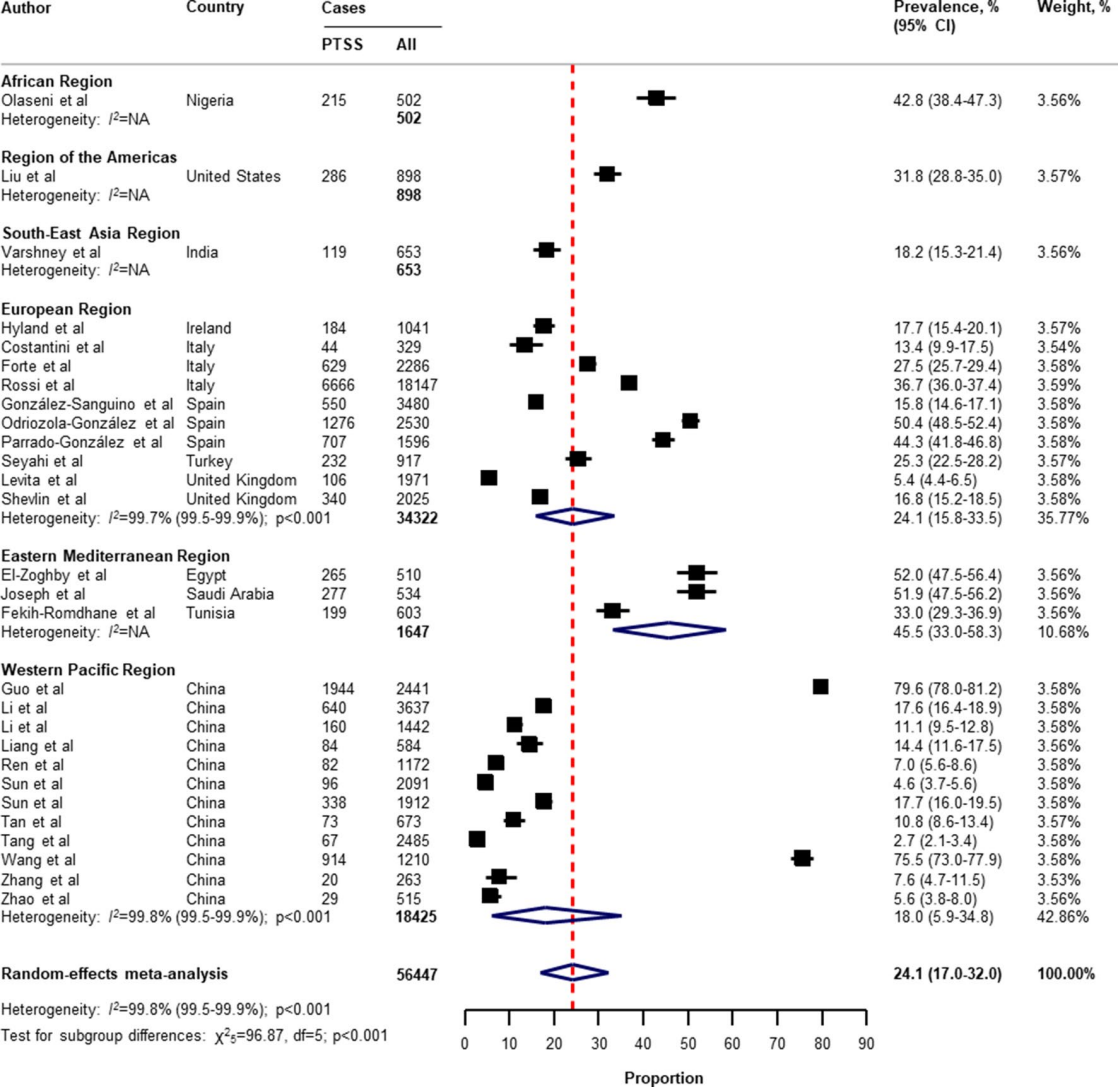
Pooled prevalence of PTSS among the general population amid the COVID-19 pandemic. *COVID-19* coronavirus disease 2019, *CI* confidence interval, *df* degree of freedom, *NA* not applicable, *PTSS* post-traumatic stress symptoms. References are listed according to WHO region in the appendix, Table S3.

**Figure 4:**
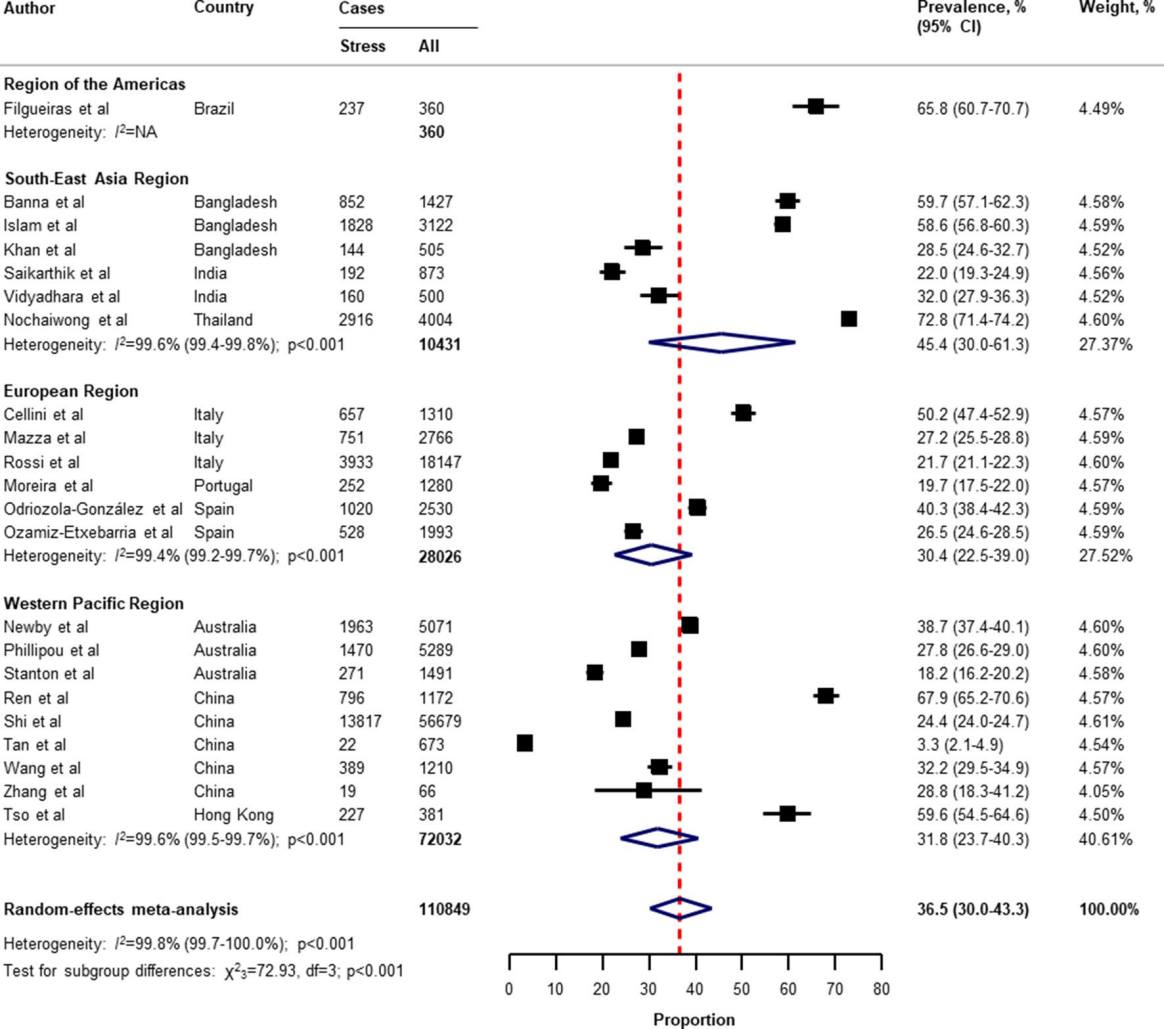
Pooled prevalence of stress among the general population amid the COVID-19 pandemic. *COVID-19* coronavirus disease 2019, *CI* confidence interval, *df* degree of freedom, *NA* not applicable. References are listed according to WHO region in the appendix, Table S3.

**Figure 5:**
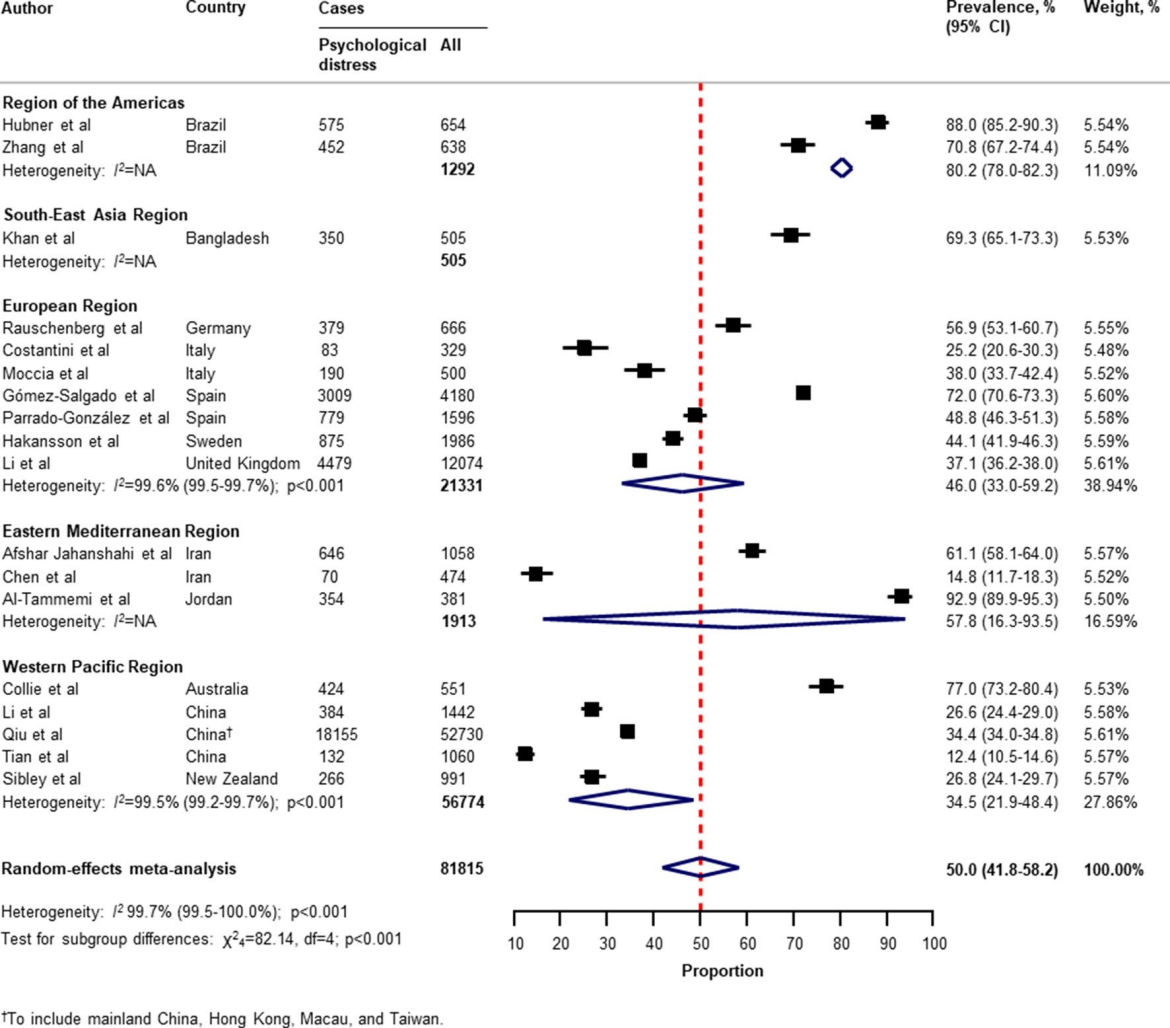
Pooled prevalence of psychological distress among the general population amid the COVID-19 pandemic. *COVID-19* coronavirus disease 2019, *CI* confidence interval, *df* degree of freedom, *NA* not applicable. References are listed according to WHO region in the appendix, Table S3.

**Figure 6:**
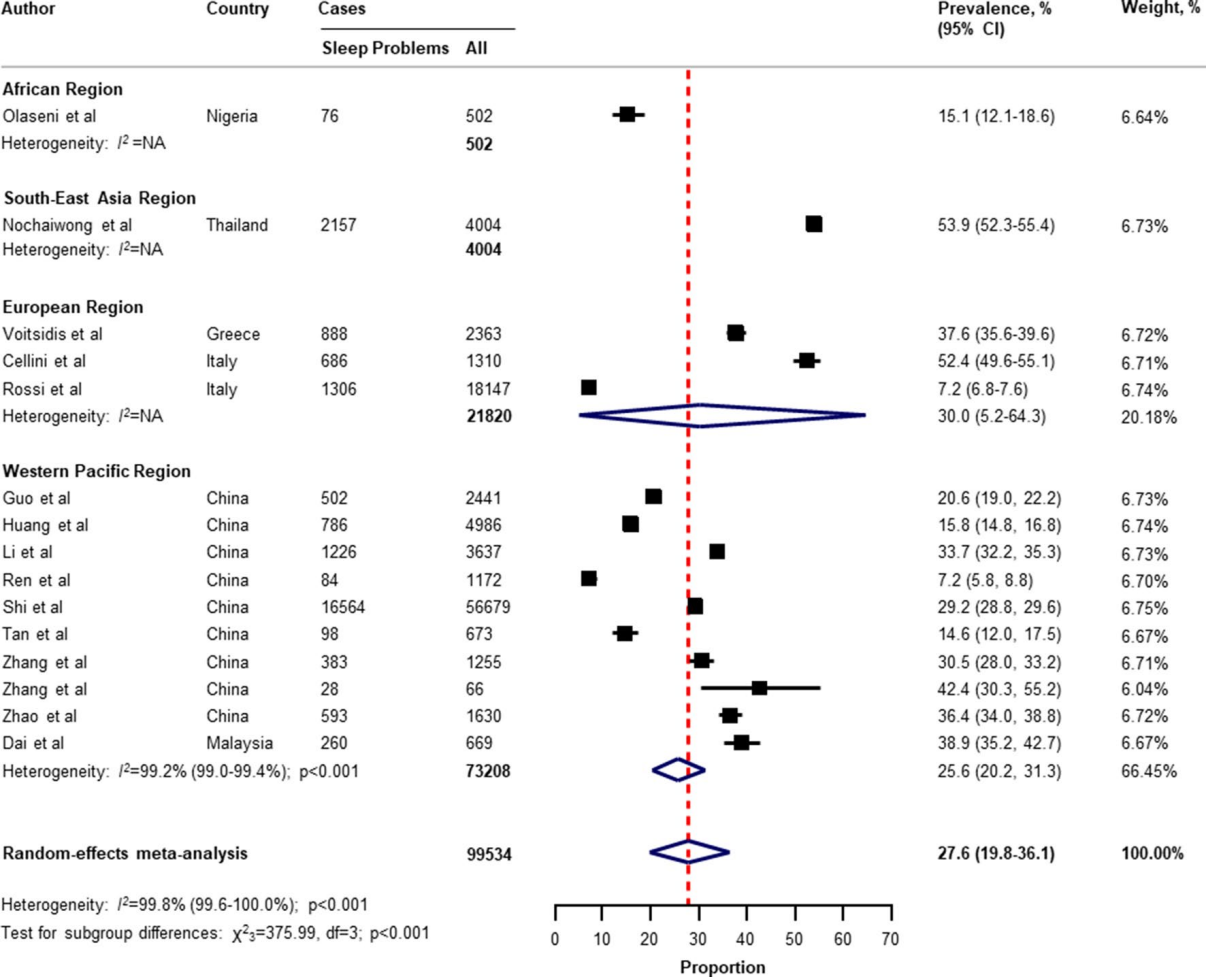
Pooled prevalence of sleep problems among the general population amid the COVID-19 pandemic. *COVID-19* coronavirus disease 2019, *CI* confidence interval, *df* degree of freedom, *NA* not applicable. References are listed according to WHO region in the appendix, Table S3.

Table 2 presents a summary of the results of the prevalence of mental health problems among the general population amid the COVID-19 pandemic by WHO region and World Bank country groups. With substantial heterogeneity, the global prevalence was 28.0% (95% CI 25.0–31.2) for depression (75 studies^34–38,40–46,49–55,57,58,60,61,64,66–71,73–77,80–83,87,88,91,93,96,97,99,101,104–109,112–114,116,117,119,120,122,124–127,129–134,136,138–140^, n = 280,607, Fig. 1); 26.9% (95% CI 24.0–30.0) for anxiety (75 studies^35,37,38,40,42–44,46,48–55,57,58,60,61,64,66–69,71,73–77,80–83,87,88,91–101,104,105,107–109,112–117,119,120,122,124–126,129–134,136,138–140^, n = 284,813, Fig. 2); 24.1% (95% CI 17.0–32.0) for PTSS (28 studies^35,44,56,59,62,64,66,69,75,78,80–82,89–91,106,109–111,119,123–125,127,131,135,138^, n = 56,447, Fig. 3); 36.5% (95% CI 30.0–43.3) for stress (22 studies^37,50–54,57,58,71,73,75,76,80,114,117,119,120,122,125,129,131,136^, n = 110,849, Fig. 4); 50.0% (95% CI 41.8–58.2) for psychological distress (18 studies^39,47,52,59,63,65,70,72,78,79,85,86,88,102,110,118,121,128^, n = 81,815, Fig. 5); and 27.6% (95% CI 19.8–36.1) for sleep problems (15 studies^35,53,58,80,84,103,106,107,109,119,120,125,134,136,137^, n = 99,534, Fig. 6). The prevalence of mental health problems based on different countries varied (Appendix, Table S6), from 14.5% (South Africa) to 63.3% (Brazil) for depressive symptoms; from 7.7% (Vietnam) to 49.9% (Mexico) for anxiety; from 10.5% (United Kingdom) to 52.0% (Egypt) for PTSS; from 19.7% (Portugal) to 72.8% (Thailand) for stress; from 23.9% (China) to Jordan (92.9%) for psychological distress; from 9.2% (Italy) to 53.9% (Thailand) for sleep problems.

**Table 2:** Summary of mental health problems prevalence estimates among the general population, by WHO region and World Bank country groups.

Outcomes	Overall	WHO region						World Bank groups		
		Africa region	Region of the Americas	South-East Asia Region	European Region	Eastern Mediterranean Region	Western Pacific Region	Lower-middle income	Upper- middle income	High income
**Depression**										
No. of studies (participant)	75 (280,607)	2 (723)	10 (17,148)	8 (10,908)	19 (126,355)	5 (7236)	31 (118,237)	9 (8540)	34 (113,688)	32 (158,379)
Prevalence (95% CI)	28.0% (25.0–31.2)	20.6% (17.7–23.6)	34.2% (25.5–43.6)	41.0% (30.4–52.0)	26.4% (22.8–30.1)	32.1% (23.4–41.4)	24.1% (18.8–29.8)	39.3% (28.1–51.1)	26.0% (20.6–31.8)	27.3% (24.3–30.4)
*I*^2^ (95% CI)	99.7% (99.0–100)	…	99.1% (99.0–99.2)	99.2% (99.0–99.5)	99.5% (99.0–100)	98.4% (98.0–99.0)	99.8% (99.6–100)	99.1% (99.0–99.2)	99.7% (99.5–100)	99.4% (99.0–99.7)
*P* value for heterogeneity	< 0.001	…	< 0.001	< 0.001	< 0.001	< 0.001	< 0.001	< 0.001	< 0.001	< 0.001
*P* value for difference	…	< 0.001						0.100		
**Anxiety**										
No. of studies (participant)	75 (284,813)	1 (502)	7 (4500)	9 (11,300)	18 (118,814)	8 (20,844)	32 (128,853)	11 (14,355)	34 (129,414)	30 (141,044)
Prevalence (95% CI)	26.9% (24.0–30.0)	49.6% (45.1–54.1)	40.0% (32.3–47.8)	32.9% (21.0–45.9)	26.2% (23.6–28.8)	30.6% (21.1–41.1)	21.7% (17.2–26.5)	32.6% (18.2–48.9)	26.0% (20.6–31.8)	26.0% (23.4–28.7)
*I*^2^ (95% CI)	99.7% (99.5–99.9)	…	96.3% (94.0–98.0)	99.4% (99.1–100)	98.8% (98.6–99.2)	99.5% (99.1–99.8)	99.7% (99.5–99.9)	99.7% (99.6–99.8)	99.8% (99.6–99.9)	99.1% (99.0–99.2)
*P* value for heterogeneity	< 0.001	…	< 0.001	< 0.001	< 0.001	< 0.001	< 0.001	< 0.001	< 0.001	< 0.001
*P* value for difference	…	< 0.001						0.700		
**Post-traumatic stress symptoms**										
No. of studies (participant)	28 (56,447)	1 (502)	1 (898)	1 (653)	10 (34,322)	3 (1647)	12 (18,425)	4 (2268)	13 (19,342)	11 (34,837)
Prevalence (95% CI)	24.1% (17.0–32.0)	42.8% (38.4–47.3)	31.8% (28.8–35.0)	18.2% (15.3–21.4)	24.1% (15.8–33.5)	45.5% (33.0–58.3)	18.0% (5.9–34.8)	35.9% (21.9–51.3)	18.5% (6.8–34.3)	26.9% (18.6–36.2)
*I*^2^ (95% CI)	99.8% (99.7–100)	…	…	…	99.7% (99.5–99.9)	…	99.8% (99.5–99.9)	98.2% (97.1–99.3)	99.8% (99.6–100)	99.6% (99.5–99.7)
*P* value for heterogeneity	< 0.001	…	…	…	< 0.001	…	< 0.001	< 0.001	< 0.001	< 0.001
*P* value for difference	…	< 0.001						0.260		
**Stress**										
No. of studies (participant)	22 (110,849)	…	1 (360)	6 (10,431)	6 (28,026)	…	9 (72,032)	5 (6427)	7 (64,164)	10 (40,258)
Prevalence (95% CI)	36.5% (30.0–43.3)	…	65.8% (60.7–70.7)	45.4% (30.0–61.3)	30.4% (22.5–39.0)	…	31.8% (23.7–40.3)	39.7% (24.4–56.1)	40.3% (19.5–63.0)	32.3% (26.2–38.7)
*I*^2^ (95% CI)	99.8% (99.7–100)	…	…	99.6% (99.4–99.8)	99.4% (99.2–99.7)	…	99.6% (99.5–99.7)	99.4% (99.2–99.7)	99.9% (99.7–100)	99.3% (99.1–99.4)
*P* value for heterogeneity	< 0.001	…	…	< 0.001	< 0.001	…	< 0.001	< 0.001	< 0.001	< 0.001
*P* value for difference	…	< 0.001						0.580		
**Psychological distress**										
No. of studies (participant)	18 (81,815)	…	2 (1292)	1 (505)	7 (21,331)	3 (1913)	5 (56,774)	1 (505)	8 (58,437)	9 (22,873)
Prevalence (95% CI)	50.0% (41.8–58.2)	…	80.2% (78.0–82.3)	69.3% (65.1–73.3)	46.0% (33.0–59.2)	57.8% (16.3–93.5)	34.5% (21.9–48.4)	69.3% (65.1–73.3)	50.6% (33.6–67.6)	47.4% (35.4–59.4)
*I*^2^ (95% CI)	99.7% (99.5–100)	…	…	…	99.6% (99.5–99.7)	…	99.5% (99.2–99.7)	…	99.7% (99.5–99.9)	99.6% (99.5–99.7)
*P* value for heterogeneity	< 0.001	…	…	…	< 0.001	…	< 0.001	…	< 0.001	< 0.001
*P* value for difference	…	< 0.001						< 0.001		
**Sleep problems (insomnia/poor sleep)**										
No. of studies (participant)	15 (99,534)	1 (502)	…	1 (4004)	3 (21,820)	…	10 (73,208)	1 (502)	11 (77,212)	3 (21,820)
Prevalence (95% CI)	27.6% (19.8–36.1)	15.1% (12.1–18.6)	…	53.9% (52.3–55.4)	30.0% (5.2–64.3)	…	25.6% (20.2–31.3)	15.1% (12.1–18.6)	28.1% (21.5–35.2)	30.0% (5.2–64.3)
*I*^2^ (95% CI)	99.8% (99.6–100)	…	…	…	…	…	99.2% (99.0–99.4)	…	99.6% (99.3–99.8)	…
*P* value for heterogeneity	< 0.001	…	…	…	…	…		…		…
*P* value for difference	…	< 0.001						< 0.001		

*CI* confidence interval, *WHO* World Health Organisation.

With respect to the small number of included studies and high degree of heterogeneity, the pooled secondary outcome prevalence estimates are presented in Appendix, Table S7. The global prevalence was 16.4% (95% CI 4.8–33.1) for suicide ideation (4 studies^36,41,53,124^, n = 17,554); 53.8% (95% CI 42.4–63.2) for loneliness (3 studies^41,44,45^, n = 2921); 30.7% (95% CI 2.1–73.3) for somatic symptoms (3 studies^53,69,134^, n = 7230); 28.6% (95% CI 9.2–53.6) for low wellbeing (3 studies^53,68,97^, n = 15,737); 50.5% (95% CI 49.2–51.7) for alcohol drinking problems (2 studies^97,114^, n = 6145); 6.4% (95% CI 5.5–7.4) for obsessive–compulsive symptoms (2 studies^73,134^, n = 2535); 25.7% (95% CI 23.7–27.8) for panic disorder (1 study^74^, n = 1753); 2.4% (95% CI 1.6–3.4) for phobia anxiety (1 study^134^, n = 1255); 22.8% (95% CI 22.1–23.4) for adjustment disorder (1 study^80^, n = 18,147); and 1.2% (95% CI 1.0–1.4) for suicide attempts (1 study^36^, n = 10,625).

### Subgroup analyses, sensitivity analyses, meta-regression analyses, and publication bias

In the subgroup analyses (Appendix, Table S8, Table S9, Table S10, Table S1, Table S12), the prevalence of mental health problems was higher in countries with a low to medium HDI (for depression, anxiety, PTSS, and psychological distress), high HDI (for sleep problems), high gender inequality index (for depression and PTSS), very stringent government response index (for PTSS and stress), less stringent government response index (for sleep problems), low to medium hospital beds per 10,000 people (for depression, anxiety, PTSS, stress, psychological distress, and sleep problems), low to medium current health expenditure (for depression, PTSS, and psychological distress), estimated percent change of real GDP growth 2020 below − 3.0 (for psychological distress), low resilience (fourth-quartile) of business environment (for depression, anxiety, and PTSS), medium resilience (second-quartile) of business environment (for psychological distress, and sleep problems), high economic vulnerability-inbound tourism expenditure (for psychological distress, sleep problems), article type-short communication/letter/correspondence (for stress), cross-sectional survey (for PTSS and psychological distress), longitudinal survey (for anxiety and stress), non-mainland China (for depression, anxiety, and psychological distress), sample size of less than 1000 (for psychological distress), sample size of more than 5000 (for PTSS), proportion of females more than 60% (for stress and sleep problems), and measurement tools (for depression, anxiety, stress, and sleep problems). However, several pre-planned subgroup analyses based on participant characteristics and secondary outcomes reported could not be performed due to limited data in the included studies.

Findings from the sensitivity analysis were almost identical to the main analysis (Appendix, Table S14). The pooled prevalence by restricting the analysis to studies with a low risk of bias was 28.6% (95% CI 25.1–32.3) for depression, 27.4% (95% CI 24.1–30.8) for anxiety, 30.2% (95% CI 20.3–41.1) for PTSS, 40.1% (95% CI 32.5–47.9) for stress, 45.4% (95% CI 32.0–59.2) for psychological distress, and 27.7% (95% CI 19.4–36.9) for sleep problems.

On the basis of univariate meta-regression, the analysis was suitable for the primary outcomes (Appendix, Table S15). The increased prevalence of mental health problems was associated with the WHO region (for depression, anxiety, and psychological distress), female gender inequality index (for depression and anxiety), the COVID-19-government response stringency index during the survey (for sleep problems), hospital beds per 10,000 people (for depression and anxiety), immediate economic vulnerability-inbound tourism expenditure (for sleep problems), study design (cross-sectional vs longitudinal survey; for stress), surveyed country (mainland China vs non-mainland China; for depression and psychological distress), and risk of bias (for PTSS).

The visual inspection of the funnel plots, and the p values tested for asymmetry using the Begg’s and Egger’s tests for each prevalence outcome, indicated no evidence of publication bias related to the sample size (Appendix, Table S16, and Figure S2).

## Discussion

This study is, to the best of our knowledge, the first systematic review and meta-analysis on the overall global prevalence of mental health problems and psychosocial consequences among the general population amid the COVID-19 pandemic. Overall, our findings indicate wide variability in the prevalence of mental health problems and psychosocial consequences across countries, particularly in relation to different regions, the global index of COVID-19 pandemic preparedness, inequalities, and economic vulnerabilities indices.

Two reports examined the global prevalence of common mental health disorders among adults prior to the COVID-19 outbreak. The first study was based on 174 surveys across 63 countries from 1980 to 2013. The estimated lifetime prevalence was 29.1% for all mental disorders, 9.6% for mood disorders, 12.9% for anxiety disorders, and 3.4% for substance use disorder^141^. Another report which was conducted as part of the Global Health Estimates by WHO in 2015, showed that the global estimates of depression and anxiety were 4.4% and 3.6% (more common among females than males), respectively^142^. Despite the different methodological methods used, our findings show that the pooled prevalence of mental health problems during the COVID-19 pandemic is higher than before the outbreak.

Previous studies on the prevalence of mental health problems during the COVID-19 pandemic have had substantial heterogeneity. Three systematic reviews reported the prevalence of depression, anxiety, and stress among the general population (mainly in mainland China). The first of these by Salari et al.^11^, was based on 17 included studies (from ten different countries in Asia, Europe, and the Middle East), the pooled prevalence of depression, anxiety, and stress were 33.7% (95% CI 27.5–40.6), 31.9% (95% CI 27.5–36.7), and 29.6% (95% CI 24.3–35.4), respectively. A review by Luo et al.^8^, which included 36 studies from seven different countries, reported a similar overall prevalence of 27% (95% CI 22–33) for depression and 32% (95% CI 25–39) for anxiety. However, a review by Ren et al.^17^, which focussed on only the Chinese population (8 included studies), found that the pooled prevalence was 29% (95% CI 16–42) and 24% (95% CI 16–32), respectively. Nevertheless, previous systematic reviews have been mainly on investigating the prevalence of PTSS, psychological distress, and sleep problems among the patients or healthcare workers that are limited to the general population during the COVID-19 pandemic. With regard to the general population, a review by Cénat et al.^143^, found that the pooled prevalence of PTSS, psychological distress, and insomnia were 22.4% (95% CI 7.6–50.3; 9 included studies), 10.2% (95% CI 4.6–21.0; 10 included studies), and 16.5% (95% CI 8.4–29.7; 8 included studies), respectively.

In this systematic review and meta-analysis, we updated and summarised the global prevalence of mental health problems and psychosocial consequences during the COVID-19 pandemic using information from 32 different countries, and 398,771 participants. A range of problems, including depression, anxiety, PTSS, stress, psychological distress, and sleep problems were reported. The global prevalence of our findings was in line with the previous reviews mentioned above in terms of depression (28.0%; 95% CI 25.0–31.2), anxiety (26.9%; 95% CI 24.0–30.0), and stress (36.5%; 95% CI 30.0–43.3). Interestingly, our findings highlight the poverty impacts of COVID-19 in terms of inequalities, the preparedness of countries to respond, and economic vulnerabilities on the prevalence of mental health problems across countries. For instance, our results suggest that countries with a low or medium HDI had a higher prevalence of depression and anxiety compared to countries with a high or very high HDI (Appendix, Table S8, and Table S9). The prevalence of depression was higher among countries with a gender inequality index of 0.439 or greater (39.6% [95% CI 30.3–49.3] vs 26.2% [95% CI 23.1–29.3]; *P* = 0.020; Appendix, Table S8). Likewise, the prevalence of depression and anxiety was higher among countries with low hospital beds per 10,000 people (Appendix, Table S8, and Table S9). Our findings suggest that the poverty impacts of COVID-19 are likely to be quite significant and related to the subsequent risk of mental health problems and psychosocial consequences. Although we performed a comprehensive review by incorporating articles published together with preprint reports, there was only limited data available on Africa, low-income groups, and secondary outcomes of interest (psychological distress, suicide ideation, suicide attempts, loneliness, somatic symptoms, wellbeing, alcohol drinking problems, obsessive–compulsive symptoms, panic disorder, phobia anxiety, and adjustment disorder).

### Strengths and limitations of this review

From a methodological point of view, we used a rigorous and comprehensive approach to establish an up-to-date overview of the evidence-based information on the global prevalence of mental health problems amid the COVID-19 pandemic, with no language restrictions. The systematic literature search was extensive, comprising published peer-reviewed articles and preprints reporting data to present all relevant literature, minimise bias, and up to date evidence. Our findings expanded and addressed the limitations of the previous systematic reviews, such as having a small sample size and number of included studies, considered more aspects of mental health circumstance, and the generalisability of evidence at a global level^5,6,11,17,18^. To address biases from different measurement tools of assessment and the cultural norms across countries, we summarised the prevalence of mental health problems and psychosocial consequences using a random-effects model to estimate the pooled data with a more conservative approach. Lastly, the sensitivity analyses were consistent with the main findings, suggesting the robustness of our findings. As such, our data can be generalised to individuals in the countries where the included studies were conducted.

There were several limitations to this systematic review and meta-analysis. First, despite an advanced comprehensive search approach, data for some geographical regions according to the WHO regions and World Bank income groups, for instance, the Africa region, as well as the countries in the low-income group, were limited. Moreover, the reporting of key specific outcomes, such as suicide attempts and ideation, alcohol drinking or drug-dependence problems, and stigma towards COVID-19 infection were also limited. Second, a subgroup analysis based on participant characteristics (that is, age, sex, unemployment, history of mental illness, financial problems, and quarantine status), could not be performed as not all of the included studies reported this data. Therefore, the global prevalence of mental health problems and psychosocial consequences amid the COVID-19 pandemic cannot be established. Third, it should be noted that different methods, for example, face-to-face interviews or paper-based questionnaires, may lead to different prevalence estimates across the general population. Due to physical distancing, the included studies in this review mostly used online surveys, which can be prone to information bias and might affect the prevalence estimates of our findings. Fourth, a high degree of heterogeneity between the included studies was found in all outcomes of interest. Even though we performed a set of subgroup analyses concerning the participant characteristics, study characteristics, the global index, and economic indices responses to the COVID-19 pandemic, substantial heterogeneity persisted. However, the univariate meta-regression analysis suggested that the WHO region, gender inequality index, COVID-19-government response stringency index during the survey, hospital beds, immediate economic vulnerability (inbound tourism expenditure), study design, surveyed country (mainland China vs non-mainland China), and risk of bias were associated with an increased prevalence of mental health problems and psychosocial consequences amid the COVID-19 pandemic. Finally, we underline that the diagnostic cut-off criteria used were not uniform across the measurement tools in this review, and misclassification remains possible. The genuine variation in global mental health circumstances across countries cannot be explained by our analyses. Indeed, such variation might be predisposed by social and cultural norms, public resilience, education, ethnic differences, and environmental differences among individual study populations.

### Implications for public health and research

Despite the limitations of our findings, this review provides the best available evidence that can inform the epidemiology of public mental health, implement targeted initiatives, improving screening, and reduce the long-term consequences of the COVID-19 pandemic, particularly among low-income countries, or those with high inequalities, low preparedness, and high economic vulnerability. Our findings could be improved by further standardised methods and measurement tools of assessment. There is a need for individual country-level data on the mental health problems and psychosocial consequences after the COVID-19 pandemic to track and monitor public health responses. There are a number network longitudinal surveys being conducted in different countries that aim to improve our understanding of the long-term effects of the COVID-19 pandemic^144^. To promote mental wellbeing, such initiatives could also be advocated for by public health officials and governments to increase awareness and provide timely proactive interventions in routine practice.

## Conclusions

In conclusion, this systematic review and meta-analysis provides a more comprehensive global overview and evidence of the prevalence of mental health problems among the general population amid the COVID-19 pandemic. The results of this study reveal that the mental health problems and psychosocial consequences amid the COVID-19 pandemic are a global burden, with differences between countries and regions observed. Moreover, equality and poverty impacts were found to be factors in the prevalence of mental health problems. Studies on the long-term effects of the COVID-19 pandemic on the mental health status among the general population at a global level is needed. Given the high burden of mental health problems during the COVID-19 pandemic, an improvement of screening systems and prevention, prompt multidisciplinary management, and research on the social and economic burden of the pandemic, are crucial.

## Data sharing

The data that support the findings of this study are available from the corresponding author upon reasonable request.

## Supplementary Information

Supplementary Figures and Tables.
