# Supplementary material for: Global prevalence of mental health issues among the general population during the coronavirus disease-2019 pandemic: a systematic review and meta-analysis
Source: Sci Rep. 2021 May 13;11:10173. doi: 10.1038/s41598-021-89700-8 (PMC8119461; doi:10.1038/s41598-021-89700-8)

Supplementary Appendix

Global Prevalence of Mental Health Issues Among the General Population During the Coronavirus Disease-2019 Pandemic: A Systematic Review and Meta-Analysis

Surapon Nochaiwong[*], Chidchanok Ruengorn, Kednapa Thavorn, Brian Hutton, Ratanaporn Awiphan, Chabaphai Phosuya, Yongyuth Ruanta, Nahathai Wongpakaran, Tinakon Wongpakaran

[*]**Correspondence and requests for materials:**

Surapon Nochaiwong, PharmD, Department of Pharmaceutical Care, Faculty of Pharmacy, Chiang Mai University, Chiang Mai 50200, Thailand, Phone: 66899973365, Fax: 6653222741, Email: surapon.nochaiwong@gmail.com

Supplementary Online Content

**Table S1**	MOOSE Checklist for Meta-Analyses of Observational Studies	S3
**Table S2**	Systematic Review Search Strategy	S5
**Table S3**	Included Studies in the Systematic Review and Meta-Analysis	S17
**Table S4**	Characteristics of the 107 Included Studies	S23
**Table S5**	Risk of Bias Assessment of Included Studies	S34
**Table S6**	Summary of Primary Outcomes Prevalence Estimates Among the General Population Amid COVID-19 Pandemic, by Countries	S37
**Table S7**	Summary of Secondary Outcomes Prevalence Estimates Among the General Population Amid COVID-19 Pandemic	S43
**Table S8**	Subgroup Analysis of Included Studies: Depression	S44
**Table S9**	Subgroup Analysis of Included Studies: Anxiety	S47
**Table S10**	Subgroup Analysis of Included Studies: Post-Traumatic Stress Symptoms	S50
**Table S11**	Subgroup Analysis of Included Studies: Stress	S53
**Table S12**	Subgroup Analysis of Included Studies: Psychological Distress	S56
**Table S13**	Subgroup Analysis of Included Studies: Sleep Problems	S59
**Table S14**	Sensitivity Analysis by Restricting the Analysis to Studies with a Low Risk of Bias	S62
**Table S15**	Univariable Meta-Regression of Included Studies	S63
**Table S16**	Meta-Analysis of Included Studies with Calibration for Publication Bias	S69
**Figure S1**	Study Selection	S70
**Figure S2**	Funnel Plot of Included Studies in the Meta-Analysis	S71

Table S1 MOOSE Checklist for Meta-Analyses of Observational Studies

Item No	Recommendation	Reported on page No
**Reporting of background should include**		
1	Problem definition	8
2	Hypothesis statement	NA
3	Description of study outcome(s)	11
4	Type of exposure or intervention used	10, 11
5	Type of study designs used	10
6	Study population	10, 11
**Reporting of search strategy should include**		
7	Qualifications of searches (eg, librarians and investigators)	10
8	Search strategy, including time period included in the synthesis and key words	10, Table S2
9	Effort to include all available studies, including contact with authors	10
10	Databases and registries searched	10, Table S2
11	Search software used, name and version, including special features used (eg, explosion)	10, Table S2
12	Use of hand searching (eg, reference lists of obtained articles)	10
13	List of citations located and those excluded, including justification	10, Table S3
14	Method of addressing articles published in languages other than English	10
15	Method of handing abstracts and unpublished studies	10
16	Description of any contact with authors	11, 12
**Reporting of methods should include**		
17	Description of relevance or appropriateness of studies assembled for assessing the hypothesis to be tested	11, 12
18	Rationale for the selection and coding of data (eg, sound clinical principles or convenience)	11, 12
19	Documentation of how data were classified and coded (eg, multiple raters, blinding and interrater reliability)	11, 12
20	Assessment of confounding (eg, comparability of cases and controls in studies where appropriate)	13, 14
21	Assessment of study quality, including blinding of quality assessors, stratification or regression on possible predictors of study results	12
22	Assessment of heterogeneity	13
23	Description of statistical methods (eg, complete description of fixed or random effects models, justification of whether the chosen models account for predictors of study results, dose-response models, or cumulative meta-analysis) in sufficient detail to be replicated	12-14
24	Provision of appropriate tables and graphics	Throughout tables and graphics
**Reporting of results should include**		
25	Graphic summarising individual study estimates and overall estimate	Figure 1 to Figure 6
26	Table giving descriptive information for each study included	Table 1, Table S4

Stroup DF, et al. Meta-analysis of observational studies in epidemiology: a proposal for reporting. Meta-analysis Of Observational Studies in Epidemiology (MOOSE) group. JAMA. 2000;283(15):2008-12.
Abbreviation: NA, not applicable.

Table S1 MOOSE Checklist for Meta-Analyses of Observational Studies (Continued)

Item No	Recommendation	Reported on page No
**Reporting of results should include (continued)**		
27	Results of sensitivity testing (eg, subgroup analysis)	Table S8 to Table S15
28	Indication of statistical uncertainty of findings	Throughout results
**Reporting of discussion should include**		
29	Quantitative assessment of bias (eg, publication bias)	19, Table S16
30	Justification for exclusion (eg, exclusion of non-English language citations)	NA
31	Assessment of quality of included studies	15, 16, Table S5
**Reporting of conclusions should include**		
32	Consideration of alternative explanations for observed results	19-21
33	Generalization of the conclusion(eg, appropriate for the data presented and within the domain of the literature review)	21-23
34	Guidelines for future research	23, 24
35	Disclosure of funding source	25

Stroup DF, et al. Meta-analysis of observational studies in epidemiology: a proposal for reporting. Meta-analysis Of Observational Studies in Epidemiology (MOOSE) group. JAMA. 2000;283(15):2008-12.

Abbreviation: NA, not applicable.

Table S2 Systematic Review Search Strategy: Ovid MEDLINE(R) ALL 1946 to June 12, 2020

Search	Query	Items Found
#1	exp Coronavirus/	16,319
#2	exp Severe Acute Respiratory Syndrome/	4,626
#3	exp Betacoronavirus/	11,251
#4	*Coronavirus, Infection/	8,957
#5	((virus$ or viral or novel or pandemic or epidemic or outbreak or infect*) adj3 (corona* or coronavir* or Wuhan)).ti,ab,kf.	9,981
#6	(coronavir$ or nCoV or COVID-19 or COVID* or SARS* or 2019-nCoV or SARS-CoV-2 or SARS-COV).ti,ab,kf.	41,027
#7	or/1-6	47,411
#8	(public or general public or general population or patients or citizens or people or person or community or healthy or quarantine or isolation or confinement).ti,ab,kf.	7,530,991
#9	(((health* or medical of hospital or allied) and (worker$ or professional or personnel or staff)) or staff or physician* or doctor* or nurse* or dentist* or pharmacist* or psychologist* or therapist*).ti,ab,kf.	1,103,515
#10	or/8-9	8,081,316
#11	7 and 10	18,195
#12	exp Mental Health/	37,830
#13	exp Mental Disorder/	1,232,078
#14	(psychosocial or psychosomatic or unintended consequences or common mental disorder or (mental adj1 health) or (mental adj1 disorder$) or (mental adj1 illness) or (mental adj1 test$) or (mental adj1 problem$) or (psycho* adj1 problem$) or (psycho* adj1 disorder$) or (psycho* adj1 illness$) or (psychiat* adj1 disorder$) or (psychiat* adj1 illness$)).ti,ab,kf.	362,041
#15	exp Depression/	117,944
#16	*Depression, emotion/	71,974
#17	depress*.mp. or depression/	540,869
#18	(depress* or depressive disorder or (depressi* adj3 disorder$) or (depressi* adj3 symptom$) or (depressi* adj3 episode$)).ti,ab,kf.	460,761
#19	exp Anxiety/	84,380
#20	Anxie*.mp. or anxiety/	235,870
#21	(anxiety$ or anxious$).ti,ab,kf.	197,850
#22	((anxi* adj3 disorder$) or (anxi* adj3 symptom$) or generalized anxiety disorder or GAD or social anxiety).ti,ab,kf.	64,097
#23	Stress-related disorder/ or posttraumatic stress disorder/	32,300

Table S2 Systematic Review Search Strategy: Ovid MEDLINE(R) ALL 1946 to June 12, 2020 (Continued)

Search	Query	Items Found
#24	(stress or distress or PTSD or PTSS or (posttraumatic adj1 symptom$) or emotion* trauma or trauma-related disorder$ or traumatic neurosis or mental distress or emotion* distress or motion* stability or (psycho* adj1 distress*) or (psychiat* adj1 distress*)).ti,ab,kf.	852,989
#25	exp Suicide/	62,563
#26	suicide*.mp	84,768
#27	(attempted suicide or suicid$ tendency or suicid$ ideation or self-harm).ti,ab,kf.	20,447
#28	exp Mood Disorder/	121,340
#29	(emotion$ health or emotion$ disturbances or affective or fear or panic* or phobia or sadness).ti,ab,kf.	147,655
#30	exp Insomnia/	13,166
#31	exp Sleep Disorder/	88,889
#32	Insomnia*.mp	21,406
#33	(insomnia or sleep disorder$ or sleep or sleep problem$ or sleep difficulty or asleep or sleep disturbance or awakening or sleepiness or sleep quality).ti,ab,kf.	183,614
#34	or/12-33	2,736,544
#35	11 and 34	2,077
#36	exp Child/	1,899,657
#37	exp Infant	1,133,809
#38	exp Adolescent	2,015,972
#39	(child$ or infant$ or newborn$ or new-born$ or neonat$ or neo-nat$ or baby$ of babies or pediat$ or paediat$ or schoolchild$ or preschool$).ti,ab,kf.	2,084,607
#40	(adolescen$ or juvenile$ or youth$ or teenage$ or youngster$).ti,ab,kf.	430,907
#41	(young people$ or young person$).ti,ab,kf.	30,823
#42	or/36-41	4,245,498
#43	35 not 42	1,803
#44	limit 43 to human	689
#45	(news or newspaper article or interview or review or systematic review or case report or case series).pt.	2,935,692
#46	44 not 45	546
#47	limit 47 to yr="2020-Current"	251

Table S2 Systematic Review Search Strategy: Embase 1980 to 2020 Week 24 (Continued)

Search	Query	Items Found
#1	exp Coronavirus/	14,379
#2	exp Severe Acute Respiratory Syndrome/	8,746
#3	exp Betacoronavirus/	9,389
#4	*Coronavirus, Infection/	2,265
#5	((virus$ or viral or novel or pandemic or epidemic or outbreak or infect*) adj3 (corona* or coronavir* or Wuhan)).ti,ab,kw.	10,281
#6	(coronavir$ or nCoV or COVID-19 or COVID* or SARS* or 2019-nCoV or SARS-CoV-2 or SARS-COV).ti,ab,kw.	40,649
#7	or/1-6	49,287
#8	(public or general public or general population or patients or citizens or people or person or community or healthy or quarantine or isolation or confinement).ti,ab,kw.	10,234,811
#9	(((health* or medical of hospital or allied) and (worker$ or professional or personnel or staff)) or staff or physician* or doctor* or nurse* or dentist* or pharmacist* or psychologist* or therapist*).ti,ab,kw.	1,381,815
#10	or/8-9	10,813,960
#11	7 and 10	19,629
#12	exp Mental Health/	151,624
#13	exp Mental Disorder/	2,082,833
#14	(psychosocial or psychosomatic or unintended consequences or common mental disorder or (mental adj1 health) or (mental adj1 disorder$) or (mental adj1 illness) or (mental adj1 test$) or (mental adj1 problem$) or (psycho* adj1 problem$) or (psycho* adj1 disorder$) or (psycho* adj1 illness$) or (psychiat* adj1 disorder$) or (psychiat* adj1 illness$)).ti,ab,kw.	450,574
#15	exp Depression/	459,794
#16	depress*.mp. or depression/	749,058
#17	(depress* or depressive disorder or (depressi* adj3 disorder$) or (depressi* adj3 symptom$) or (depressi* adj3 episode$)).ti,ab,kw.	591,715
#18	exp Anxiety/	200,531
#19	Anxie*.mp. or anxiety/	354,606
#20	(anxiety$ or anxious$).ti,ab,kw.	279,612
#21	((anxi* adj3 disorder$) or (anxi* adj3 symptom$) or generalized anxiety disorder or GAD or social anxiety).ti,ab,kw.	91,424
#22	Stress-related disorder/ or posttraumatic stress disorder/	59,424
#23	(stress or distress or PTSD or PTSS or (posttraumatic adj1 symptom$) or emotion* trauma or trauma-related disorder$ or traumatic neurosis or mental distress or emotion* distress or motion* stability or (psycho* adj1 distress*) or (psychiat* adj1 distress*)).ti,ab,kw.	1,091,197
#24	exp Suicide/	51,504
#25	suicide*.mp	103,249

Table S2 Systematic Review Search Strategy: Embase 1980 to 2020 Week 24 (Continued)

Search	Query	Items Found
#26	(attempted suicide or suicid$ tendency or suicid$ ideation or self-harm).ti,ab,kw.	25,973
#27	exp Mood Disorder/	501,309
#28	(emotion$ health or emotion$ disturbances or affective or fear or panic* or phobia or sadness).ti,ab,kw.	191,182
#29	exp Insomnia/	65,712
#30	exp Sleep Disorder/	230,692
#31	Insomnia*.mp	72,271
#32	(insomnia or sleep disorder$ or sleep or sleep problem$ or sleep difficulty or asleep or sleep disturbance or awakening or sleepiness or sleep quality).ti,ab,kw.	272,238
#33	or/12-32	3,736,724
#34	11 and 33	2,438
#35	exp Child/	2,432,014
#36	exp Infant	916,631
#37	exp Adolescent	1,456,272
#38	(child$ or infant$ or newborn$ or new-born$ or neonat$ or neo-nat$ or baby$ of babies or pediat$ or paediat$ or schoolchild$ or preschool$).ti,ab,kw.	2,401,439
#39	(adolescen$ or juvenile$ or youth$ or teenage$ or youngster$).ti,ab,kw.	514,766
#40	(young people$ or young person$).ti,ab,kw.	41,533
#41	or/35-40	3,952,084
#42	34 not 41	2,136
#43	limit 42 to human	2,020
#44	(news or newspaper article or interview or review or systematic review or case report or case series).pt.	2,539,938
#45	43 not 44	1,680
#46	limit 45 to yr="2020-Current"	1,039

Table S2 Systematic Review Search Strategy: PsycINFO 1806 to June Week 2, 2020 (Continued)

Search	Query	Items Found
#1	(coronavirus or severe acute respiratory syndrome or betacoronavirus).ti,ab.	413
#2	((virus$ or viral or novel or pandemic or epidemic or outbreak or infect*) adj3 (corona* or coronavir* or Wuhan)).ti,ab.	115
#3	(coronavir$ or nCoV or COVID-19 or COVID* or SARS* or 2019-nCoV or SARS-CoV-2 or SARS-COV).ti,ab.	867
#4	or/1-3	907
#5	(public or general public or general population or patients or citizens or people or person or community or healthy or quarantine or isolation or confinement).ti,ab.	1,387,868
#6	(((health* or medical of hospital or allied) and (worker$ or professional or personnel or staff)) or staff or physician* or doctor* or nurse* or dentist* or pharmacist* or psychologist* or therapist*).ti,ab.	415,996
#7	or/5-6	1,615,246
#8	4 and 7	579
#9	exp Mental Health/	65,851
#10	(psychosocial or psychosomatic or unintended consequences or common mental disorder or (mental adj1 health) or (mental adj1 disorder$) or (mental adj1 illness) or (mental adj1 test$) or (mental adj1 problem$) or (psycho* adj1 problem$) or (psycho* adj1 disorder$) or (psycho* adj1 illness$) or (psychiat* adj1 disorder$) or (psychiat* adj1 illness$)).ti,ab.	371,022
#11	exp Depression/	25,526
#12	*Depression, emotion/	19,742
#13	depress*.mp. or depression/	367,022
#14	(depress* or depressive disorder or (depressi* adj3 disorder$) or (depressi* adj3 symptom$) or (depressi* adj3 episode$)).ti,ab.	299,591
#15	exp Anxiety/	71,200
#16	Anxie*.mp. or anxiety/	240,532
#17	(anxiety$ or anxious$).ti,ab.	198,434
#18	((anxi* adj3 disorder$) or (anxi* adj3 symptom$) or generalized anxiety disorder or GAD or social anxiety).ti,ab.	62,298
#19	Stress-related disorder/ or posttraumatic stress disorder/	32,729
#20	(stress or distress or PTSD or PTSS or (posttraumatic adj1 symptom$) or emotion* trauma or trauma-related disorder$ or traumatic neurosis or mental distress or emotion* distress or motion* stability or (psycho* adj1 distress*) or (psychiat* adj1 distress*)).ti,ab.	260,956
#21	exp Suicide/	34,457
#22	suicide*.mp	59,094
#23	(attempted suicide or suicid$ tendency or suicid$ ideation or self-harm).ti,ab.	19,389
#24	exp Mood Disorder/	142,851
#25	(emotion$ health or emotion$ disturbances or affective or fear or panic* or phobia or sadness).ti,ab.	177,078

Table S2 Systematic Review Search Strategy: PsycINFO 1806 to June Week 2, 2020 (Continued)

Search	Query	Items Found
#26	exp Insomnia/	6,280
#27	exp Sleep Disorder/	19,256
#28	Insomnia*.mp	13,252
#29	(insomnia or sleep disorder$ or sleep or sleep problem$ or sleep difficulty or asleep or sleep disturbance or awakening or sleepiness or sleep quality).ti,ab.	75,446
#30	or/9-29	1,095,428
#31	8 and 30	286
#32	(child$ or infant$ or newborn$ or new-born$ or neonat$ or neo-nat$ or baby$ of babies or pediat$ or paediat$ or schoolchild$ or preschool$).ti,ab.	759,422
#33	(adolescen$ or juvenile$ or youth$ or teenage$ or youngster$).ti,ab.	316,704
#34	(young people$ or young person$).ti,ab.	31,373
#35	or/32-34	959,221
#36	31 not 35	259
#37	limit 36 to human	205
#38	limit 37 to yr="2020-Current"	61

Table S2 Systematic Review Search Strategy: PubMed, From Inception to June 16, 2020 (Continued)

Search	Query	Items Found
#1	((((((((((((Coronavirus) OR Betacoronavirus) OR Coronavirus disease 2019) OR COVID-19) OR COVID) OR Novel coronavirus) OR 2019-nCoV) OR nCoV) OR Severe Acute Respiratory Syndrome) OR SARS) OR SARS-CoV-2) OR SARS-COV) OR Wuhan pneumonia	52,054
#2	((((((((((Public) OR General public) OR General population) OR Patients) OR Citizens) OR People) OR Person) OR Community) OR Healthy) OR Quarantine) OR Isolation) OR Confinement	15,530,812
#3	((((((((((((((Healthcare workers) OR Healthcare professional) OR Healthcare personnel) OR Health professional) OR Health workers) OR Medical workers) OR Practitioner) OR Medical staff) OR Allied staff) OR Physician) OR Doctor) OR Nurse) OR Dentist) OR Pharmacist) OR Psychologist) OR Therapist	3,305,624
#4	#2 OR #3	16,172,297
#5	#1 AND #4	32,615
#6	((((((((Mental) OR Mental Health) OR Mental Disorder) OR Mental illness) OR Mental problems) OR Psychosocial) OR Psychosomatic) OR Unintended consequences) OR Common mental disorder	1,635,801
#7	((((Depression) OR Depressive) OR Depress) OR Depressive disorder) OR Depressive symptoms	518,102
#8	((((Anxiety) OR Anxious) OR Generalized anxiety disorder) OR GAD) OR Social anxiety	256,813
#9	((((((((((Stress) OR Distress) OR Mental distress) OR Emotion distress) OR Stress-related disorder) OR Trauma-related disorder) OR Traumatic neurosis) OR Posttraumatic stress disorder) OR Posttraumatic symptom) OR PTSD) OR PTSS) OR Emotion trauma	1,127,643
#10	((((Suicide) OR Attempted suicide) OR Suicide tendency) OR Suicide ideation) OR Self-harm	108,390
#11	((((((Mood disorder) OR Emotion disturbances) OR Affective) OR Fear) OR Panic) OR Phobia) OR Sadness	314,026
#12	(((((((((Insomnia) OR Sleep) OR Sleep disorder) OR Sleep disturbance) OR Sleep problem) OR Sleep difficulty) OR Sleepiness) OR Sleep quality) OR Asleep) OR Awakening	241,669
#13	#6 OR #7 OR #8 OR #9 OR #10 OR #11 OR #12	3,147,467
#14	#5 AND #13	7,529
#15	((((((((Case Reports[Publication Type]) OR Guideline[Publication Type]) OR News[Publication Type]) OR Newspaper Article[Publication Type]) OR Review[Publication Type])	4,877,143
#16	#14 NOT #15	5,092
#17	Filters: Humans	2,979
#18	Filters: Publication date from Jan 01, 2020 to current	503

Table S2 Systematic Review Search Strategy: The Cochrane Library, From Inception to June 16, 2020 (Continued)

Search	Query	Items Found
#1	(((((((((((Coronavirus) OR Betacoronavirus) OR "Coronavirus disease 2019") OR COVID19) OR COVID) OR "Novel coronavirus") OR 2019nCoV) OR nCoV) OR "Severe Acute Respiratory Syndrome") OR SARS) OR SARS CoV2) OR SARS COV) OR "Wuhan pneumonia"	849
#2	((((((((((Public) OR General public) OR General population) OR Patients) OR Citizens) OR People) OR Person) OR Community) OR Healthy) OR Quarantine) OR Isolation) OR Confinement	1,065,487
#3	((((((((((((((Healthcare workers) OR Healthcare professional) OR Healthcare personnel) OR Health professional) OR Health workers) OR Medical workers) OR Practitioner) OR Medical staff) OR Allied staff) OR Physician) OR Doctor) OR Nurse) OR Dentist) OR Pharmacist) OR Psychologist) OR Therapist	92,172
#4	#2 OR #3	1,084,652
#5	#1 AND #4	742
#6	(((((((Mental) OR Mental Health) OR Mental Disorder) OR Mental illness) OR Mental problems) OR Psychosocial) OR Psychosomatic) OR Unintended consequences) OR Common mental disorder	75,740
#7	((((Depression) OR Depressive) OR Depress) OR Depressive disorder) OR Depressive symptoms	80,359
#8	((((Anxiety) OR Anxious) OR Generalized anxiety disorder) OR GAD) OR Social anxiety	52,391
#9	((((((((((Stress) OR Distress) OR Mental distress) OR Emotion distress) OR Stress related disorder) OR Trauma related disorder) OR Traumatic neurosis) OR Posttraumatic stress disorder) OR Posttraumatic symptom) OR PTSD) OR PTSS) OR Emotion trauma	74,817
#10	((((Suicide) OR Attempted suicide) OR Suicide tendency) OR Suicide ideation) OR Self harm	6,377
#11	((((((Mood disorder) OR Emotion disturbances) OR Affective) OR Fear) OR Panic) OR Phobia) OR Sadness	26,633
#12	(((((((((Insomnia) OR Sleep) OR Sleep disorder) OR Sleep disturbance) OR Sleep problem) OR Sleep difficulty) OR Sleepiness) OR Sleep quality) OR Asleep) OR Awakening	45,538
#13	#6 OR #7 OR #8 OR #9 OR #10 OR #11 OR #12	241,994
#14	#5 AND #13	197
#15	Filters: Library publication date from Jan 2020 to Current	148

Table S2 Systematic Review Search Strategy: Web of Science, From Inception to June 16, 2020 (Continued)

Search	Query	Items Found
#1	TS=(Coronavirus OR Betacoronavirus OR "Coronavirus disease 2019" OR COVID-19 OR COVID OR "Novel coronavirus" OR 2019-nCoV OR nCoV OR "Severe Acute Respiratory Syndrome" OR SARS OR SARS-CoV-2 OR SARS-COV OR "Wuhan pneumonia")	27,240
#2	TS=(Public OR “General public” OR “General population” OR Patients OR Citizens OR People OR Person OR Community OR Healthy OR Quarantine OR Isolation OR Confinement)	7,357,292
#3	TS= (“Healthcare workers” OR “Healthcare professional” OR “Healthcare personnel” OR “Health professional” OR “Health workers” OR “Medical workers” OR Practitioner OR “Medical staff” OR “Allied staff” OR “Allied health” OR Physician OR Doctor OR Nurse OR Dentist OR Pharmacist OR Psychologist OR Therapist)	735,730
#4	#3 OR #2	7,644,297
#5	#4 AND #1	9,994
#6	TS=(Mental OR “Mental Health” OR “Mental Disorder” OR “Mental illness” OR “Mental problems” OR Psychosocial OR Psychosomatic OR “Unintended consequences” OR “Common mental disorder”)	452,802
#7	TS=(Depression OR Depressive OR Depress OR “Depressive disorder” OR “Depressive symptoms”)	469,701
#8	TS=(Anxiety OR Anxious OR “Generalized anxiety disorder” OR GAD OR Social anxiety)	237,985
#9	TS=(Stress OR Distress OR “Mental distress” OR “Emotion distress” OR “Stress-related disorder” OR “Trauma-related disorder” OR “Traumatic neurosis” OR “Posttraumatic stress disorder” OR “Posttraumatic symptom” OR PTSS OR PTSD OR Emotion trauma)	1,581,251
#10	TS=(Suicide OR “Attempted suicide” OR “Suicide tendency” OR “Suicide ideation” OR Self-harm)	62,347
#11	TS=(“Mood disorder” OR “Emotion disturbances” OR Affective OR Fear OR Panic OR Phobia OR Sadness)	197,554
#12	TS=(Insomnia OR Sleep OR “Sleep disorder” OR “Sleep disturbance” OR “Sleep problem” OR “Sleep difficulty” OR Sleepiness OR “Sleep quality” OR Asleep OR Awakening)	205,077
#13	#12 OR #11 OR #10 OR #9 OR #8 OR #7 OR #6	2,613,527
#14	#13 AND #5	1,199
#15	((#13 AND #5)) AND DOCUMENT TYPES: (Article)	885
#16	Filters: Timespan=2020	376

Table S2 Systematic Review Search Strategy: Scopus, From Inception to June 16, 2020 (Continued)

Search	Query	Items Found
#1	(TITLE-ABS-KEY (coronavirus) OR TITLE-ABS-KEY (betacoronavirus) OR TITLE-ABS-KEY (coronavirus AND disease 2019) OR TITLE-ABS-KEY (covid-19) OR TITLE-ABS-KEY (covid) OR TITLE-ABS-KEY (novel AND coronavirus) OR TITLE-ABS-KEY (2019-ncov) OR TITLE-ABS-KEY (ncov) OR TITLE-ABS-KEY (severe AND acute AND respiratory AND syndrome) OR TITLE-ABS-KEY (sars) OR TITLE-ABS-KEY (sars-cov-2) OR TITLE-ABS-KEY (sars-cov) OR TITLE-ABS-KEY (wuhan AND pneumonia))	146,208
#2	(TITLE-ABS-KEY (public) OR TITLE-ABS-KEY (general AND public) OR TITLE-ABS-KEY (general AND population) OR TITLE-ABS-KEY (patients) OR TITLE-ABS-KEY (citizens) OR TITLE-ABS-KEY (people) OR TITLE-ABS-KEY (person) OR TITLE-ABS-KEY (community) OR TITLE-ABS-KEY (healthy) OR TITLE-ABS-KEY (quarantine) OR TITLE-ABS-KEY (isolation) OR TITLE-ABS-KEY (confinement))	14,236,887
#3	(TITLE-ABS-KEY (healthcare AND workers) OR TITLE-ABS-KEY (healthcare AND professional) OR TITLE-ABS-KEY (healthcare AND personnel) OR TITLE-ABS-KEY (health AND professional) OR TITLE-ABS-KEY (health AND workers) OR TITLE-ABS-KEY (medical AND workers) OR TITLE-ABS-KEY (practitioner) OR TITLE-ABS-KEY (medical AND staff) OR TITLE-ABS-KEY (allied AND staff) OR TITLE-ABS-KEY (allied AND health) OR TITLE-ABS-KEY (physician) OR TITLE-ABS-KEY (doctor) OR TITLE-ABS-KEY (nurse) OR TITLE-ABS-KEY (dentist) OR TITLE-ABS-KEY (pharmacist) OR TITLE-ABS-KEY (psychologist) OR TITLE-ABS-KEY (therapist))	2,180,891
#4	#3 OR #2	15,099,757
#5	#4 AND #1	37,087
#6	(TITLE-ABS-KEY (mental) OR TITLE-ABS-KEY (mental AND health) OR TITLE-ABS-KEY (mental AND disorder) OR TITLE-ABS-KEY (mental AND illness) OR TITLE-ABS-KEY (mental AND problems) OR TITLE-ABS-KEY (psychosocial) OR TITLE-ABS-KEY (psychosomatic) OR TITLE-ABS-KEY (unintended AND consequences) OR TITLE-ABS-KEY (common AND mental AND disorder))	1,166,788
#7	(TITLE-ABS-KEY (depression) OR TITLE-ABS-KEY (depressive) OR TITLE-ABS-KEY (depress) OR TITLE-ABS-KEY (depressive AND disorder) OR TITLE-ABS-KEY (depressive AND symptoms))	753,625
#8	(TITLE-ABS-KEY (anxiety) OR TITLE-ABS-KEY (anxious) OR TITLE-ABS-KEY (generalized AND anxiety AND disorder) OR TITLE-ABS-KEY (gad) OR TITLE-ABS-KEY (social AND anxiety))	400,753
#9	(TITLE-ABS-KEY (stress) OR TITLE-ABS-KEY (distress) OR TITLE-ABS-KEY (mental AND distress) OR TITLE-ABS-KEY (emotion AND distress) OR TITLE-ABS-KEY (stress-related AND disorder) OR TITLE-ABS-KEY (trauma-related AND disorder) OR TITLE-ABS-KEY (traumatic AND neurosis) OR TITLE-ABS-KEY (posttraumatic AND stress AND disorder) OR TITLE-ABS-KEY (posttraumatic AND symptom) OR TITLE-ABS-KEY (ptss) OR TITLE-ABS-KEY (ptsd) OR TITLE-ABS-KEY (emotion AND trauma))	2,680,174
#10	(TITLE-ABS-KEY (suicide) OR TITLE-ABS-KEY (attempted AND suicide) OR TITLE-ABS-KEY (suicide AND tendency) OR TITLE-ABS-KEY (suicide AND ideation) OR TITLE-ABS-KEY (self-harm))	128,891

Table S2 Systematic Review Search Strategy: Scopus, From Inception to June 16, 2020 (Continued)

Search	Query	Items Found
#11	(TITLE-ABS-KEY (mood AND disorder) OR TITLE-ABS-KEY (emotion AND disturbances) OR TITLE-ABS-KEY (affective) OR TITLE-ABS-KEY (fear) OR TITLE-ABS-KEY (panic) OR TITLE-ABS-KEY (phobia) OR TITLE-ABS-KEY (sadness))	406,323
#12	(TITLE-ABS-KEY (insomnia) OR TITLE-ABS-KEY (sleep) OR TITLE-ABS-KEY (sleep AND disorder) OR TITLE-ABS-KEY (sleep AND disturbance) OR TITLE-ABS-KEY (sleep AND problem) OR TITLE-ABS-KEY (sleep AND difficulty) OR TITLE-ABS-KEY (sleepiness) OR TITLE-ABS-KEY (sleep AND quality) OR TITLE-ABS-KEY (asleep) OR TITLE-ABS-KEY (awakening))	335,899
#13	#6 OR #7 OR #8 OR #9 OR #10 OR #11 OR #12	4,798,699
#14	#5 AND #13	9,110
#15	Filters: (LIMIT-TO (PUBYEAR , 2020)) AND (LIMIT-TO (DOCTYPE , "ar") OR LIMIT-TO (DOCTYPE , "le") OR LIMIT-TO (DOCTYPE , "ed"))	1,654

Table S2 Systematic Review Search Strategy: CINAHL, From Inception to June 16, 2020 (Continued)

Search	Query	Items Found
#1	AB Coronavirus OR Betacoronavirus OR Coronavirus disease 2019 OR COVID-19 OR COVID OR Novel coronavirus OR 2019-nCoV OR nCoV OR Severe Acute Respiratory Syndrome OR SARS OR SARS-CoV-2 OR SARS-COV OR Wuhan pneumonia	11,838
#2	AB Public OR General public OR General population OR Patients OR Citizens OR People OR Person OR Community OR Healthy OR Quarantine OR Isolation OR Confinement	2,862,464
#3	AB Healthcare workers OR Healthcare professional OR Healthcare personnel OR Health professional OR Health workers OR Medical workers OR Practitioner OR Medical staff OR Allied staff OR Allied health OR Physician OR Doctor OR Nurse OR Dentist OR Pharmacist OR Psychologist OR Therapist	1,197,249
#4	S2 OR S3	3,466,109
#5	S1 AND S4	7,234
#6	AB Mental OR Mental Health OR Mental Disorder OR Mental illness OR Mental problems OR Psychosocial OR Psychosomatic OR Unintended consequences OR Common mental disorder	742,462
#7	AB Depression OR Depressive OR Depress OR Depressive disorder OR Depressive symptoms	154,396
#8	AB Anxiety OR Anxious OR Generalized anxiety disorder OR GAD OR Social anxiety	83,904
#9	AB Stress OR Distress OR Mental distress OR Emotion distress OR Stress-related disorder OR Trauma-related disorder OR Traumatic neurosis OR Posttraumatic stress disorder OR Posttraumatic symptom OR PTSS OR PTSD OR Emotion trauma	192,581
#10	AB Suicide OR Attempted suicide OR Suicide tendency OR Suicide ideation OR Self-harm	34,230
#11	AB Mood disorder OR Emotion disturbances OR Affective OR Fear OR Panic OR Phobia OR Sadness	83,033
#12	AB Insomnia OR Sleep OR Sleep disorder OR Sleep disturbance OR Sleep problem OR Sleep difficulty OR Sleepiness OR Sleep quality OR Asleep OR Awakening	79,760
#13	S6 OR S7 OR S8 OR S9 OR S10 OR S11 OR S12	1,047,001
#14	S5 AND S13	2,610
#15	Limiters - Published Date: 20200101-20201231<br>Expanders - Apply equivalent subjects<br>Source Types - Academic Journals	471

Table S3 Included Studies in the Systematic Review and Meta-Analysis

Author, Country	Title
**Africa Region**	
Olaseni et al, Nigeria	Psychological distress experiences of Nigerians amid COVID-19 pandemic. PsyArXiv May 6 doi:1031234/osfio/9v78y 2020.
Kim et al, South Africa	Evaluating the mental health impacts of the COVID-19 pandemic in urban South Africa: Perceived risk of COVID-19 infection and childhood trauma predict adult depressive symptoms. medRxiv 2020: 2020.06.13.20130120.
**Region of the Americas**	
Filgueiras, Brazil	Factors linked to changes in mental health outcomes among Brazilians in quarantine due to COVID-19. medRxiv 2020: 2020.05.12.20099374.
Hubner, Brazil	Distress among Brazilian university students due to the Covid-19 pandemic: Survey results and reflections. medRxiv 2020: 2020.06.19.20135251.
Sartorao et al, Brazil	Impact of COVID-19 pandemic on mental health of medical students: a cross-sectional study using GAD-7 and PHQ-9 questionnaires. medRxiv 2020: 2020.06.24.20138925.
Zhang et al, Brazil	Mental distress of adults in Brazil during the COVID-19 crisis. medRxiv 2020: 2020.04.18.20070896.
Garcia-Priego et al, Mexico	Anxiety, depression, attitudes, and internet addiction during the initial phase of the 2019 coronavirus disease (COVID-19) epidemic: A cross-sectional study in Mexico. medRxiv 2020: 2020.05.10.20095844.
Bryan et al, U.S.	Associations among state-level physical distancing measures and suicidal thoughts and behaviors among U.S. adults during the early COVID-19 pandemic. PsyArXiv May 29 doi:1031234/osfio/9bpr4 2020.
Kantor et al, U.S.	Mental health outcomes and associations during the coronavirus disease 2019 pandemic: A cross-sectional survey of the US general population. medRxiv 2020: 2020.05.26.20114140.
Killgore et al, U.S.	Loneliness: A signature mental health concern in the era of COVID-19. Psychiatry Res 2020; 290: 113117.
Lan et al, U.S.	Association between SARS-CoV-2 infection, exposure risk and mental health among a cohort of essential retail workers in the United States. medRxiv 2020: 2020.06.08.20125120.
Lee et al, U.S.	Mental health characteristics associated with dysfunctional coronavirus anxiety. Psychol Med 2020: 1-2.
Liu et al, U.S.	Factors associated with depression, anxiety, and PTSD symptomatology during the COVID-19 pandemic: Clinical implications for U.S. young adult mental health. Psychiatry Res 2020; 290: 113172.
Rosenberg et al, U.S.	Depression and loneliness during COVID-19 restrictions in the United States, and their associations with frequency of social and sexual connections. medRxiv 2020: 2020.05.18.20101840.
**South-East Asia Region**	
Banna et al, Bangladesh	The impact of the COVID-19 pandemic on the mental health of the adult population in Bangladesh: A nationwide cross-sectional study. PsyArXiv May 24 doi:1031234/osfio/chw5d 2020.
Islam et al, Bangladesh	Psychological responses during the COVID-19 outbreak among university students in Bangladesh. PsyArXiv June 2 doi:1031234/osfio/cndz7 2020.
Khan et al, Bangladesh	The impact of COVID-19 pandemic on mental health & wellbeing among home-quarantined Bangladeshi students: A cross-sectional pilot study. PsyArXiv May 15 doi:1031234/osfio/97s5r 2020.
Ahmad et al, India	Factors influencing mental health during COVID-19 outbreak: An exploratory survey among Indian population. medRxiv 2020: 2020.05.03.20081380.
Balhara et al, India	Impact of lockdown following COVID-19 on the gaming behavior of college students. Indian J Public Health 2020; 64(Supplement): S172-s6.
Saikarthik et al, India	Assessment of impact of COVID-19 outbreak & lockdown on mental health status & its associated risk and protective factors in adult Indian population. medRxiv 2020: 2020.06.13.20130153.

Abbreviations: U.S., United states.

Table S3 Included Studies in the Systematic Review and Meta-Analysis (Continued)

Author, Country	Title
**South-East Asia Region (Continued)**	
Vidyadhara et al, India	Mental health status among the South Indian pharmacy students during Covid-19 pandemic quarantine period: A cross-sectional study. medRxiv 2020: 2020.05.08.20093708.
Varshney et al, India	Initial psychological impact of COVID-19 and its correlates in Indian Community: An online (FEEL-COVID) survey. PLoS One 2020; 15(5): e0233874.
Sigdel et al, Nepal	Depression, Anxiety and Depression-anxiety comorbidity amid COVID-19 Pandemic: An online survey conducted during lockdown in Nepal. medRxiv 2020: 2020.04.30.20086926.
Nochaiwong et al, Thailand	Mental health circumstances among health care workers and general public under the pandemic situation of COVID-19 (HOME-COVID-19). Medicine (Baltimore). 2020 Jun 26;99(26):e20751.
**European Region**	
Rauschenberg et al, Germany	Social isolation, mental health and use of digital interventions in youth during the COVID-19 pandemic: a nationally representative survey. PsyArXiv published online June 29 DOI:1031234/osfio/v64hf 2020.
Voitsidis et al, Greece	Insomnia during the COVID-19 pandemic in a Greek population. Psychiatry Research 2020; 289.
Hyland et al, Ireland	Anxiety and Depression in the Republic of Ireland During the COVID-19 Pandemic. PsyArXiv April 22 doi:1031234/osfio/8yqxr 2020.
Cellini et al, Italy	Changes in sleep pattern, sense of time and digital media use during COVID-19 lockdown in Italy. Journal of Sleep Research 2020.
Costantini et al, Italy	Italian validation of CoViD-19 Peritraumatic Distress Index and preliminary data in a sample of general population. Riv Psichiatr 2020; 55(3): 145-51.
Forte et al, Italy	COVID-19 pandemic in the Italian population: Validation of a post-traumatic stress disorder questionnaire and prevalence of PTSD symptomatology. Int J Environ Res Public Health 2020; 17(11).
Mazza et al, Italy	A nationwide survey of psychological distress among Italian people during the COVID-19 pandemic: Immediate psychological responses and associated factors. Int J Environ Res Public Health 2020; 17(9).
Moccia et al, Italy	Affective temperament attachment style and the psychological impact of the COVID-19 outbreak: an early report on the Italian general population. Brain, Behavior and Immunity. 2020.
Rossi et al, Italy	COVID-19 pandemic and lockdown measures impact on mental health among the general population in Italy. An N=18147 web-based survey. medRxiv 2020: 2020.04.09.20057802.
Ebrahimi et al, Norway	The mental health impact of non-pharmacological interventions aimed at impeding viral transmission during the COVID-19 pandemic in a general adult population and the factors associated with adherence to these mitigation strategies. PsyArXiv May 9 doi:1031234/osfio/kjzsp 2020.
Moreira et al, Portugal	Protective elements of mental health status during the COVID-19 outbreak in the Portuguese population. medRxiv 2020: 2020.04.28.20080671.
Gómez-Salgado et al, Spain	Related health factors of psychological distress during the COVID-19 pandemic in Spain. Int J Environ Res Public Health 2020; 17(11).
González-Sanguino et al, Spain	Mental health consequences during the initial stage of the 2020 coronavirus pandemic (COVID-19) in Spain. Brain Behav Immun 2020.
Munoz-Navarro et al, Spain	Emotional distress and associated sociodemographic risk factors during the COVID-19 outbreak in Spain. medRxiv 2020: 2020.05.30.20117457.
Odriozola-González et al, Spain	Psychological effects of the COVID-19 outbreak and lockdown among students and workers of a Spanish university. Psychiatry Res 2020; 290: 113108.
Ozamiz-Etxebarria et al, Spain	Psychological symptoms during the two stages of lockdown in response to the COVID-19 outbreak: An investigation in a sample of citizens in Northern Spain. Front Psychol 2020; 11: 1491.

Table S3 Included Studies in the Systematic Review and Meta-Analysis (Continued)

Author, Country	Title
**European Region (Continued)**	
Parrado-González et al, Spain	Covid-19: factors associated with emotional distress and psychological morbidity in Spanish population. Rev Esp Salud Publica 2020; 94.
Hakansson et al, Sweden	Changes in gambling behavior during the COVID-19 pandemic-A web survey study in Sweden. Int J Environ Res Public Health 2020; 17(11).
Özdin et al, Turkey	Levels and predictors of anxiety, depression and health anxiety during COVID-19 pandemic in Turkish society: The importance of gender. Int J Soc Psychiatry 2020: 20764020927051.
Seyahi et al, Turkey	The psychological state and changes in the routine of the patients with rheumatic diseases during the coronavirus disease (COVID-19) outbreak in Turkey: a web-based cross-sectional survey. Rheumatol Int 2020; 40(8): 1229-38.
Fancourt et al, UK	Trajectories of depression and anxiety during enforced isolation due to COVID-19: longitudinal analyses of 59,318 adults in the UK with and without diagnosed mental illness. medRxiv 2020: 2020.06.03.20120923.
Jia et al, UK	Mental health in the UK during the COVID-19 pandemic: early observations. medRxiv 2020: 2020.05.14.20102012.
Kwong et al, UK	Mental health during the COVID-19 pandemic in two longitudinal UK population cohorts. medRxiv 2020: 2020.06.16.20133116.
Levita et al, UK	Impact of COVID-19 on young people aged 13-24 in the UK- preliminary findings. PsyArXiv published online June 30 DOI:1031234/osfio/uq4rn 2020.
Li et al, UK	Prevalence and predictors of general psychiatric disorders and loneliness during COVID-19 in the United Kingdom: Results from the Understanding Society UKHLS. medRxiv 2020: 2020.06.09.20120139.
Shevlin et al, UK	Anxiety, depression, traumatic stress, and COVID-19 related anxiety in the UK general population during the COVID-19 pandemic. PsyArXiv published online April 18 DOI:1031234/osfio/hb6nq 2020.
Smith et al, UK	Factors associated with self-reported anxiety, depression, and general health during the UK lockdown; a cross-sectional survey. medRxiv 2020: 2020.06.23.20137901.
**Eastern Mediterranean Region**	
El-Zoghby et al, Egypt	Impact of the COVID-19 pandemic on mental health and social support among adult Egyptians. J Community Health 2020; 45(4): 689-95.
Afshar Jahanshahi et al, Iran	The distress of Iranian adults during the COVID-19 pandemic - More distressed than the Chinese and with different predictors. Brain Behav Immun 2020.
Chen et al, Iran	The curvilinear relationship between the age of adults and their mental health in Iran after its peak of COVID-19 cases. medRxiv 2020: 2020.06.11.20128132.
Moghanibashi-Mansourieh et al, Iran	Assessing the anxiety level of Iranian general population during COVID-19 outbreak. Asian Journal of Psychiatry. 2020;51.
Al-Tammemi et al, Jordan	Is it just about physical health? An internet-based cross-sectional study exploring the psychological impacts of COVID-19 pandemic on university students in Jordan using Kessler psychological distress scale. medRxiv 2020: 2020.05.14.20102343.
Naser et al, Jordan	Mental health status of the general population, healthcare professionals, and university students during 2019 coronavirus disease outbreak in Jordan: A cross-sectional study. Brain Behav. 2020 Jun 24;e01730. doi: 10.1002/brb3.1730.
Salman et al, Pakistan	Psychological impact of COVID-19 on Pakistani university students and how they are coping. medRxiv 2020: 2020.05.21.20108647.
Alyami et al, Saudi Arabia	Depression and anxiety during 2019 coronavirus disease pandemic in Saudi Arabia: a cross-sectional study. medRxiv 2020: 2020.05.09.20096677.

Abbreviations: UK, United Kingdom.

Table S3 Included Studies in the Systematic Review and Meta-Analysis (Continued)

Author, Country	Title
**Eastern Mediterranean Region (Continued)**	
Joseph et al, Saudi Arabia	The immediate psychological response of the general population in Saudi Arabia during COVID-19 pandemic: a cross-sectional study. medRxiv 2020: 2020.06.19.20135533.
Fekih-Romdhane et al, Tunisia	Prevalence and predictors of PTSD during the COVID-19 pandemic: Findings from a Tunisian community sample. Psychiatry Research 2020; 290.
Saddik et al, UAE	Assessing the influence of parental anxiety on childhood anxiety during the COVID-19 pandemic in the United Arab Emirates. medRxiv 2020: 2020.06.11.20128371.
Saddik et al, UAE	Increased levels of anxiety among medical and non-medical university students during the COVID-19 pandemic in the United Arab Emirates. medRxiv 2020: 2020.05.10.20096933.
**Western Pacific Region**	
Collie et al, Australia	Psychological distress among people losing work during the COVID-19 pandemic in Australia. medRxiv 2020: 2020.05.06.20093773.
Newby et al, Australia	Acute mental health responses during the COVID-19 pandemic in Australia. medRxiv 2020: 2020.05.03.20089961.
Phillipou et al, Australia	Eating and exercise behaviors in eating disorders and the general population during the COVID-19 pandemic in Australia: Initial results from the COLLATE project. Int J Eat Disord 2020.
Stanton et al, Australia	Depression, anxiety and stress during COVID-19: Associations with changes in physical activity, sleep, tobacco and alcohol use in Australian adults. Int J Environ Res Public Health 2020; 17(11).
Ahmed et al, China	Epidemic of COVID-19 in China and associated psychological problems. Asian Journal of Psychiatry. 2020;51.
Cao et al, China	The psychological impact of the COVID-19 epidemic on college students in China. Psychiatry Res. 2020;287:112934.
Chang et al, China	Mental health status and its influencing factors among college students during the epidemic of COVID-19. Nan Fang Yi Ke Da Xue Xue Bao 2020; 40(2): 171-6.
Chen et al, China	Public anxiety and its influencing factors in the initial outbreak of COVID-19. Fudan University Journal of Medical Sciences 2020; 47(3): 385-91.
Elhai et al, China	COVID-19 anxiety symptoms associated with problematic smartphone use severity in Chinese adults. Journal of Affective Disorders 2020; 274: 576-82.
Gao et al, China	Mental health problems and social media exposure during COVID-19 outbreak. PLoS ONE. 2020;15(4).
Guo et al, China	Coping with COVID-19: Exposure to COVID-19 and negative impact on livelihood predict elevated mental health problems in Chinese adults. Int J Environ Res Public Health 2020; 17(11).
Huang et al, China	Generalized anxiety disorder, depressive symptoms and sleep quality during COVID-19 outbreak in China: a web-based cross-sectional survey. Psychiatry Research. 2020;288.
Lei et al, China	Comparison of prevalence and associated factors of anxiety and depression among people affected by versus people unaffected by quarantine during the COVID-19 epidemic in Southwestern China. Med Sci Monit. 2020;26:e924609.
Li et al, China	Insomnia and psychological reactions during the COVID-19 outbreak in China. J Clin Sleep Med 2020.
Li et al, China	Psychological distress among health professional students during the COVID-19 outbreak. Psychol Med 2020: 1-3.
Liang et al, China	The effect of COVID-19 on youth mental health. Psychiatr Q 2020: 1-12.
Liu et al, China	Online mental health survey in a medical college in China during the COVID-19 outbreak. Frontiers in Psychiatry 2020; 11.
Liu et al, China	Psychological status and behavior changes of the public during the COVID-19 epidemic in China. Infect Dis Poverty 2020; 9(1): 58.
Ni et al, China	Mental health, risk factors, and social media use during the COVID-19 epidemic and cordon sanitaire among the community and health professionals in Wuhan, China: Cross-Sectional Survey. JMIR Ment Health 2020; 7(5): e19009.

Abbreviations: UAE, United Arab Emirates.

Table S3 Included Studies in the Systematic Review and Meta-Analysis (Continued)

Author, Country	Title
**Western Pacific Region (Continued)**	
Qiu et al, China[†]	A nationwide survey of psychological distress among Chinese people in the COVID-19 epidemic: implications and policy recommendations. Gen Psychiatr 2020; 33(2): e100213.
Ren et al, China	Letter to the Editor "A longitudinal study on the mental health of general population during the COVID-19 epidemic in China". Brain Behav Immun 2020.
Shi et al, China	Prevalence of and risk factors associated with mental health symptoms among the general population in China during the coronavirus disease 2019 pandemic. JAMA Netw Open 2020; 3(7): e2014053.
Sun et al, China	Prevalence and risk factors of acute posttraumatic stress symptoms during the COVID-19 outbreak in Wuhan, China. medRxiv 2020: 2020.03.06.20032425.
Sun et al, China	Psychiatric symptoms, risk, and protective factors among university students in quarantine during the COVID-19 pandemic in China. medRxiv 2020: 2020.07.03.20144931.
Tan et al, China	Is returning to work during the COVID-19 pandemic stressful? A study on immediate mental health status and psychoneuroimmunity prevention measures of Chinese workforce. Brain Behavior and Immunity. 2020.
Tang et al, China	COVID-19 related depression and anxiety among quarantined respondents. Psychol Health 2020: 1-15.
Tang et al, China	Prevalence and correlates of PTSD and depressive symptoms one month after the outbreak of the COVID-19 epidemic in a sample of home-quarantined Chinese university students. J Affect Disord 2020; 274: 1-7.
Tian et al, China	Psychological symptoms of ordinary Chinese citizens based on SCL-90 during the level I emergency response to COVID-19. Psychiatry Research. 2020;288.
Wang et al, China	Immediate psychological responses and associated factors during the initial stage of the 2019 coronavirus disease (COVID-19) epidemic among the general population in China. Int J Environ Res Public Health. 2020;17(5).
Wang et al, China	Study on the public psychological states and its related factors during the outbreak of coronavirus disease 2019 (COVID-19) in some regions of China. Psychology health & medicine. 2020:1-10.
Ying et al, China	Mental health status among family members of health care workers in Ningbo, China during the Coronavirus Disease 2019 (COVID-19) outbreak: a Cross-sectional Study. medRxiv 2020: 2020.03.13.20033290.
Zhang et al, China	Mental health and psychosocial problems of medical health workers during the COVID-19 epidemic in China. Psychotherapy and psychosomatics. 2020:1-9.
Zhang et al, China	Impact of the COVID-19 pandemic on mental health and quality of life among local residents in Liaoning Province China: A cross-sectional study. Int J Environ Res Public Health. 2020;17(7).
Zhang et al, China	Mental health problems during the COVID-19 pandemics and the mitigation effects of exercise: A longitudinal study of college students in China. Int J Environ Res Public Health 2020; 17(10).
Zhao et al, China	Perceived stress and sleep quality among the non-diseased general public in China during the 2019 coronavirus disease: a moderated mediation model. Sleep Medicine 2020.
Zhao et al, China	Mental health and its influencing factors among self-isolating ordinary citizens during the beginning epidemic of COVID-19. Journal of Loss and Trauma 2020.
Zhou et al, China	Prevalence and socio-demographic correlates of psychological health problems in Chinese adolescents during the outbreak of COVID-19. European Child and Adolescent Psychiatry 2020.
Zhu et al, China	The immediate mental health impacts of the COVID-19 pandemic among people with or without quarantine managements. Brain Behav Immun 2020.

[†]Includes mainland China, Hong Kong, Macau, and Taiwan.

Table S3 Included Studies in the Systematic Review and Meta-Analysis (Continued)

Author, Country	Title
**Western Pacific Region (Continued)**	
Choi et al, Hong Kong	Depression and anxiety in Hong Kong during COVID-19. Int J Environ Res Public Health 2020; 17(10).
Tso et al, Hong Kong	Alarming levels of psychiatric symptoms and the role of loneliness during the COVID-19 epidemic: A case study of Hong Kong. . PsyArXiv published online June 27 DOI:1031234/osfio/wv9y2 2020.
Ueda et al, Japan	Mental health status of the general population during the COVID-19 pandemic: A cross-sectional national survey in Japan. medRxiv 2020: 2020.04.28.20082453.
Dai et al, Malaysia	Health condition and test availability as predictors of adults' mental health during the COVID-19 pandemic. medRxiv 2020: 2020.06.21.20137000.
Sibley et al, New Zealand	Effects of the COVID-19 pandemic and nationwide lockdown on trust, attitudes toward government, and well-being. Am Psychol 2020.
Nguyen et al, Vietnam	Fear of COVID-19 scale-associations of its scores with health literacy and health-related behaviors among medical students. Int J Environ Res Public Health 2020; 17(11).

Table S4 Characteristics of the 107 Included Studies

Author, country	Article type	Sample size	Mean age (SD), year	No. of female (%)	Study design (data collection)	Period of collecting	Outcomes reported	Measurement tool (diagnostic cut-off)
**Africa Region**								
Olaseni et al, Nigeria	Preprint reporting data	502	28.8 (8.2)	225 (44.8)	Cross-sectional study (online survey)	Mar 20 to Apr 19, 2020	Depression (23.5%), anxiety (49.6%), PTSS (42.8%), insomnia (15.1%)	PHQ-9 (≥10); GAD-7 (≥10); IES-R (≥33); ISI (≥15)
Kim et al, South Africa	Preprint reporting data	221	46.3 (12.9)	162 (73.3)	Longitudinal survey (telephone survey)	Late Mar to early May, 2020	Depression (14.5%)	CES-D (≥10)
**Region of the Americas**								
Filgueiras, Brazil	Preprint reporting data	360	37.9 (12.3)	248 (68.9)	Longitudinal survey (online survey)	Mar 20 to Mar 25, 2020	Depression (62.2%), anxiety (51.7%), stress (65.8%)	FDI (≥53.3); STAI (≥36.5 for men, ≥ 43.7 for women); PSS-10 (≥17)
Hubner, Brazil	Preprint reporting data	654	NR	501 (76.6)	Cross-sectional study (online survey)	May 12 to May 18, 2020	Psychological distress (87.9%)	CPDI (≥28)
Sartorao et al, Brazil	Preprint reporting data	340	NR	251 (73.8)	Cross-sectional study (online survey)	May 18 to May 19, 2020	Depression (64.4%), anxiety (46.2%)	PHQ-9 (≥10); GAD-7 (≥10)
Zhang et al, Brazil	Preprint reporting data	638	NR	368 (57.7)	Cross-sectional study (online survey)	Mar 25 to Mar 28, 2020	Psychological distress (70.8%)	CPDI (≥28)
Garcia-Priego et al, Mexico	Preprint reporting data	561	30.7 (10.6)	400 (71.3)	Cross-sectional study (online survey)	Mar 23 to Apr 21, 2020	Depression (27.6%), anxiety (49.9%)	HADS: depression (≥8), anxiety (≥8)
Bryan et al, U.S.	Preprint reporting data	10,625	45.2 (17.0)	5,389 (50.7)	Cross-sectional study (online survey)	Mar 18 to Apr 4, 2020	Depression (19.5%), suicide ideation (4.6%), suicide attempts (1.2%)	PHQ-9 (≥14); suicide ideation and attempts in the past month (yes/no)
Kantor et al, U.S.	Preprint reporting data	1,005	45.0 (16.0)	518 (51.2)	Cross-sectional study (online survey)	Mar 29 to Mar 31, 2020	Depression (23.1%), anxiety (26.3%)	PHQ-9 (≥10); GAD-7 (≥10)
Killgore et al, U.S.	Short communication/ letter to editor/ correspondence	1,013	NR	446 (44.0)	Cross-sectional study (NR)	Apr 9 to Apr 10, 2020	Depression (32.2%), loneliness (43.0%), suicide ideation (17.6%)	PHQ-9 (≥10); UCLA-Loneliness (≥47); suicide ideation (based on Q9 of PHQ-9)
Lan et al, U.S.	Preprint reporting data	99	49.0 (14.1)	49 (47.1)	Cross-sectional study (paper-based survey)	Early May 2020 (NS)	Depression (8.1%), anxiety (24.2%)	PHQ-9 (≥5); GAD-7 (≥5)

Abbreviations: CES-D, Center for Epidemiologic Studies Depression Scale; CPDI, COVID-19 Peri-traumatic Distress Index; DASS-21, Depression, Anxiety and Stress Scale-21; FDI, Filgueiras Depression Inventory; GAD-7, General Anxiety Disorder-7; HADS, Hospital Anxiety and Depression Scale; IES-R, Impact of Event Scale-Revised; ISI, Insomnia Severity Index; NR, not reported; NS, not specified; PHQ-9, Patient Health Questionnaire-9; Perceived Stress Scale-10; PTSS, post-traumatic stress symptoms; STAI, State-Trait Anxiety Inventory; UCLA-Loneliness, University of California, Los Angeles-Loneliness.

Table S4 Characteristics of the 107 Included Studies (Continued)

Author, country	Article type	Sample size	Mean age (SD), year	No. of female (%)	Study design (data collection)	Period of collecting	Outcomes reported	Measurement tool (diagnostic cut-off)
**Region of the Americas (Continued)**								
Lee et al, U.S.	Short communication/ letter to editor/ correspondence	1237	Median 35 (NR)	558 (45.1)	Cross-sectional study (online survey)	Apr 2 2020	Depression (40.3%), anxiety (36.0%)	PHQ-9 (≥10); GAD-7 (≥10)
Liu et al, U.S.	Published article	898	24.5 (NR)	730 (81.3)	Cross-sectional study (online survey)	Apr 13 to May 19, 2020	Depression (43.3%), anxiety (45.4%), PTSS (31.8%), loneliness (61.5%)	PHQ-8 (≥10); GAD-7 (≥10); PCL-5 (≥45), UCLA-Loneliness-3 (≥6)
Rosenberg et al, U.S.	Preprint reporting data	1,010	Median 48 (32-62)	516 (51.1)	Cross-sectional study (online survey)	Apr 10 to Apr 20, 2020	Depression (30.1%), loneliness (54.0%)	CES-D (≥10); UCLA Loneliness-3 (≥4)
**South-East Asia Region**								
Banna et al, Bangladesh	Preprint reporting data	1,427	25.8 (6.8)	407 (28.5)	Cross-sectional study (online survey)	Apr 29 to May 7, 2020	Depression (57.9%), anxiety (33.7%), stress (59.7%)	DASS-21: depression (≥10), anxiety (≥7), stress (≥11)
Islam et al, Bangladesh	Preprint reporting data	3,122	21.4 (2.0)	1,265 (40.5)	Cross-sectional study (online survey)	Apr 11 to Apr 24, 2020	Depression (62.9%), anxiety (63.6%), stress (58.6%)	DASS-21: depression (≥14), anxiety (≥10), stress (≥19)
Khan et al, Bangladesh	Preprint reporting data	505	NR	188 (37.2)	Cross-sectional study (online survey)	Apr 9 to Apr 23, 2020	Depression (46.9%), anxiety (33.3%), stress (28.5%), psychological distress (69.3%)	DASS-21: depression (≥10), anxiety (≥8), stress (≥15); ESD (≥9)
Ahmad et al, India	Preprint reporting data	392	30.3 (9.3)	185 (47.2)	Cross-sectional study (online survey)	Mar 29 to Apr 12, 2020	Anxiety (25.3%)	GAD-7 (≥10)
Balhara et al, India	Published article	128	19.6 (1.9)	76 (59.4)	Cross-sectional study (online survey)	During lockdown, Mar 2020 (NS)	Depression (25.8%), anxiety (17.2%)	PHQ-9 (≥10); GAD-7 (≥10)
Saikarthik et al, India	Preprint reporting data	873	33.6 (12.2)	400 (45.9)	Cross-sectional study (online survey)	May 5 to May 14, 2020	Depression (18.6%), anxiety (25.7%), stress (22.0%)	DASS-21: depression (≥10), anxiety (≥8), stress (≥15)
Vidyadhara et al, India	Preprint reporting data	500	21.2 (1.3)	326 (65.0)	Cross-sectional study (online survey)	Apr 23 to Apr 30, 2020	Depression (42.6%), anxiety (47.0%), stress (32.0%)	DASS-21: depression (≥10), anxiety (≥8), stress (≥15)

Abbreviations: CES-D, Center for Epidemiologic Studies Depression Scale; DASS-21, Depression, Anxiety and Stress Scale-21; ESD, Event-Specific Distress; GAD-7, General Anxiety Disorder-7; PCL-5, Posttraumatic Stress Disorder Checklist for Diagnostic and Statistical Manual of Mental Disorders, 5th; PHQ-8, Patient Health Questionnaire-8; PHQ-9, Patient Health Questionnaire-9; PTSS, post-traumatic stress symptoms; UCLA-Loneliness, University of California, Los Angeles-Loneliness; United States.

Table S4 Characteristics of the 107 Included Studies (Continued)

Author, country	Article type	Sample size	Mean age (SD), year	No. of female (%)	Study design (data collection)	Period of collecting	Outcomes reported	Measurement tool (diagnostic cut-off)
**South-East Asia Region (Continued)**								
Varshney et al, India	Published article	653	41.8 (13.8)	162 (24.8)	Cross-sectional study (online survey)	Mar 26 to Mar 29, 2020	PTSS (18.2%)	IES-R (≥33)
Sigdel et al, Nepal	Preprint reporting data	349	27.8 (NR)	160 (45.8)	Cross-sectional study (online survey)	Apr 9 to Apr 16, 2020	Depression (34.1%), anxiety (31.2%)	PHQ-9 (≥11); GAD-7 (≥10)
Nochaiwong et al, Thailand	Preprint reporting data	4,004	29.1 (10.8)	2619 (65.4)	Longitudinal survey (online survey)	Apr 21 to May 4, 2020	Depression (41.3%), anxiety (22.6%), stress (72.8%), insomnia 53.9%), somatic symptoms (57.9%), low wellbeing (43.7%), suicide ideation (28.4%)	PHQ-9 (≥9); GAD-7 (≥8); PSS-10 (≥14); ISI (≥8); SSS-8 (≥8); WHO-5 Wellbeing (<50); suicide ideation (based on Q9 of PHQ-9)
**European Region**								
Rauschenberg et al, Germany	Preprint reporting data	666	21.3 (2.6)	318 (47.8)	Cross-sectional study (online survey)	May 7 to May 16, 2020	Psychological distress (56.9%)	Kessler-10 (≥20)
Voitsidis et al, Greece	Short communication/ letter to editor/ correspondence	2,363	NR	1,800 (76.2)	Cross-sectional study (online survey)	Apr 10 to 13, 2020	Insomnia (37.6%)	AIS (≥6)
Hyland et al, Ireland	Preprint reporting data	1,041	45 (15.8)	502 (48.2)	Cross-sectional study (online survey)	Mar 31 to Apr 5, 2020	Depression (22.8%), anxiety (20.0%), PTSS (17.7%)	PHQ-9 (≥10); GAD-7 (≥10); ITQ (based on diagnostic criteria)
Cellini et al, Italy	Published article	1,310	23.9 (3.6)	880 (67.2)	Cross-sectional study (online survey)	Mar 24 to 28, 2020	Depression (24.2%), anxiety (32.6%), stress (50.2%), poor sleep quality (52.4%)	DASS-21: depression (≥14), anxiety (≥10), stress (≥19); PSQI (≥5)
Costantini et al, Italy	Published article	329	46.5 (13.6)	191 (58.1)	Cross-sectional study (online survey)	Apr 15 to 24, 2020	PTSS (13.4%), Psychological distress (25.2%)	IES-R (≥50); CPDI (≥28)
Forte et al, Italy	Published article	2,286	29.6 (11.4)	1,706 (74.6)	Cross-sectional study (online survey)	Mar 18 to Mar 31, 2020	PTSS (27.5%)	PCL-5 (based on diagnostic criteria of DSM-5)

Abbreviations: AIS, Athens Insomnia Scale; DASS-21, Depression, Anxiety and Stress Scale-21; GAD-7, General Anxiety Disorder-7; IES-R, Impact of Event Scale-Revised; ISI, Insomnia Severity Index; ITQ, International Trauma Questionnaire; NR, not reported; PCL-5, Posttraumatic Stress Disorder Checklist for Diagnostic and Statistical Manual of Mental Disorders, 5th; PHQ-9, Patient Health Questionnaire-9; PSQI, Pittsburgh Sleep Quality Index; PSS-10, Perceived Stress Scale-10; PTSS, post-traumatic stress symptoms; SSS-8, Somatic Symptoms Scale-8; WHO, World Health Organization.

Table S4 Characteristics of the 107 Included Studies (Continued)

Author, country	Article type	Sample size	Mean age (SD), year	No. of female (%)	Study design (data collection)	Period of collecting	Outcomes reported	Measurement tool (diagnostic cut-off)
**European Region (Continued)**								
Mazza et al, Italy	Published article	2,766	32.9 (13.2)	1,982 (71.6)	Cross-sectional study (online survey)	Mar 18 to Mar 22, 2020	Depression (32.7%), anxiety (18.7%), stress (27.2%)	DASS-21: high/extremely high (NS)
Moccia et al, Italy	Short communication/ letter to editor/ correspondence	500	NR	298 (59.6)	Cross-sectional study (online survey)	Apr 10 to 13, 2020	Psychological distress (38.0%)	Kessler-10 (≥20)
Rossi et al, Italy	Preprint reporting data	18,147	Median 38.0 (IQR 23.0)	14,207 (79.5)	Cross-sectional study (online survey)	Mar 27 to Apr 6, 2020	Depression (17.1%), anxiety (20.6%), PTSS (36.7%), stress (21.7%), insomnia (7.2%), adjustment disorder (22.8%)	PHQ-9 (≥15); GAD-7 (≥15); GPS-PTSS (3/5 of symptoms); PSS-10 (≥31); ISI (≥22); IADQ (NS)
Ebrahimi et al, Norway	Preprint reporting data	10,084	36 (13.5)	7,872 (78.1)	Cross-sectional study (online survey)	Mar 31 to Apr 7, 2020	Depression (30.8%), anxiety (27.6%)	PHQ-9 (≥10); GAD-7 (≥8)
Moreira et al, Portugal	Preprint reporting data	1,280	37.1 (12.1)	1,022 (79.8)	Cross-sectional study (online survey)	Mar 23, 2020 (NS)	Depression (18.8%), anxiety (15.9%), stress (19.7%), obsessive compulsive symptoms (12.4%)	DASS-21: moderate to extremely severe (NS); OCI (≥21)
Gómez-Salgado et al, Spain	Published article	4,180	40.3 (13.2)	3,092 (74.0)	Cross-sectional study (online survey)	Mar 26 to Apr 26, 2020	Psychological distress (72.0%)	GHQ-12 (≥3)
González-Sanguino et al, Spain	Short communication/ letter to editor/ correspondence	3,480	NR	2,610 (75.0)	Cross-sectional study (online survey)	Mar 21 to Mar 28, 2020	Depression (18.7%), anxiety (21.6%), PTSS (15.8%)	PHQ-2 (NS); GAD-2 (NS); PCL-5 (NS)
Munoz-Navarro et al, Spain	Preprint reporting data	1,753	40.4 (12.9)	1,346 (76.8)	Cross-sectional study (online survey)	Mar 26 to Apr 25, 2020	Depression (22.8%), anxiety (20.8%), panic disorder (25.7%)	PHQ-9 (≥10); GAD-7 (≥10); PHQ-PD (based on DSM-4 criteria)

Abbreviations: DASS-21, Depression, Anxiety and Stress Scale-21; GAD-2, General Anxiety Disorder-2; GAD-7, General Anxiety Disorder-7; GHQ-12, General Health Questionnaire-12; GPS-PTSS, Global Psychotrauma Scale-Post-Traumatic Stress Disorder Subscale; HADS, Hospital Anxiety and Depression Scale; IADQ, International Adjustment Disorder Questionnaire; ISI, Insomnia Severity Index; NR, not reported; NS, not specified; OCI, Obsessive-compulsive Inventory; PCL-5, Posttraumatic Stress Disorder Checklist for Diagnostic and Statistical Manual of Mental Disorders, 5th; PHQ-2, Patient Health Questionnaire-2; PHQ-9, Patient Health Questionnaire-9; PHQ-PD, Patient Health Questionnaire-Panic Disorder; PTSS, post-traumatic stress symptoms; UK, United Kingdom; PSS-10, Perceived Stress Scale-10.

Table S4 Characteristics of the 107 Included Studies (Continued)

Author, country	Article type	Sample size	Mean age (SD), year	No. of female (%)	Study design (data collection)	Period of collecting	Outcomes reported	Measurement tool (diagnostic cut-off)
**European Region (Continued)**								
Odriozola-González et al, Spain	Published article	2,530	27.9 (12.4)	1,672 (66.1)	Cross-sectional study (online survey)	Mar 28 to 4 Apr, 2020	Depression (48.1%), anxiety (35.2%), stress (40.3%), PTSS (50.4%)	DASS-21: moderately to extremely severe (NS); IES-R (≥26)
Ozamiz-Etxebarria et al, Spain	Published article	1,993	33.8 (16.6)	1,584 (79.5)	Cross-sectional study (online survey)	T1: Mar 11 to 18, 2020; T2: Apr 2 to Apr 12, 2020	Depression (27.5%), anxiety (26.9%), stress (26.5%)	DASS-21: depression (≥10), anxiety (≥8), stress (≥15)
Parrado-González et al, Spain	Published article	1,596	38.6 (14.9)	1,063 (66.6)	Cross-sectional study (online survey)	Mar 26 to Apr 1, 2020	PTSS (44.3%), psychological distress (48.8%)	IES-R (≥24); GHQ-12 (≥3)
Hakansson et al, Sweden	Published article	2,016	NR	992 (49.2)	Cross-sectional study (online survey)	Apr 24 to May 3, 2020	Psychological distress (44.1%)	Kessler-6 (≥5)
Özdin et al, Turkey	Published article	343	37.2 (10.3)	169 (49.2)	Cross-sectional study (online survey)	Apr 14 to Apr 16, 2020	Depression (23.6%), anxiety (45.2%)	HADS: depression (≥10), anxiety (≥7)
Seyahi et al, Turkey	Published article	917	Median 35.0 (NR)	659 (71.9)	Cross-sectional study (online survey)	Apr 4 to Apr 24, 2020	Depression (40.7%), anxiety (22.0%), PTSS (25.3%)	HADS: depression (≥8), anxiety (≥11); IES-R (≥33)
Fancourt et al, UK	Preprint reporting data	53,328	NR	38,929 (73.0)	Longitudinal survey (online survey)	Mar 21 to May 10, 2020	Depression (31.4%), anxiety (24.4%)	PHQ-9 (≥10); GAD-7 (≥10)
Jia et al, UK	Preprint reporting data	3,097	NR	2,618 (84.5)	Cross-sectional study (online survey)	Apr 3 to Apr 30, 2020	Depression (31.4%), anxiety (26.0%)	PHQ-9 (≥10); GAD-7 (≥10)
Kwong et al, UK	Preprint reporting data	10,659	48.5 (5.8)	5,945 (55.8)	Longitudinal survey (online survey)	Cohort 1: Apr 9 to May 14, 2020; cohort 2: Apr 17 to May 17, 2020	Depression (18.1%), anxiety (24.0%), low wellbeing (13.0%)	PHQ-9 (≥10) or SMFQ (≥11), GAD-7 (≥10), SWEMWBS (≤17)
Levita et al, UK	Preprint reporting data	1,971	NR	1,301 (66.0)	Longitudinal survey (online survey)	Apr 21 to Apr 29, 2020	Depression (34.7%), anxiety (55.6%), PTSS (5.4%), somatic symptoms (54.6%)	HADS: depression (≥10), anxiety (≥12); CRIES-8 (≥17); SSS-8 (≥8)
Li et al, UK	Preprint reporting data	15,530	51.6 (NR)	9,045 (58.2)	Longitudinal survey (online and telephone survey)	Apr 24 to Apr 30, 2020	Depression (18.6%), psychological distress (37.1%), psychological symptoms (29.2%)	GHQ-12: depression (≥6), distress (≥3), psychological symptoms (≥4)

Abbreviations: CRIES-8, Children's Revised Impact of Event Scale-8; DASS-21, Depression, Anxiety and Stress Scale-21; GAD-2, General Anxiety Disorder-2; GAD-7, General Anxiety Disorder-7; GHQ-12, General Health Questionnaire-12; HADS, Hospital Anxiety and Depression Scale; IES-R, Impact of Event Scale-Revised; NR, not reported; NS, not specified; PHQ-2, Patient Health Questionnaire-2; PHQ-9, Patient Health Questionnaire-9; PTSS, post-traumatic stress symptoms; SMFQ, Short Mood and Feelings Questionnaire; SSS-8, Somatic Symptoms Scale-8; SWEMWBS, Short Warwick Edinburgh Mental Well-Being Scale; UK, United Kingdom.

Table S4 Characteristics of the 107 Included Studies (Continued)

Author, country	Article type	Sample size	Mean age (SD), year	No. of female (%)	Study design (data collection)	Period of collecting	Outcomes reported	Measurement tool (diagnostic cut-off)
**European Region (Continued)**								
Shevlin et al, UK	Preprint reporting data	2,025	45.4 (15.9)	1,047 (51.7)	Longitudinal survey (online survey)	Mar 23 to Mar 28, 2020	Depression (22.1%), anxiety (21.6%), PTSS (16.8%)	PHQ-9 (≥10); GAD-7 (≥10); ITQ (based on diagnostic criteria)
Smith et al, UK	Preprint reporting data	2,240	NR	1,164 (52.0)	Cross-sectional study (online survey)	May 6 to May 7, 2020	Depression (23.5%), anxiety (21.9%)	PHQ-2 (≥3); GAD-2 (≥3)
**Eastern Mediterranean Region**								
El-Zoghby et al, Egypt	Published article	510	NR	336 (65.9)	Cross-sectional study (online survey)	May 2 to May 9, 2020	PTSS (52.0%)	IES-R (≥33)
Afshar Jahanshahi et al, Iran	Short communication/ letter to editor/ correspondence	1,058	NR	569 (53.8)	Cross-sectional study (online survey)	Mar 25 to Mar 28, 2020	Psychological distress (61.1%)	CPDI (≥28)
Chen et al, Iran	Preprint reporting data	474	NR	243 (51.3)	Cross-sectional study (online survey)	Apr 1 to Apr 10, 2020	Depression (21.9%), anxiety (21.1%), psychological distress (14.8%)	PHQ-2 (≥3); GAD-2 (≥3); Kessler-6 (≥13)
Moghanibashi-Mansourieh et al, Iran	Published article	10,754	NR	7,073 (65.8)	Cross-sectional study (online survey)	Mar 1 to Mar 9, 2020	Anxiety (50.9%)	DASS-21: anxiety (≥7)
Al-Tammemi et al, Jordan	Preprint reporting data	381	22.6 (3.2)	199 (52.2)	Cross-sectional study (online survey)	May 2020 (NS)	Psychological distress (92.9%)	Kessler-10 (≥20)
Naser et al, Jordan	Published article	2,963	NR	1,783 (60.2)	Cross-sectional study (online survey)	Mar 22 to Mar 28, 2020	Depression (43.6%), anxiety (31.9%)	PHQ-9 (≥10); GAD-7 (≥10)
Salman et al, Pakistan	Preprint reporting data	1,134	21.7 (3.5)	799 (70.5)	Cross-sectional study (online survey)	Apr to May, 2020 (NS)	Depression (45.0%), anxiety (34.0%)	PHQ-9 (≥10); GAD-7 (≥10)
Alyami et al, Saudi Arabia	Preprint reporting data	2,081	NR	677 (32.5)	Cross-sectional study (online survey)	Mar 27 to Apr 27, 2020	Depression (29.4%), anxiety (26.5%)	PHQ-9 (≥10); GAD-7 (≥10)
Joseph et al, Saudi Arabia	Preprint reporting data	584	NR	223 (38.2)	Cross-sectional study (online survey)	Apr 12 to May 10, 2020	Depression (21.9%), anxiety (17.8%), PTSS (51.9%)	PHQ-2 (≥3); GAD-2 (≥3); IES-6 (≥9)

Abbreviations: CPDI, COVID-19 Peri-traumatic Distress Index; DASS-21, Depression, Anxiety and Stress Scale-21; GAD-2, General Anxiety Disorder-2; GAD-7, General Anxiety Disorder-7; IES-R, Impact of Event Scale-Revised; ITQ, International Trauma Questionnaire; NR, not reported; NS, not specified; PHQ-2, Patient Health Questionnaire-2; PHQ-9, Patient Health Questionnaire-9; PTSS, post-traumatic stress symptoms.

Table S4 Characteristics of the 107 Included Studies (Continued)

Author, country	Article type	Sample size	Mean age (SD), year	No. of female (%)	Study design (data collection)	Period of collecting	Outcomes reported	Measurement tool (diagnostic cut-off)
**Eastern Mediterranean Region (Continued)**								
Fekih-Romdhane et al, Tunisia	Short communication/ letter to editor/ correspondence	603	29.2 (10.4)	446 (74.0)	Cross-sectional study (online survey)	Apr 9 to 15, 2020	PTSS (33.0%)	IES-R (≥34)
Saddik et al, UAE	Preprint reporting data	1,469	NR	1,216 (82.8)	Cross-sectional study (online survey)	Mar 24 to May 15, 2020	Anxiety (49.8%)	GAD-7 (≥8)
Saddik et al, UAE	Preprint reporting data	1,385	20.5 (2.3)	994 (71.8)	Cross-sectional study (online survey)	Mar 11 to Mar 21, 2020	Anxiety (17.8%)	GAD-7 (≥10)
**Western Pacific Region (Continued)**								
Collie et al, Australia	Preprint reporting data	551	NR	431 (78.2)	Cross-sectional study (online survey)	Mar 27 to Apr 20, 2020	Psychological distress (77.0%)	Kessler-6 (≥5)
Newby et al, Australia	Preprint reporting data	5,071	NR	4,348 (85.8)	Cross-sectional study (online survey)	Mar 27 to Apr 7, 2020	Depression (46.3%), anxiety (40.8%), stress (38.7%), alcohol drinking problems (52.7%)	DASS-21: depression, anxiety, stress; AUDIT-C (hazardous drinking ≥3 for women and other genders and ≥4 for men)
Phillipou et al, Australia	Published article	5,289	40.6 (13.7)	4,231 (80.0)	Cross-sectional study (online survey)	1 Apr to 4 Apr, 2020	Depression (34.3%), anxiety (31.8%), stress (27.8%)	DASS-21: depression (≥14), anxiety (≥10), stress (≥19)
Stanton et al, Australia	Published article	1,491	50.5 (14.9)	999 (67.4)	Cross-sectional study (online survey)	Apr 9 to 19 Apr, 2020	Depression (26.6%), anxiety (13.5%), stress (18.2%)	DASS-21: depression (≥7), anxiety (≥6), stress (≥10)
Ahmed et al, China	Published article	1,074	33.5 (11.1)	503 (46.8)	Cross-sectional study (online survey)	NR	Depression (37.2%), anxiety (29.1%), low well-being (32.1%), alcohol drinking problems (40.1%)	BDI-II (≥14); BAI (≥8); WEMWBS (<43); AUDIT (≥8)
Cao et al, China	Published article	7,143	NR	4,975 (69.6)	Cross-sectional study (NR)	NR	Anxiety (24.9%)	GAD-7 (NS)
Chang et al, China	Published article	3,881	20	2,447 (63.0)	Cross-sectional study (online survey)	Jan 31 to Feb 3, 2020	Depression (21.2%), anxiety (26.6%)	PHQ-9 (≥5); GAD-7 (≥6)
Chen et al, China	Published article	3,015	32.3 (10.0)†	3,267 (67.7)†	Cross-sectional study (online survey)	Jan 31 to Feb 2, 2020	Anxiety (23.7%)	GAD-7 (≥10)

†Based on mixed population with regard to the entire sample.

Abbreviations: AUDIT, Alcohol Use Disorders Identification Test; AUDIT-C, Alcohol Use Disorders Identification Test-Concise; BAI, Beck Anxiety Inventory; BDI-II, Beck Depression Inventory-II; DASS-21, Depression, Anxiety and Stress Scale-21; GAD-7, General Anxiety Disorder-7; IES-R, Impact of Event Scale-Revised; NR, not reported; PHQ-9, Patient Health Questionnaire-9; PTSS, post-traumatic stress symptoms; ; UAE, United Arab Emirates; WEMWBS, Warwick–Edinburgh Mental Well-being Scale.

Table S4 Characteristics of the 107 Included Studies (Continued)

Author, country	Article type	Sample size	Mean age (SD), year	No. of female (%)	Study design (data collection)	Period of collecting	Outcomes reported	Measurement tool (diagnostic cut-off)
**Western Pacific Region (Continued)**								
Elhai et al, China	Published article	908	40.4 (9.3)	752 (82.8)	Cross-sectional study (online survey)	Feb 24 to Mar 15, 2020	Depression (15.4%), anxiety (10.6%)	DASS-21: depression (≥6); GAD-7 (≥9)
Gao et al, China	Published article	4,576	NR	3,684 (80.5)	Cross-sectional study (online survey)	Jan 31 to Feb 2, 2020	Depression (48.2%), anxiety (22.7%)	WHO-5 Well-Being Index (<13); GAD-7 (≥10)
Guo et al, China	Published article	2,441	NR	1,279 (52.4)	Cross-sectional study (online survey)	Feb 1 to 10 Feb, 2020	Depression (72.6%), PTSS (79.6%), poor sleep quality (20.6%)	CES-D (≥21); PCL-5 (based on diagnostic criteria of DSM-5); PSQI (≥7)
Huang et al, China	Published article	4,986	35.3 (5.6)†	NR	Cross-sectional study (online survey)	Feb 3 to Feb 17, 2020	Depression (20.2%), anxiety (34.9%), poor sleep quality (15.8%)	CES-D (≥29); GAD-7 (≥9); PSQI (≥8)
Lei et al, China	Published article	1,593	32.3 (9.8)	976 (61.3)	Cross-sectional study (online survey)	Early Feb 2020 (NS)	Depression (14.7%), anxiety (8.3%)	Zung-SDS (≥50); Zung-SAS (≥50)
Li et al, China	Short communication/ letter to editor/ correspondence	3,637	34.5 (9.6)	2,291 (63.0)	Cross-sectional study (online survey)	Feb 5 to Feb 19, 2020	Depression (31.2%), anxiety (27.5%), PTSS (17.6%), insomnia (33.7%)	PHQ-9 (≥5); GAD-7 (≥5); IES-R (≥24); ISI (≥8)
Li et al, China	Short communication/ letter to editor/ correspondence	1,442	20 (1.5)	891 (61.8)	Longitudinal survey (online survey)	Feb 7 to Feb 13, 2020	PTSS (11.1%), Psychological distress (26.6%)	IES-R (≥24); Kessler-6 (≥5)
Liang et al, China	Published article	584	NR	361 (61.8)	Cross-sectional study (online survey)	Jan 30, 2020 (NS)	PTSS (14.4%), psychological problems (40.4%)	PCL-5 (≥38); GHQ-12 (≥16)
Liu et al, China	Published article	217	21.7 (1.7)	127 (58.5)	Cross-sectional study (online survey)	Feb 23 to Apr 2, 2020	Depression (11.1%), anxiety (7.4%)	PHQ-9 (≥10) GAD-7 (≥10)
Liu et al, China	Published article	608	NR	357 (58.7)	Cross-sectional study (online survey)	Jan 30 to Feb 3, 2020	Depression (27.1%), anxiety (15.8%), psychological symptoms (7.7%)	Zung-SDS (≥50); STAI (norm-based, NS); SCL-90 (≥160)
Ni et al, China	Published article	1,577	NR	959 (60.8)	Cross-sectional study (online survey)	Feb 18 to Feb 24, 2020	Depression (19.2%), anxiety (23.8%)	PHQ-2 (≥3); GAD-2 (≥3)

Abbreviations: CES-D, Center for Epidemiologic Studies Depression Scale; DASS-21, Depression, Anxiety and Stress Scale-21; GAD-2, General Anxiety Disorder-2; GAD-7, General Anxiety Disorder-7; GHQ-12, NR, not reported; NS, not specified; PCL-5, Posttraumatic Stress Disorder Checklist for Diagnostic and Statistical Manual of Mental Disorders, 5th; PHQ-2, Patient Health Questionnaire-2; PHQ-9, Patient Health Questionnaire-9; PSQI, Pittsburgh Sleep Quality Index; PTSS, post-traumatic stress symptoms; SCL-90-R, Symptom Checklist-90-Revised; STAI, State-Trait Anxiety Inventory; WHO, World Health Organization; Zung-SDS, Zung-Self-Rating Depression Scale.

Table S4 Characteristics of the 107 Included Studies (Continued)

Author, country	Article type	Sample size	Mean age (SD), year	No. of female (%)	Study design (data collection)	Period of collecting	Outcomes reported	Measurement tool (diagnostic cut-off)
**Western Pacific Region (Continued)**								
Qiu et al, China[†]	Short communication/ letter to editor/ correspondence	52,730	NR	34,131 (64.7)	Cross-sectional study (online survey)	Jan 31 to 10 Feb, 2020	Psychological distress (34.4%)	CPDI (≥28)
Ren et al, China	Short communication/ letter to editor/ correspondence	1,172	NR	NR	Cross-sectional study (online survey)	Feb 14 to Mar 29, 2020	Depression (18.8%), anxiety (13.3%), insomnia (7.2%), stress (67.9%), PTSS (7.0%)	PHQ-9 (NS); GAD-7 (NS); PCL-5 (NS); PSS-10 (NS); ISI (NS)
Shi et al, China	Published article	56,679	36.0 (8.2)	29,530 (52.1)	Cross-sectional study (online survey)	Feb 28 to Mar 11, 2020	Depression (10.8%), anxiety (10.4%), stress (24.4%), insomnia (29.2%)	PHQ-9 (≥10); GAD-7 (≥10); ASDS (dissociative ≥9 and cumulative re-experiencing, avoidance, and arousal cluster ≥28; ISI (≥8)
Sun et al, China	Preprint reporting data	2,091	NR	1,272 (60.8)	Cross-sectional study (online survey)	Jan 30 to Feb 3, 2020	PTSS (4.6%)	PCL-5 (≥33)
Sun et al, China	Preprint reporting data	1,912	20.3 (2.1)	1,334 (69.8)	Cross-sectional study (online survey)	Mar 20 to Apr 10, 2020	Depression (15.6%), anxiety (9.6%), PTSS (17.7%), suicide ideation (19.6%)	PHQ-9 (≥10); GAD-7 (≥10); IES-R (≥26); suicide ideation in the past two weeks (yes/no)
Tan et al, China	Published article	673	30.8 (7.4)	172 (25.6)	Cross-sectional study (online survey)	Feb 24 to Feb 25, 2020	Depression (5.9%), anxiety (6.1%), stress (3.3%), PTSS (10.8%), insomnia (14.6%)	DASS-21: NS; IES-R (≥24); ISI (≥8)
Tang et al, China	Published article	1,160	NR	696 (60.0)	Cross-sectional study (online survey)	Feb 5 to Feb 7, 2020	Depression (26.5%), anxiety (70.8%)	CES-D (≥15); GAD-7 (≥5)
Tang et al, China	Published article	2,485	19.8 (1.6)	1,525 (61.4)	Cross-sectional study (online survey)	Feb 20 to Feb 27, 2020	Depression (9.0%), PTSS (2.7%)	PHQ-9 (≥10); PCL-5 (≥38)
Tian et al, China	Published article	1,060	35.0 (12.8)	511 (48.2)	Cross-sectional study (online survey)	Jan 31 to Feb 2, 2020	Psychological distress (12.5%)	SCL-90-R: global symptoms index (≥63)
Wang et al, China	Published article	1,210	NR	814 (67.3)	Cross-sectional study (online survey)	Jan 31 to Feb 2, 2020	Depression (30.3%), anxiety (36.4%), stress (32.1%), PTSS (75.5%)	DASS-21: depression (≥10), anxiety (≥7), stress; IES-R (≥24)

[†]Includes mainland China, Hong Kong, Macau, and Taiwan.

Abbreviations: ASDS, Acute Stress Disorder Scale; CES-D, Center for Epidemiologic Studies Depression Scale; CPDI, COVID-19 Peri-traumatic Distress Index; DASS-21, Depression, Anxiety and Stress Scale-21; GAD-7, General Anxiety Disorder-7; IES-R, Impact of Event Scale-Revised; ISI, Insomnia Severity Index; NR, not reported; NS, not specified; PHQ-9, Patient Health Questionnaire-9; PSS-10, Perceived Stress Scale-10; PTSS, post-traumatic stress symptoms; SCL-90-R, Symptom Checklist-90-Revised.

Table S4 Characteristics of the 107 Included Studies (Continued)

Author, country	Article type	Sample size	Mean age (SD), year	No. of female (%)	Study design (data collection)	Period of collecting	Outcomes reported	Measurement tool (diagnostic cut-off)
**Western Pacific Region (Continued)**								
Wang et al, China	Published article	600	34.0 (12.0)	333 (55.5)	Cross-sectional study (online survey)	Feb 6 to Feb 9, 2020	Depression (17.2%), anxiety (6.3%)	Zung-SDS (≥53); Zung-SAS (≥50)
Ying et al, China	Preprint reporting data	448	38.0† (9.4)	NR	Cross-sectional study (online survey)	Feb 2020 (NS)	Depression (29.0%), anxiety (32.8%)	PHQ-9 (≥5); GAD-7 (≥5)
Zhang et al, China	Published article	1,255	NR	723 (57.6)	Cross-sectional study (online survey)	Feb 19 to Mar 6, 2020	Depression (9.5%), anxiety (45.5%), insomnia (30.5%), somatic symptoms (0.4%), obsessive-compulsive symptoms (2.2%), phobic anxiety (2.4%)	PHQ-2 (≥3); GAD-2 (≥3); ISI (≥9), SCL-90-R: obsessive compulsive (≥2), phobia anxiety (≥2)
Zhang et al, China	Published article	263	37.7 (14.0)	157 (59.7)	Cross-sectional study (online survey)	Jan 28 to Feb 5, 2020	PTSS (7.6%)	IES-R (≥26)
Zhang et al, China	Published article	66	20.7 (2.1)	41 (62.1)	Longitudinal survey (online survey)	Feb 19, Mar 5, and Mar 20, 2020	Depression (22.7%), anxiety (45.5%), stress (28.8%), poor sleep quality (42.4%)	DASS-21: depression (≥10), anxiety (≥8), stress (≥11); PSQI (≥5)
Zhao et al, China	Published article	1,630	29.2 (10.6)	581 (69.6)‡	Cross-sectional study (online survey)	Feb 18 to 25, 2020	Poor sleep quality (36.4%)	PSQI (≥5)
Zhao et al, China	Published article	515	NR	342 (66.4)	Cross-sectional study (online survey)	Jan 26 to Feb 2, 2020	Depression (29.7%), anxiety (14.4%), PTSS (5.6%)	Zung-SDS (≥53); Zung-SAS (≥50); PCL-5 (based on diagnostic criteria of DSM-5)
Zhou et al, China	Published article	8,079	Median 16.0 (NR)	4,326 (53.5)	Cross-sectional study (online survey)	Mar 8 to Mar 15, 2020	Depression (17.4%), anxiety (10.3%)	PHQ-9 (≥10); GAD-7 (≥10)
Zhu et al, China	Short communication/ letter to editor/ correspondence	1,753	NR	1,052 (60.0)	Cross-sectional study (online survey)	Feb 12 to Mar 17, 2020	Depression (22.8%), anxiety (22.4%), psychological symptoms (15.6%)	PHQ-9 (≥5); GAD-7 (≥5); SRQ-20 (≥7)

†Based on mixed population with regard to the entire sample.

‡Based on the complete case analysis.

Abbreviations: DASS-21, Depression, Anxiety and Stress Scale-21; DSM, The Diagnostic and Statistical Manual of Mental Disorders; GAD-2, General Anxiety Disorder-2; GAD-7, General Anxiety Disorder-7; IES-R, Impact of Event Scale-Revised; ISI, Insomnia Severity Index; NR, not reported; PCL-5, Posttraumatic Stress Disorder Checklist for Diagnostic and Statistical Manual of Mental Disorders, 5th; PHQ-2, Patient Health Questionnaire-2; PHQ-9, Patient Health Questionnaire-9; PSQI, Pittsburgh Sleep Quality Index; PSS-10, SCL-90-R, Symptom Checklist-90-Revised; SRQ-20, Self-Reporting Questionnaire-20; Zung-SAS, Zung-Self-Rating Anxiety Scale; Zung-SDS, Zung-Self-Rating Depression Scale.

Table S4 Characteristics of the 107 Included Studies (Continued)

Author, country	Article type	Sample size	Mean age (SD), year	No. of female (%)	Study design (data collection)	Period of collecting	Outcomes reported	Measurement tool (diagnostic cut-off)
**Western Pacific Region (Continued)**								
Choi et al, Hong Kong	Published article	500	47.3 (15.8)	274 (54.8)	Cross-sectional study (online survey)	Apr 24 to May 3, 2020	Depression (19.8%), anxiety (14.0%)	PHQ-9 (≥10); GAD-7 (≥10)
Tso et al, Hong Kong	Preprint reporting data	381	33.4 (10.6)	246 (56.9)	Cross-sectional study (online survey)	Mar 31 to May 30, 2020	Depression (50.4%), anxiety (128.1%), stress (59.6%)	DASS-21: depression (≥14), anxiety (≥10), stress (≥19)
Ueda et al, Japan	Preprint reporting data	2,000	NR	1,008 (50.4)	Cross-sectional study (online survey)	Apr 16 to Apr 18, 2020 (1st round); May 15 to May 17 (2nd round)	Depression (17.4%), anxiety (10.9%)	PHQ-9 (≥10); GAD-7 (≥10)
Dai et al, Malaysia	Preprint reporting data	669	NR	345 (51.6)	Cross-sectional study (online survey)	May 2 to May 8, 2020	Insomnia (38.9%)	AIS (NS)
Sibley et al, New Zealand	Published article	991	51.5 (13.4)	651 (64.9)	Longitudinal survey (online survey)	Mar 26 to Apr 12, 2020	Psychological distress (26.8%)	Kessler-6 (≥13)
Nguyen et al, Vietnam	Published article	5,423	22.0 (2.0)	2,821 (52.0)	Cross-sectional study (online survey)	Apr 7 to Apr 29, 2020	Anxiety (7.7%)	GAD-7 (≥8)

Abbreviations: AIS, Athens Insomnia Scale; DASS-21, Depression, Anxiety and Stress Scale-21; GAD-7, General Anxiety Disorder-7; NR, not reported; PHQ-9, Patient Health Questionnaire-9.

Table S5 Risk of Bias Assessment of Included Studies†

Author, Country	External validity				Internal validity						RoB (Overall)
	Hoy Q1	Hoy Q2	Hoy Q3	Hoy Q4	Hoy Q5	Hoy Q6	Hoy Q7	Hoy Q8	Hoy Q9	Hoy Q10	
**Africa Region**											
Olaseni et al, Nigeria	Yes	No	No	No	Yes	Yes	Yes	Yes	Yes	Yes	3 (Low)
Kim et al, South Africa	No	No	No	No	Yes	Yes	Yes	Yes	Yes	Yes	4 (Moderate)
**Region of the Americas**											
Filgueiras, Brazil	Yes	No	No	No	Yes	Yes	Yes	Yes	Yes	Yes	3 (Low)
Hubner, Brazil	No	No	No	No	Yes	Yes	Yes	Yes	Yes	Yes	4 (Moderate)
Sartorao et al, Brazil	Yes	No	No	Yes	Yes	Yes	Yes	Yes	Yes	Yes	2 (Low)
Zhang et al, Brazil	Yes	No	No	No	Yes	No	Yes	Yes	Yes	Yes	4 (Moderate)
Garcia-Priego et al, Mexico	No	No	No	No	Yes	Yes	Yes	Yes	Yes	Yes	4 (Moderate)
Bryan et al, U.S.	Yes	Yes	Yes	No	Yes	Yes	Yes	Yes	Yes	Yes	1 (Low)
Kantor et al, U.S.	Yes	Yes	Yes	Yes	Yes	No	Yes	Yes	Yes	Yes	1 (Low)
Killgore et al, U.S.	Yes	Yes	No	No	Yes	No	Yes	Yes	Yes	Yes	3 (Low)
Lan et al, U.S.	No	No	No	Yes	Yes	Yes	Yes	Yes	Yes	Yes	3 (Low)
Lee et al, U.S.	No	Yes	No	No	Yes	No	Yes	Yes	Yes	Yes	4 (Moderate)
Liu et al, U.S.	No	No	No	No	Yes	Yes	Yes	Yes	Yes	Yes	4 (Moderate)
Rosenberg et al, U.S.	Yes	No	Yes	No	Yes	Yes	Yes	Yes	Yes	Yes	2 (Low)
**South-East Asia Region**											
Banna et al, Bangladesh	Yes	Yes	No	No	Yes	No	Yes	Yes	Yes	Yes	3 (Low)
Islam et al, Bangladesh	No	Yes	No	Yes	Yes	Yes	Yes	Yes	Yes	Yes	2 (Low)
Khan et al, Bangladesh	No	No	No	No	Yes	Yes	Yes	Yes	Yes	Yes	4 (Moderate)
Ahmad et al, India	Yes	No	No	Yes	Yes	Yes	Yes	Yes	Yes	Yes	2 (Low)
Balhara et al, India	No	No	No	No	Yes	No	Yes	Yes	Yes	Yes	5 (Moderate)
Saikarthik et al, India	Yes	No	No	Yes	Yes	Yes	Yes	Yes	Yes	Yes	2 (Low)
Vidyadhara et al, India	No	No	No	No	Yes	No	Yes	Yes	Yes	Yes	5 (Moderate)
Varshney et al, India	No	No	No	No	Yes	Yes	Yes	Yes	Yes	Yes	4 (Moderate)
Sigdel et al, Nepal	Yes	No	No	Yes	Yes	Yes	Yes	Yes	Yes	Yes	2 (Low)
Nochaiwong et al, Thailand	Yes	Yes	No	Yes	Yes	Yes	Yes	Yes	Yes	Yes	1 (Low)
**European Region**											
Rauschenberg et al, Germany	No	No	Yes	No	Yes	Yes	Yes	Yes	Yes	Yes	3 (Low)
Voitsidis et al, Greece	Yes	Yes	No	Yes	Yes	No	Yes	Yes	Yes	Yes	2 (Low)
Hyland et al, Ireland	Yes	Yes	Yes	No	Yes	Yes	Yes	Yes	Yes	Yes	1 (Low)
Cellini et al, Italy	No	Yes	No	No	Yes	Yes	Yes	Yes	Yes	Yes	3 (Low)
Costantini et al, Italy	Yes	No	No	No	Yes	No	Yes	Yes	Yes	Yes	4 (Moderate)
Forte et al, Italy	Yes	Yes	No	Yes	Yes	Yes	Yes	Yes	Yes	Yes	1 (Low)
Mazza et al, Italy	Yes	Yes	No	Yes	Yes	Yes	Yes	Yes	Yes	Yes	1 (Low)
Moccia et al, Italy	Yes	No	No	No	Yes	Yes	Yes	Yes	Yes	Yes	3 (Low)
Rossi et al, Italy	Yes	Yes	No	No	Yes	Yes	Yes	Yes	Yes	Yes	2 (Low)
Ebrahimi et al, Norway	Yes	Yes	Yes	No	Yes	Yes	Yes	Yes	Yes	Yes	1 (Low)
Moreira et al, Portugal	Yes	Yes	No	No	Yes	Yes	Yes	Yes	Yes	Yes	2 (Low)
Gómez-Salgado et al, Spain	Yes	Yes	No	Yes	Yes	Yes	Yes	Yes	Yes	Yes	1 (Low)
González-Sanguino et al, Spain	Yes	Yes	No	No	Yes	Yes	Yes	Yes	Yes	Yes	2 (Low)
Munoz-Navarro et al, Spain	Yes	No	No	No	Yes	No	Yes	Yes	Yes	Yes	4 (Moderate)
Odriozola-González et al, Spain	No	Yes	No	No	Yes	No	Yes	Yes	Yes	Yes	4 (Moderate)
Ozamiz-Etxebarria et al, Spain	No	Yes	No	No	Yes	Yes	Yes	Yes	Yes	Yes	3 (Low)
Parrado-González et al, Spain	Yes	Yes	No	Yes	Yes	Yes	Yes	Yes	Yes	Yes	1 (Low)
Hakansson et al, Sweden	Yes	Yes	No	No	Yes	Yes	Yes	Yes	Yes	Yes	2 (Low)
Özdin et al, Turkey	Yes	No	No	No	Yes	Yes	Yes	Yes	Yes	Yes	3 (Low)
Seyahi et al, Turkey	No	No	No	No	Yes	Yes	Yes	Yes	Yes	Yes	4 (Moderate)
Fancourt et al, UK	Yes	Yes	No	No	Yes	Yes	Yes	Yes	Yes	Yes	2 (Low)

†Based on the risk of bias in prevalence studies by Hoy et al. J Clin Epidemiol. 2012;65(9):934-9.
Abbreviations: RoB, risk of bias; U.S., United states.

Table S5 Risk of Bias Assessment of Included Studies (Continued)†

Author, Country	External validity				Internal validity						RoB (Overall)
	Hoy Q1	Hoy Q2	Hoy Q3	Hoy Q4	Hoy Q5	Hoy Q6	Hoy Q7	Hoy Q8	Hoy Q9	Hoy Q10	
**European Region (Continued)**											
Jia et al, UK	Yes	Yes	No	Yes	Yes	Yes	Yes	Yes	Yes	Yes	1 (Low)
Kwong et al, UK	Yes	Yes	No	No	Yes	Yes	Yes	No	Yes	Yes	3 (Low)
Levita et al, UK	No	Yes	No	No	Yes	No	Yes	Yes	Yes	Yes	4 (Moderate)
Li et al, UK	Yes	No	No	No	Yes	No	Yes	No	Yes	Yes	5 (Moderate)
Shevlin et al, UK	Yes	Yes	Yes	No	Yes	Yes	Yes	Yes	Yes	Yes	1 (Low)
Smith et al, UK	Yes	Yes	Yes	Yes	Yes	Yes	Yes	Yes	Yes	Yes	0 (Low)
**Eastern Mediterranean Region**											
El-Zoghby et al, Egypt	Yes	No	No	No	Yes	Yes	Yes	Yes	Yes	Yes	3 (Low)
Afshar Jahanshahi et al, Iran	Yes	Yes	No	No	Yes	No	Yes	Yes	Yes	Yes	3 (Low)
Chen et al, Iran	No	No	No	No	Yes	Yes	Yes	Yes	Yes	Yes	4 (Moderate)
Moghanibashi-Mansourieh et al, Iran	Yes	Yes	No	Yes	Yes	No	Yes	Yes	Yes	Yes	2 (Low)
Al-Tammemi et al, Jordan	No	No	No	No	Yes	Yes	Yes	Yes	Yes	Yes	4 (Moderate)
Naser et al, Jordan	Yes	Yes	No	No	Yes	Yes	Yes	Yes	Yes	Yes	2 (Low)
Salman et al, Pakistan	No	Yes	No	No	Yes	Yes	Yes	Yes	Yes	Yes	3 (Low)
Alyami et al, Saudi Arabia	Yes	Yes	No	No	Yes	Yes	Yes	Yes	Yes	Yes	2 (Low)
Joseph et al, Saudi Arabia	Yes	No	No	No	Yes	Yes	Yes	Yes	Yes	Yes	3 (Low)
Fekih-Romdhane et al, Tunisia	Yes	No	No	No	Yes	No	Yes	Yes	Yes	Yes	4 (Moderate)
Saddik et al, UAE	Yes	Yes	No	No	Yes	Yes	Yes	Yes	Yes	Yes	2 (Low)
Saddik et al, UAE	No	Yes	No	Yes	Yes	Yes	Yes	Yes	Yes	Yes	2 (Low)
**Western Pacific Region**											
Collie et al, Australia	No	No	No	No	Yes	Yes	Yes	Yes	Yes	Yes	4 (Moderate)
Newby et al, Australia	Yes	Yes	No	Yes	Yes	Yes	Yes	Yes	Yes	Yes	1 (Low)
Phillipou et al, Australia	Yes	Yes	No	No	Yes	Yes	Yes	Yes	Yes	Yes	2 (Low)
Stanton et al, Australia	Yes	Yes	No	No	Yes	Yes	Yes	Yes	Yes	Yes	2 (Low)
Ahmed et al, China	Yes	Yes	No	No	Yes	Yes	Yes	Yes	Yes	Yes	2 (Low)
Cao et al, China	No	Yes	No	Yes	Yes	Yes	Yes	Yes	Yes	Yes	2 (Low)
Chang et al, China	No	Yes	No	Yes	Yes	Yes	Yes	Yes	Yes	Yes	2 (Low)
Chen et al, China	Yes	Yes	No	No	Yes	Yes	Yes	Yes	Yes	Yes	2 (Low)
Elhai et al, China	No	No	No	No	Yes	No	Yes	Yes	Yes	Yes	5 (Moderate)
Gao et al, China	Yes	Yes	No	Yes	Yes	Yes	Yes	Yes	Yes	Yes	1 (Low)
Guo et al, China	Yes	Yes	Yes	No	Yes	Yes	Yes	Yes	Yes	Yes	1 (Low)
Huang et al, China	Yes	Yes	No	No	Yes	Yes	Yes	Yes	Yes	Yes	2 (Low)
Lei et al, China	Yes	Yes	No	Yes	Yes	Yes	Yes	Yes	Yes	Yes	1 (Low)
Li et al, China	Yes	Yes	No	No	Yes	No	Yes	Yes	Yes	Yes	3 (Low)
Li et al, China	No	Yes	No	No	Yes	No	Yes	Yes	Yes	Yes	4 (Moderate)
Liang et al, China	No	No	No	Yes	Yes	Yes	Yes	Yes	Yes	No	4 (Moderate)
Liu et al, China	No	No	No	No	Yes	No	Yes	Yes	Yes	Yes	5 (Moderate)
Liu et al, China	Yes	No	No	No	Yes	No	Yes	Yes	Yes	Yes	4 (Moderate)
Ni et al, China	Yes	Yes	No	No	Yes	Yes	Yes	Yes	Yes	Yes	2 (Low)
Qiu et al, China‡	Yes	Yes	No	No	Yes	No	Yes	Yes	Yes	Yes	3 (Low)
Ren et al, China	Yes	Yes	No	No	Yes	No	Yes	Yes	Yes	Yes	3 (Low)
Shi et al, China	Yes	Yes	No	Yes	Yes	Yes	Yes	Yes	Yes	Yes	1 (Low)
Sun et al, China	Yes	Yes	No	No	Yes	No	Yes	Yes	Yes	Yes	3 (Low)
Sun et al, China	No	Yes	No	No	Yes	Yes	Yes	Yes	Yes	Yes	3 (Low)
Tan et al, China	No	No	No	No	Yes	Yes	Yes	Yes	Yes	Yes	4 (Moderate)
Tang et al, China	Yes	Yes	No	Yes	Yes	No	Yes	Yes	Yes	Yes	2 (Low)

†Based on the risk of bias in prevalence studies by Hoy et al. J Clin Epidemiol. 2012;65(9):934-9.

‡Includes mainland China, Hong Kong, Macau, and Taiwan.

Abbreviations: RoB, risk of bias; UAE, United Arab Emirates; UK, United Kingdom.

Table S5 Risk of Bias Assessment of Included Studies (Continued)[†]

Author, Country	External validity				Internal validity						RoB (Overall)
	Hoy Q1	Hoy Q2	Hoy Q3	Hoy Q4	Hoy Q5	Hoy Q6	Hoy Q7	Hoy Q8	Hoy Q9	Hoy Q10	
**Western Pacific Region (Continued)**											
Tang et al, China	No	Yes	No	No	Yes	No	Yes	Yes	Yes	Yes	4 (Moderate)
Tian et al, China	Yes	Yes	No	Yes	Yes	No	Yes	Yes	Yes	Yes	2 (Low)
Wang et al, China	Yes	Yes	No	Yes	Yes	No	Yes	Yes	Yes	Yes	2 (Low)
Wang et al, China	Yes	No	No	Yes	Yes	Yes	Yes	Yes	Yes	Yes	2 (Low)
Ying et al, China	No	No	No	Yes	Yes	Yes	Yes	Yes	Yes	Yes	3 (Low)
Zhang et al, China	Yes	Yes	No	No	Yes	Yes	Yes	Yes	Yes	Yes	2 (Low)
Zhang et al, China	Yes	No	No	No	Yes	Yes	Yes	Yes	Yes	Yes	3 (Low)
Zhang et al, China	No	No	No	No	Yes	No	Yes	Yes	Yes	Yes	5 (Moderate)
Zhao et al, China	Yes	Yes	No	No	Yes	Yes	Yes	Yes	Yes	Yes	2 (Low)
Zhao et al, China	No	No	No	No	Yes	No	Yes	Yes	Yes	Yes	5 (Moderate)
Zhou et al, China	No	Yes	No	Yes	Yes	Yes	Yes	Yes	Yes	Yes	2 (Low)
Zhu et al, China	Yes	Yes	No	No	Yes	No	Yes	Yes	Yes	Yes	3 (Low)
Choi et al, Hong Kong	Yes	No	No	No	Yes	Yes	Yes	Yes	Yes	Yes	3 (Low)
Tso et al, Hong Kong	Yes	Yes	No	Yes	Yes	Yes	Yes	Yes	Yes	Yes	1 (Low)
Ueda et al, Japan	Yes	Yes	No	No	Yes	No	Yes	Yes	Yes	Yes	3 (Low)
Dai et al, Malaysia	Yes	No	Yes	No	Yes	No	Yes	Yes	Yes	Yes	3 (Low)
Sibley et al, New Zealand	Yes	No	No	No	Yes	No	Yes	No	Yes	Yes	5 (Moderate)
Nguyen et al, Vietnam	No	Yes	No	No	Yes	No	Yes	Yes	Yes	Yes	4 (Moderate)

[†]Based on the risk of bias in prevalence studies by Hoy et al. J Clin Epidemiol. 2012;65(9):934-9.
Abbreviations: RoB, risk of bias; UAE, United Arab Emirates; UK, United Kingdom; U.S., United states.

Table S6 Summary of Primary Outcomes Prevalence Estimates Among the General Population Amid COVID-19 Pandemic, by Countries: Depression

Country	No. of studies	Cases/Total	Prevalence (95% CI)	I^2 (95% CI)	*P* value for heterogeneity
**Africa region**					
Nigeria	1	118/502	23.5% (19.9-27.5)	NA	NA
South Africa	1	32/221	14.5% (10.1-19.8)	NA	NA
**Region of the Americas**					
Brazil	2	443/700	63.3% (59.7-66.8)	NA	NA
Mexico	1	155/561	27.6% (24.0)	NA	NA
United States	7	3,830/15,887	27.6% (19.8-36.2)	98.8% (98.0-99.7)	<0.001
**South-East Asia Region**					
Bangladesh	3	3,026/5,054	56.2% (48.6-63.7)	NA	NA
India	3	408/1,501	28.5% (13.3-46.7)	NA	NA
Nepal	1	119/349	34.1% (29.1-39.3)	NA	NA
Thailand	1	1,654/4,004	41.3% (39.8-42.8)	NA	NA
**European Region**					
Germany	NA	NA	NA	NA	NA
Greece	NA	NA	NA	NA	NA
Ireland	1	237/1,041	22.8% (20.2-25.4)	NA	NA
Italy	3	4,320/22,223	24.4% (14.5-35.8)	NA	NA
Norway	1	3,104/10,084	30.8% (29.9-31.7)	NA	NA
Portugal	1	240/1,280	18.8% (16.6-21.0)	NA	NA
Spain	4	2,815/9,756	28.7% (16.6-42.7)	99.5% (99.1-99.7)	<0.001
Sweden					
Turkey	2	454/1,260	35.8% (33.2-38.5)	NA	NA
United Kingdom	7	22,680/80,711	25.4% (19.9-31.4)	99.6% (99.5-99.9)	<0.001
**Eastern Mediterranean Region**					
Egypt					
Iran	1	104/474	21.9% (18.3-25.9)	NA	NA
Jordan	1	1,292/2,963	43.6% (41.8-45.4)	NA	NA
Pakistan	1	510/1,134	45.0% (42.1-47.9)	NA	NA
Saudi Arabia	2	740/2,665	27.7% (26.0-29.4)	NA	NA
Tunisia	NA	NA	NA	NA	NA
United Arab Emirates	NA	NA	NA	NA	NA
**Western Pacific Region**					
Australia	3	4,558/11,851	35.6% (25.6-46.3)	NA	NA
China	25	18,100/103,505	22.3% (16.8-28.4)	99.7% (99.5-99.9)	<0.001
Hong Kong	2	291/881	32.2% (29.1-35.3)	NA	NA
Japan	1	347/2,000	17.4% (15.7-19.1)	NA	NA
Malaysia	NA	NA	NA	NA	NA
New Zealand	NA	NA	NA	NA	NA
Vietnam	NA	NA	NA	NA	NA

Abbreviations: CI, confidence interval; COVID-19, coronavirus disease-2019; NA, not applicable.

Table S6 Summary of Primary Outcomes Prevalence Estimates Among the General Population Amid COVID-19 Pandemic, by Countries: Anxiety (Continued)

Country	No. of studies	Cases/Total	Prevalence (95% CI)	I^2 (95% CI)	*P* value for heterogeneity
**Africa region**					
Nigeria	1	249/502	49.6% (45.2-54.0)	NA	NA
South Africa	NA	NA	NA	NA	NA
**Region of the Americas**					
Brazil	2	343/700	49.0% (45.3-52.7)	NA	NA
Mexico	1	280/561	49.9% (45.7-54.1)	NA	NA
United States	4	1,141/3,239	33.2% (24.4-42.6)	96.4% (93.1-98.5)	<0.001
**South-East Asia Region**					
Bangladesh	3	2,634/5,054	43.4% (22.1-66.2)	NA	NA
India	4	580/1,893	28.5% (17.7-40.7)	96.5% (94.1-98.3)	<0.001
Nepal	1	109/349	31.2% (26.4-36.4)	NA	NA
Thailand	1	903/4,004	22.6% (21.3-23.9)	NA	NA
**European Region**					
Germany	NA	NA	NA	NA	NA
Greece	NA	NA	NA	NA	NA
Ireland	1	208/1,041	20.0% (17.6-22.5)	NA	NA
Italy	3	4,675/22,223	23.6% (18.0-29.7)	NA	NA
Norway	1	2,780/10,084	27.6% (26.7-28.4)	NA	NA
Portugal	1	204/1,280	15.9% (14.0-18.1)	NA	NA
Spain	4	2,543/9,756	25.9% (19.7-32.7)	98.2% (97.0-99.2)	<0.001
Sweden	NA	NA	NA	NA	NA
Turkey	2	357/1,260	27.9% (25.4-30.4)	NA	NA
United Kingdom	6	18,368/73,170	28.4% (22.8-34.4)	99.4% (99.1-99.7	
**Eastern Mediterranean Region**					
Egypt	NA	NA	NA	NA	NA
Iran	2	5,572/11,228	49.6% (48.6-50.5)	NA	NA
Jordan	1	944/2,963	31.9% (30.2-33.6)	NA	NA
Pakistan	1	385/1,134	34.0% (31.2-36.8)	NA	NA
Saudi Arabia	2	656/2,665	24.5% (22.9-26.2)	NA	NA
Tunisia	NA	NA	NA	NA	NA
United Arab Emirates	2	978/2,854	33.2% (31.5-35.0)	NA	NA
**Western Pacific Region**					
Australia	3	3,952/11,851	27.9% (16.0-41.6)	NA	NA
China	25	18,159/108,698	22.2% (17.2-27.7)	99.7% (99.5-99.9)	<0.001
Hong Kong	2	177/881	19.6% (17.1-22.3)	NA	NA
Japan	1	218/2,000	10.9% (9.6-12.4)	NA	NA
Malaysia	NA	NA	NA	NA	NA
New Zealand	NA	NA	NA	NA	NA
Vietnam	1	416/5,423	7.7% (7.0-8.4)	NA	NA

Abbreviations: CI, confidence interval; COVID-19, coronavirus disease-2019; NA, not applicable.

Table S6 Summary of Primary Outcomes Prevalence Estimates Among the General Population Amid COVID-19 Pandemic, by Countries: Post-Traumatic Stress Symptoms (Continued)

Country	No. of studies	Cases/Total	Prevalence (95% CI)	I^2 (95% CI)	*P* value for heterogeneity
**Africa region**					
Nigeria	1	215/502	42.8% (38.4-47.3)	NA	NA
South Africa	NA	NA	NA	NA	NA
**Region of the Americas**					
Brazil	NA	NA	NA	NA	NA
Mexico	NA	NA	NA	NA	NA
United States	1	286/898	31.8% (28.8-34.9)	NA	NA
**South-East Asia Region**					
Bangladesh	NA	NA	NA	NA	NA
India	1	119/653	18.2% (15.4-21.3)	NA	NA
Nepal	NA	NA	NA	NA	NA
Thailand	NA	NA	NA	NA	NA
**European Region**					
Germany	NA	NA	NA	NA	NA
Greece	NA	NA	NA	NA	NA
Ireland	1	184/1,041	17.7% (15.4-20.1)	NA	NA
Italy	3	7,339/20,762	25.6% (16.7-35.6)	NA	NA
Norway	NA	NA	NA	NA	NA
Portugal	NA	NA	NA	NA	NA
Spain	3	2,533/7,606	35.9% (14.3-61.0)	NA	NA
Sweden	NA	NA	NA	NA	NA
Turkey	1	232/917	25.3% (22.5-28.2)	NA	NA
United Kingdom	2	446/3,996	10.5% (9.5-11.4)	NA	NA
**Eastern Mediterranean Region**					
Egypt	1	265/510	52.0% (47.5-56.4)	NA	NA
Iran	NA	NA	NA	NA	NA
Jordan	NA	NA	NA	NA	NA
Pakistan	NA	NA	NA	NA	NA
Saudi Arabia	1	277/534	51.9% (47.5-56.2)	NA	NA
Tunisia	1	199/603	33.0% (29.3-36.9)	NA	NA
United Arab Emirates	NA	NA	NA	NA	NA
**Western Pacific Region**					
Australia	NA	NA	NA	NA	NA
China	12	4,447/18,425	18.0% (5.9-34.8)	99.8% (99.6-100)	<0.001
Hong Kong	NA	NA	NA	NA	NA
Japan	NA	NA	NA	NA	NA
Malaysia	NA	NA	NA	NA	NA
New Zealand	NA	NA	NA	NA	NA
Vietnam	NA	NA	NA	NA	NA

Abbreviations: CI, confidence interval; COVID-19, coronavirus disease-2019; NA, not applicable.

Table S6 Summary of Primary Outcomes Prevalence Estimates Among the General Population Amid COVID-19 Pandemic, by Countries: Stress (Continued)

Country	No. of studies	Cases/Total	Prevalence (95% CI)	I^2 (95% CI)	*P* value for heterogeneity
**Africa region**					
Nigeria	NA	NA	NA	NA	NA
South Africa	NA	NA	NA	NA	NA
**Region of the Americas**					
Brazil	1	237/360	65.8% (60.8-70.6)	NA	NA
Mexico	NA	NA	NA	NA	NA
United States	NA	NA	NA	NA	NA
**South-East Asia Region**					
Bangladesh	3	2,824/5,054	48.9% (34.6-63.3)	NA	NA
India	2	352/1,373	25.5% (23.2-27.8)	NA	NA
Nepal	NA	NA	NA	NA	NA
Thailand	1	2,916/4,004	72.8% (71.4-74.2)	NA	NA
**European Region**					
Germany	NA	NA	NA	NA	NA
Greece	NA	NA	NA	NA	NA
Ireland	NA	NA	NA	NA	NA
Italy	3	5,341/22,223	32.4% (19.7-46.7)	NA	NA
Norway	NA	NA	NA	NA	NA
Portugal	1	252/1,280	19.7% (17.5-22.0)	NA	NA
Spain	2	1,548/4,523	34.1% (32.7-35.4)	NA	NA
Sweden	NA	NA	NA	NA	NA
Turkey	NA	NA	NA	NA	NA
United Kingdom	NA	NA	NA	NA	NA
**Eastern Mediterranean Region**					
Egypt	NA	NA	NA	NA	NA
Iran	NA	NA	NA	NA	NA
Jordan	NA	NA	NA	NA	NA
Pakistan	NA	NA	NA	NA	NA
Saudi Arabia	NA	NA	NA	NA	NA
Tunisia	NA	NA	NA	NA	NA
United Arab Emirates	NA	NA	NA	NA	NA
**Western Pacific Region**					
Australia	3	3,704/11,851	27.9% (18.2-38.7)	NA	NA
China	5	15,043/59,800	28.9% (12.6-48.6)	99.7% (99.5-99.9)	<0.001
Hong Kong	1	227/381	59.6% (54.5-64.6)	NA	NA
Japan	NA	NA	NA	NA	NA
Malaysia	NA	NA	NA	NA	NA
New Zealand	NA	NA	NA	NA	NA
Vietnam	NA	NA	NA	NA	NA

Abbreviations: CI, confidence interval; COVID-19, coronavirus disease-2019; NA, not applicable.

Table S6 Summary of Primary Outcomes Prevalence Estimates Among the General Population Amid COVID-19 Pandemic, by Countries: Psychological Distress (Continued)

Country	No. of studies	Cases/Total	Prevalence (95% CI)	I^2 (95% CI)	*P* value for heterogeneity
**Africa region**					
Nigeria	NA	NA	NA	NA	NA
South Africa	NA	NA	NA	NA	NA
**Region of the Americas**					
Brazil	2	1,027/1,292	80.2% (78.0-82.3)	NA	NA
Mexico	NA	NA	NA	NA	NA
United States	NA	NA	NA	NA	NA
**South-East Asia Region**					
Bangladesh	1	350/505	69.3% (65.2-73.3)	NA	NA
India	NA	NA	NA	NA	NA
Nepal	NA	NA	NA	NA	NA
Thailand	NA	NA	NA	NA	NA
**European Region**					
Germany	1	379/666	56.9% (53.1-60.7)	NA	NA
Greece	NA	NA	NA	NA	NA
Ireland	NA	NA	NA	NA	NA
Italy	2	273/829	32.8% (29.6-36.0)	NA	NA
Norway	NA	NA	NA	NA	NA
Portugal	NA	NA	NA	NA	NA
Spain	2	3,788/5,776	65.9% (64.6-67.1)	NA	NA
Sweden	1	875/1,986	44.1% (41.9-46.3)	NA	NA
Turkey	NA	NA	NA	NA	NA
United Kingdom	1	4,479/12,074	37.1% (36.2-38.0)	NA	NA
**Eastern Mediterranean Region**					
Egypt	NA	NA	NA	NA	NA
Iran	2	716/1,532	45.6% (43.1-48.1)	NA	NA
Jordan	1	354/381	92.9% (89.9-95.3)	NA	NA
Pakistan	NA	NA	NA	NA	NA
Saudi Arabia	NA	NA	NA	NA	NA
Tunisia	NA	NA	NA	NA	NA
United Arab Emirates	NA	NA	NA	NA	NA
**Western Pacific Region**					
Australia	1	424/551	77.0% (73.2-80.4)	NA	NA
China	3	18,671/55,232	23.9% (12.6-37.4)	NA	NA
Hong Kong	NA	NA	NA	NA	NA
Japan	NA	NA	NA	NA	NA
Malaysia	NA	NA	NA	NA	NA
New Zealand	1	266/991	26.8% (24.1-29.7)	NA	NA
Vietnam	NA	NA	NA	NA	NA

Abbreviations: CI, confidence interval; COVID-19, coronavirus disease-2019; NA, not applicable.

Table S6 Summary of Primary Outcomes Prevalence Estimates Among the General Population Amid COVID-19 Pandemic, by Countries: Sleep Problems (Continued)

Country	No. of studies	Cases/Total	Prevalence (95% CI)	I^2 (95% CI)	*P* value for heterogeneity
**Africa region**					
Nigeria	1	76/502	15.1% (12.1-18.4)	NA	NA
South Africa	NA	NA	NA	NA	NA
**Region of the Americas**					
Brazil	NA	NA	NA	NA	NA
Mexico	NA	NA	NA	NA	NA
United States	NA	NA	NA	NA	NA
**South-East Asia Region**					
Bangladesh	NA	NA	NA	NA	NA
India	NA	NA	NA	NA	NA
Nepal	NA	NA	NA	NA	NA
Thailand	1	2,157/4,004	53.9% (52.3-55.4)	NA	NA
**European Region**					
Germany	NA	NA	NA	NA	NA
Greece	1	888/2,363	37.6% (35.6-39.6)	NA	NA
Ireland	NA	NA	NA	NA	NA
Italy	2	1,992/19,457	9.2% (8.8-9.6)	NA	NA
Norway	NA	NA	NA	NA	NA
Portugal	NA	NA	NA	NA	NA
Spain	NA	NA	NA	NA	NA
Sweden	NA	NA	NA	NA	NA
Turkey	NA	NA	NA	NA	NA
United Kingdom	NA	NA	NA	NA	NA
**Eastern Mediterranean Region**					
Egypt	NA	NA	NA	NA	NA
Iran	NA	NA	NA	NA	NA
Jordan	NA	NA	NA	NA	NA
Pakistan	NA	NA	NA	NA	NA
Saudi Arabia	NA	NA	NA	NA	NA
Tunisia	NA	NA	NA	NA	NA
United Arab Emirates	NA	NA	NA	NA	NA
**Western Pacific Region**					
Australia	NA	NA	NA	NA	NA
China	9	20,264/72,539	24.2% (18.6-30.2)	99.3% (99.0-99.4)	<0.001
Hong Kong	NA	NA	NA	NA	NA
Japan	NA	NA	NA	NA	NA
Malaysia	1	260/669	25.6% (20.2-31.3)	NA	NA
New Zealand	NA	NA	NA	NA	NA
Vietnam	NA	NA	NA	NA	NA

Abbreviations: CI, confidence interval; COVID-19, coronavirus disease-2019; NA, not applicable.

Table S7 Summary of Secondary Outcomes Prevalence Estimates Among the General Population Amid COVID-19 Pandemic

Outcomes	No. of studies	Cases/Total	Prevalence (95% CI)	I^2 (95% CI)	*P* value for heterogeneity
Suicide ideation	4	2,179/17,554	16.4% (4.8-33.1)	99.8% (99.5-100)	<0.001
Loneliness	3	1,533/2,921	53.8% (42.4-63.2)	97.0% (94.1-99.3)	<0.001
Somatic symptoms	3	3,400/7,230	30.7% (2.1-73.3)	99.9% (99.7-100)	<0.001
Low wellbeing	3	3,481/15,737	28.6% (9.2-53.6)	99.9% (99.7-100)	<0.001
Alcohol drinking problems	2	3,103/6,145	50.5% (49.2-51.7)	NA	NA
Obsessive compulsive symptoms	2	187/2,535	6.4% (5.5-7.4)	NA	NA
Panic disorder	1	451/1,753	25.7% (23.7-27.8)	NA	NA
Phobia anxiety	1	30/1,255	2.4% (1.6-3.4)	NA	NA
Adjustment disorder	1	4,129/18,147	22.8% (22.1-23.4)	NA	NA
Suicide attempts	1	128/10,625	1.2% (1.0-1.4)	NA	NA

Abbreviations: CI, confidence interval; COVID-19, coronavirus disease-2019; NA, not applicable.

Table S8 Subgroup Analysis of Included Studies: Depression

Subgroup comparison	No. of studies	Cases/Total	Prevalence (95% CI)	I^2 (95% CI)	*P* value for heterogeneity	*P* value for difference
HDI groups, 2018†						
Low	1	118/502	23.5% (19.9-27.5)	NA	NA	0.020
Medium	8	4,063/8,038	41.4% (30.0-53.4)	99.1% (98.9-99.3)	<0.001	
High	32	21,780/112,428	25.6% (20.1-31.5)	99.7% (99.5-99.9)	<0.001	
Very high	34	43,616/159,639	27.6% (24.6-30.5)	99.3% (99.1-99.4)	<0.001	
GII, 2018†						
<0.439	62	63,709/267,749	26.2% (23.1-29.3)	99.7% (99.5-99.9)	<0.001	0.020
≥0.439	10	5,459/11,475	39.6% (30.3-49.3)	99.0% (98.8-99.2)	<0.001	
CSI during the survey‡						
Less stringent (<75)	15	6,212/26,417	28.2 (23.4-33.2)	98.5% (97.7-99.2)	<0.001	0.410
Moderate stringent (75-85)	42	50,597/211,872	25.3 (21.4-29.5)	99.7% (99.5-99.9)	<0.001	
Very stringent (>85)	17	12,369/41,244	34.5% (26.2-43.2)	99.6% (99.5-99.8)	<0.001	
Hospital beds per 10,000 people, 2010-2018§						
Low	8	4,063/8,038	41.4% (30.0-53.4)	99.1% (98.7-99.4)		<0.001
Medium-low	3	1,551/3,998	30.8% (17.7-45.8)	NA	NA	
Medium	15	26,208/90,381	31.7% (27.1-36.4)	99.3% (99.2-99.4)	<0.001	
Medium-high	44	36,967/174,586	27.8% (20.9-28.9)	99.7% (99.5-99.9)	<0.001	
High	1	347/2,000	17.4% (15.7-19.1)	NA	NA	
No data	4	441/1,604	26.2% (13.4-41.5)	97.7% (95.7-99.1)	<0.001	

†Based on the 2019 Human Development Report by the United Nations Development Programme.

‡Based on the Oxford COVID-19 Government Response Tracker—highest value during the surveys.

§Based on the 2020 global preparedness and vulnerability to respond to COVID-19 pandemic by the United Nations Development Programme.

Abbreviations: CI, confidence interval; CSI, Coronavirus Disease-2019 Government Response Stringency Index; GDP, gross domestic product; GII, Gender Inequality Index; HDI, Human Development Index; NA, not applicable.

Table S8 Subgroup Analysis of Included Studies: Depression (Continued)

Subgroup comparison	No. of studies	Cases/Total	Prevalence (95% CI)	I^2 (95% CI)	*P* value for heterogeneity	*P* value for difference
Current health expenditure (% of GDP), 2016§						
Low	9	5,716/12,195	40.2% (30.4-50.3)	99.1% (98.8-99.3)	<0.001	0.020
Medium-low	29	20,001/108,289	23.8% (18.4-29.7)	99.7% (99.5-99.9)	<0.001	
Medium	3	859/3,014	28.2% (22.4-34.4)	NA	NA	
Medium-high	10	7,508/33,715	24.7% (18.6-31.3)	99.3% (99.1-99.4)	<0.001	
High	22	35,202/122,513	30.1% (26.5-33.9)	99.3% (99.0-99.4)	<0.001	
Real GDP growth 2020, estimates percent change¥						
Below -3.0	40	46,674/166,399	29.4% (26.4-32.4)	99.3% (99.2-99.4)	<0.001	0.410
Above -3.0	35	22,903/114,208	26.5% (20.8-32.7)	99.6% (99.5-99.8)	<0.001	
Resilience of a country's business environment£						
First quartile	30	42,422/155,714	27.4% (24.3-30.6)	99.3% (99.2-99.4)	<0.001	0.030
Second quartile	11	2,332/6,908	33.1% (25.0-41.6%)	98.1% (97.1-99.2)	<0.001	
Third quartile	27	21,046/110,472	23.7% (18.0-29.9)	99.8% (99.6-100)	<0.001	
Fourth quartile	7	3,877/7,513	41.4% (29.7-53.6)	99.0% (98.7-99.3)	<0.001	
Immediate economic vulnerability: inbound tourism expenditure (% of GDP 2016-2018)§						
Low	37	23,056/114,870	27.7% (22.0-33.8)	99.7% (99.5-99.9)	<0.001	0.760
Medium-low	18	30,509/109,908	26.9% (23.6-30.3)	99.2% (98.9-99.4)	<0.001	
Medium	9	9,266/35,685	27.6% (19.6-36.5)	99.6% (99.5-99.8)	<0.001	
Medium-high	6	3,269/11,016	29.8% (19.9-40.7)	99.3% (99.0-99.4)	<0.001	
High	5	3,477/9,128	34.1% (23.9-45.0)	99.0 (98.7-99.3)	<0.001	
Article type						
Published article	32	23,381/115,711	25.8% (20.2-31.7)	99.7% (99.5-99.9)	<0.001	0.38
Preprint reporting data	37	42,966/152,604	30.2% (26.7-33.9)	99.5% (99.2-99.8)	<0.001	
Short communication/letter to editor/correspondence	6	3,230/12,292	27.0% (20.6-33.8)	98.5% (97.9-99.3)	<0.001	

§Based on the 2020 global preparedness and vulnerability to respond to COVID-19 pandemic by the United Nations Development Programme.

¥Based on the World Economic Outlook, April 2020 by the International Monetary Fund.

£Based on the 2020 FM Global Resilience Index.

Abbreviations: CI, confidence interval; GDP, gross domestic product; NA, not applicable.

Table S8 Subgroup Analysis of Included Studies: Depression (Continued)

**Subgroup comparison**	**No. of studies**	**Cases/Total**	**Prevalence (95% CI)**	**I^2 (95% CI)**	***P* value for heterogeneity**	***P* value for difference**
Study design						
Cross-sectional study	66	46,444/200,446	27.9% (24.4-31.6)	99.7% (99.5-99.9)	<0.001	0.780
Longitudinal survey	9	23,133/80,161	28.9% (22.8-35.4)	99.6% (99.2-100)	<0.001	
Country						
Mainland China	25	18,100/103,505	28.0% (25.0-31.2)	99.7% (99.5-100)	<0.001	0.100
Non-mainland China	50	51,477/177,102	31.1% (28.1-34.2)	99.3% (99.0-99.4)	<0.001	
Sample size						
<1,000	26	3,656/12,570	27.8% (22.2-33.7)	98.1% (97.7-98.4)	<0.001	0.430
1,000-5,000	39	25,892/82,549	29.4% (24.8-34.1)	99.6% (99.5-99.8)	<0.001	
>5,000	10	40,029/185,488	23.7% (17.2-31.0)	99.8% (99.6-100)	<0.001	
% Female						
<50	15	5,755/14,923	29.9% (21.2-39.4)	99.3% (99.0-99.4)	<0.001	0.010
50-60	21	18,062/111,230	23.3% (18.5-28.4)	99.7% (99.5-99.9)	<0.001	
>60	36	44,402/147,848	30.6% (27.2-34.1)	99.4% (99.2-99.5)	<0.001	
Measurement tools						
PHQ-9	32	43,917/197,718	25.3% (21.4-29.4)	99.7% (99.5-99.9)	<0.001	<0.001
DASS-21: subscale depression	18	11,939/31,395	33.1% (26.4-40.2)	99.3% (99.1-99.4)	<0.001	
PHQ-2	6	1,799/9,474	18.8% (14.8-23.1)	96.0% (93.7-98.4)	<0.001	
CES-D	5	3,424/9,818	31.9% (11.6-56.7)	99.8% (99.6-100)	<0.001	
HADS: subscale depression	4	1,293/3,792	31.7% (25.6-38.2)	93.6% (87.3-97.2)	<0.001	
Zung-SDS	4	655/3,316	28.0 (25.0-31.2)	96.1% (93.2-98.3)	<0.001	
Others	6	6,550/25,094	37.1% (24.6-50.5)	99.7% (99.5-99.9)	<0.001	

Abbreviations: CES-D, Center for Epidemiologic Studies Depression Scale; CI, confidence interval; DASS-21, Depression, Anxiety and Stress Scale-21; HADS, Hospital Anxiety and Depression Scale; NA, not applicable; PHQ-2, Patient Health Questionnaire-2; PHQ-9, Patient Health Questionnaire-9; Zung-SDS, Zung-Self-Rating Depression Scale.

Table S9 Subgroup Analysis of Included Studies: Anxiety

Subgroup comparison	No. of studies	Cases/Total	Prevalence (95% CI)	I^2 (95% CI)	*P* value for heterogeneity	*P* value for difference
HDI groups, 2018[†]						
Low	1	249/502	49.6% (45.1-54.1)	NA	NA	<0.001
Medium	10	4,124/13,853	30.9% (16.1-48.2)	99.7% (99.5-99.9)	<0.001	
High	32	26,201/128,154	25.6% (20.1-31.6)	99.8% (99.6-100)	<0.001	
Very high	32	36,257/142,304	26.4% (23.8-29.1)	99.1% (98.6-99.4)	<0.001	
GII, 2018[†]						
<0.439	60	56,181/260,809	25.4% (22.6-28.3)	99.6% (99.5-99.9)	<0.001	0.100
≥0.439	12	10,224/22,621	34.3% (26.7-42.3)	99.2% (99.0-99.4)	<0.001	
CSI during the survey[‡]						
Less stringent (<75)	14	9,157/25,908	28.0% (19.6-37.1)	99.5% (99.1-99.7)	<0.001	0.720
Moderate stringent (75-85)	40	43,107/202,381	25.7% (22.0-29.6)	99.7% (99.5-99.9)	<0.001	
Very stringent (>85)	19	12,479/48,307	28.8% (22.2-35.8)	99.6% (99.5-99.8)	<0.001	
Hospital beds per 10,000 people, 2010-2018[§]						
Low	9	3,708/8,430	34.2% (23.4-46.0)	99.1% (98.7-99.3)	<0.001	<0.001
Medium-low	6	7,774/17,606	36.2% (24.8-48.6)	99.5% (99.1-99.7)	<0.001	
Medium	15	21,251/88,263	27.8% (23.2-32.7)	99.3% (99.0-99.4)	<0.001	
Medium-high	41	33,454/167,131	24.0% (20.4-27.9)	99.7% (99.5-99.9)	<0.001	
High	1	218/2,000	10.9% (9.6-12.4)	NA	NA	
Current health expenditure (% of GDP), 2016[§]						
Low	12	5,729/15,441	34.4% (24.8-44.8)	99.4% (99.1-99.8)	<0.001	0.240
Medium-low	29	19,740/113,482	24.1% (19.2-29.4)	99.7% (99.5-99.9)	<0.001	
Medium	4	1,181/8,437	19.8% (8.5-34.5)	99.4% (99.2-99.6)	<0.001	
Medium-high	10	12,998/44,248	24.4% (18.3-35.5)	99.7% (99.5-99.8)	<0.001	
High	18	27,006/102,324	29.4% (25.6-33.4)	99.3% (99.0-99.4)	<0.001	

[†]Based on the 2019 Human Development Report by the United Nations Development Programme.
[‡]Based on the Oxford COVID-19 Government Response Tracker—highest value during the surveys.
[§]Based on the 2020 global preparedness and vulnerability to respond to COVID-19 pandemic by the United Nations Development Programme.
Abbreviations: CI, confidence interval; CSI, Coronavirus Disease-2019 Government Response Stringency Index; GDP, gross domestic product; GII, Gender Inequality Index; HDI, Human Development Index; NA, not applicable.

Table S9 Subgroup Analysis of Included Studies: Anxiety (Continued)

Subgroup comparison	No. of studies	Cases/Total	Prevalence (95% CI)	I^2 (95% CI)	*P* value for heterogeneity	*P* value for difference
Real GDP growth 2020, estimates percent change[¥]						
Below -3.0	38	43,892/159,597	29.4% (26.2-32.6)	99.3% (99.1-99.4)	<0.001	0.110
Above -3.0	37	22,939/125,216	24.5% (19.7-29.6)	99.7% (99.5-100)	<0.001	
Resilience of a country's business environment[£]						
First quartile	28	35,244/138,379	26.3% (23.5-29.1)	99.2% (89.6-99.3)	<0.001	<0.001
Second quartile	11	2,216/7,079	33.4% (26.3-41.0)	97.6% (97.1-98.3)	<0.001	
Third quartile	28	20,422/121,088	21.9% (17.4-26.8)	99.7% (99.5-99.9)	<0.001	
Fourth quartile	8	8,949/18,267	39.5% (30.6-48.7)	99.1% (98.7-99.3)	<0.001	
Immediate economic vulnerability: inbound tourism expenditure (% of GDP 2016-2018)[§]						
Low	39	28,140/131,209	26.9% (21.3-32.8)	99.8% (99.6-100)	<0.001	0.200
Medium-low	14	23,225/89,719	30.1% (26.5-33.9)	99.0% (98.6-99.3)	<0.001	
Medium	9	9,360/40,887	23.2% (16.2-31.2)	99.6% (99.5-99.9)	<0.001	
Medium-high	8	3,878/13,870	29.3% (22.2-36.9)	98.9% (98.5-99.2)	<0.001	
High	5	2,228/9,128	22.1% (16.1-28.8)	97.8% (97.0-99.3)	<0.001	
Article type						
Published article	34	28,220/137,081	24.5% (19.2-30.2)	99.8% (99.5-99.9)	<0.001	0.110
Preprint reporting data	36	35,865/136,453	29.7% (26.4-33.2)	99.3% (99.0-99.4)	<0.001	
Short communication/letter to editor/correspondence	5	2,746/11,279	23.8% (18.1-29.9)	98.1% (97.1-99.3)	<0.001	
Study design						
Cross-sectional study	68	48,608/212,400	26.2% (22.8-29.8)	99.7% (99.5-99.9)	<0.001	0.040
Longitudinal survey	7	18,223/72,413	33.7% (27.5-40.2)	99.4% (99.1-99.7)	<0.001	
Country						
Mainland China	25	18,159/108,698	22.2% (17.2-27.7)	99.7% (99.5-99.9)	<0.001	0.030
Non-mainland China	50	48,672/176,115	29.4% (26.2-32.7)	99.5% (99.1-99.8)	<0.001	

[¥]Based on the World Economic Outlook, April 2020 by the International Monetary Fund.

[£]Based on the 2020 FM Global Resilience Index.

Abbreviations: CI, confidence interval; GDP, gross domestic product; NA, not applicable.

Table S9 Subgroup Analysis of Included Studies: Anxiety (Continued)

Subgroup comparison	No. of studies	Cases/Total	Prevalence (95% CI)	I^2 (95% CI)	*P* value for heterogeneity	*P* value for difference
Sample size						
<1,000	26	3,431/12,702	26.8% (20.9-33.1)	98.4% (98.0-99.2)	<0.001	0.610
1,000-5,000	38	23,196/81,455	28.0% (23.8-32.3)	99.5% (99.0-99.8)	<0.001	
>5,000	11	40,204/190,656	23.7% (16.9-31.3)	99.9% (99.6-100)	<0.001	
% Female						
<50	15	5,156/14,302	30.3% (21.8-39.6)	99.2% (98.8-99.4)	<0.001	0.130
50-60	18	13,523/94,997	20.8% (15.3-26.8)	99.7% (99.5-99.9)	<0.001	
>60	39	46,111/168,908	28.6% (25.5-31.8)	99.5% (99.2-99.8)	<0.001	
Measurement tools						
GAD-7	41	45,960/225,609	24.7% (21.5-27.9)	99.6% (99.5-99.8)	<0.001	<0.001
DASS-21: subscale anxiety	18	15,708/41,241	31.5% (24.8-38.6)	99.5% (99.1-99.7)	<0.001	
GAD-2	6	2,597/9,460	27.7% (16.3-40.8)	99.4% (99.0-99.8)	<0.001	
HADS: subscale anxiety	4	1,733/3,792	42.8% (26.0-60.4)	99.1% (98.8-99.4)	<0.001	
Zung-SAS	3	245/2,708	9.4% (5.8-13.6)	NA	NA	
Others	3	588/2,003	31.2% (15.3-49.8)	NA	NA	

Abbreviations: CI, confidence interval; DASS-21, Depression, Anxiety and Stress Scale-21; GAD-2, General Anxiety Disorder-2; GAD-7, General Anxiety Disorder-7; HADS, Hospital Anxiety and Depression Scale; NA, not applicable; Zung Self-Rating Anxiety Scale.

Table S10 Subgroup Analysis of Included Studies: Post-Traumatic Stress Symptoms

Subgroup comparison	No. of studies	Cases/Total	Prevalence (95% CI)	I^2 (95% CI)	*P* value for heterogeneity	*P* value for difference
HDI groups, 2018†						
Low	1	215/502	42.8% (38.4-47.3)	NA	NA	<0.001
Medium	1	119/653	18.2% (15.3-21.4)	NA	NA	
High	14	4,911/19,538	21.1% (8.8-36.8)	99.8% (99.5-100)	<0.001	
Very high	12	11,297/35,754	26.8% (19.0-35.4)	99.6% (99.5-99.9)	<0.001	
GII, 2018†						
<0.439	25	15,943/54,782	22.6% (15.3-31.0)	99.8% (99.6-100)	<0.001	<0.001
≥0.439	2	384/1,163	31.9% (29.3-34.7)	NA	NA	
CSI during the survey‡						
Less stringent (<75)	4	1,260/6,987	19.3% (13.5-25.8)	97.4% (95.1-98.3)	<0.001	0.030
Moderate stringent (75-85)	15	5,181/21,741	21.0% (9.4-35.8)	99.8% (99.6-100)	<0.001	
Very stringent (>85)	9	10,101/27,719	31.9% (25.1-39.0)	99.0% (98.6-99.2)	<0.001	
Hospital beds per 10,000 people, 2010-2018§						
Low	1	119/653	18.2% (15.3-21.4)	NA	NA	<0.001
Medium-low	1	265/510	52.0% (47.5-56.4)	NA	NA	
Medium	6	1,338/7,091	23.4% (12.5-36.4)	99.3% (99.1-99.4)	<0.001	
Medium-high	19	14,605/47,691	22.4% (13.8-32.4)	99.8% (99.5-100)	<0.001	
High	NA	NA	NA	NA	NA	
Current health expenditure (% of GDP), 2016§						
Low	2	334/1,155	28.2% (25.6-30.8)	NA	NA	<0.001
Medium-low	14	4,944/19,852	20.6% (8.6-36.1)	99.8% (99.5-100)	<0.001	
Medium	2	476/1,137	41.7% (38.9-44.6)	NA	NA	
Medium-high	7	10,056/29,409	28.6% (19.8-38.2)	99.6% (99.3-99.8)	<0.001	
High	3	732/4,894	16.5% (5.0-32.8)	NA	NA	

†Based on the 2019 Human Development Report by the United Nations Development Programme.
‡Based on the Oxford COVID-19 Government Response Tracker—highest value during the surveys.
§Based on the 2020 global preparedness and vulnerability to respond to COVID-19 pandemic by the United Nations Development Programme.
Abbreviations: CI, confidence interval; CSI, Coronavirus Disease-2019 Government Response Stringency Index; GDP, gross domestic product; GII, Gender Inequality Index; HDI, Human Development Index; NA, not applicable.

Table S10 Subgroup Analysis of Included Studies: Post-Traumatic Stress Symptoms (Continued)

Subgroup comparison	No. of studies	Cases/Total	Prevalence (95% CI)	I^2 (95% CI)	*P* value for heterogeneity	*P* value for difference
Real GDP growth 2020, estimates percent change[¥]						
Below -3.0	13	11,434/36,325	26.6% (19.3-34.7)	99.6% (99.3-99.8)	<0.001	0.570
Above -3.0	15	5,108/20,122	21.9% (9.8-37.2)	99.8% (99.5-100)	<0.001	
Resilience of a country's business environment[£]						
First quartile	10	10,788/34,303	24.7% (16.3-34.1)	99.7% (99.5-99.9)	<0.001	<0.001
Second quartile	3	628/2,104	31.0% (14.6-50.3)	NA	NA	
Third quartile	14	4,911/19,538	21.1% (8.8-36.8)	99.8% (99.6-100)	<0.001	
Fourth quartile	1	215/502	42.8% (38.4-47.3)	NA	NA	
Immediate economic vulnerability: inbound tourism expenditure (% of GDP 2016-2018)[§]						
Low	14	4,781/19,580	19.6% (7.9-34.9)	99.8% (99.6-100)	<0.001	0.390
Medium-low	4	1,009/5,428	24.2% (8.8-44.1)	99.6% (99.3-99.8)	<0.001	
Medium	4	7,523/21,803	23.4% (14.6-33.5)	99.1% (98.7-99.4)	<0.001	
Medium-high	6	3,229/9,636	36.1% (22.2-51.4)	99.5% (99.1-99.8)	<0.001	
High	NA	NA	NA	NA	NA	
Article type						
Published article	15	6,689/17,890	28.2% (14.9-43.7)	99.8% (99.5-99.9)	<0.001	0.230
Preprint reporting data	8	8,222/28,223	22.0 (11.1-35.4)	99.8% (99.6-100)	<0.001	
Short communication/letter to editor/correspondence	5	1,631/10,334	16.0% (10.8-22.0)	98.2% (97.1-99.4)	<0.001	
Study design						
Cross-sectional study	25	15,939/51,009	26.0% (18.2-34.6)	99.8% (99.5-99.9)	<0.001	0.010
Longitudinal survey	3	606/5,438	10.6% (4.7-18.5)	NA	NA	
Country						
Mainland China	12	4,447/18,425	18.0% (5.9-34.8)	99.8% (99.6-100)	<0.001	0.220
Non-mainland China	16	12,095/38,022	29.0% (22.1-36.4%)	99.5% (99.3-99.7)	<0.001	

[¥]Based on the World Economic Outlook, April 2020 by the International Monetary Fund.

[£]Based on the 2020 FM Global Resilience Index.

Abbreviations: CI, confidence interval; GDP, gross domestic product; NA, not applicable.

Table S10 Subgroup Analysis of Included Studies: Post-Traumatic Stress Symptoms (Continued)

**Subgroup comparison**	**No. of studies**	**Cases/Total**	**Prevalence (95% CI)**	I^2 **(95% CI)**	***P* value for heterogeneity**	***P* value for difference**
Sample size						
<1,000	12	1,843/6,981	23.9% (15.6-33.2)	98.7% (97.9-99.3)	<0.001	<0.001
1,000-5,000	15	8,033/31,319	23.4% (12.6-36.5)	99.8% (99.5-100)	<0.001	
>5,000	1	6,666/18,147	36.7% (36.0-37.4)	NA	NA	
% Female						
<50	5	868/3,403	26.9% (13.6-42.7)	99.0% (98.7-99.3)	<0.001	<0.001
50-60	4	2,348/5,058	26.9% (0.9-70.0)	99.8% (99.5-100)	<0.001	
>60	18	13,244/46,814	23.8% (15.9-32.8)	99.9% (99.8-100)	<0.001	
No data	1	82/1,172	7.0% (5.6-8.6)	NA	NA	
Measurement tools						
IES-R	14	5,202/16,777	28.4% (18.5-39.6)	99.6% (99.4-99.7)	<0.001	0.620
PCL-5	8	3,217/12,472	18.3% (3.3-41.4)	99.9% (99.8-100)	<0.001	
Others	6	8,123/27,198	22.3% (11.0-36.2)	99.8% (99.5-99.9)	<0.001	

Abbreviations: CI, confidence interval; IES-R, Impact of Event Scale-Revised; NA, not applicable; PCL-5, Posttraumatic Stress Disorder Checklist for Diagnostic and Statistical Manual of Mental Disorders, 5th.

Table S11 Subgroup Analysis of Included Studies: Stress

Subgroup comparison	No. of studies	Cases/Total	Prevalence (95% CI)	I^2 (95% CI)	*P* value for heterogeneity	*P* value for difference
HDI groups, 2018†						
Low	NA	NA	NA	NA	NA	0.580
Medium	5	3,176/6,427	39.7% (24.4-56.1)	99.4% (99.0-99.7)	<0.001	
High	7	18,196/64,164	40.3% (19.5-63.0)	99.9% (99.7-100)	<0.001	
Very high	10	11,072/40,258	32.3% (26.2-38.7)	99.3% (99.1-99.4)	<0.001	
GII, 2018†						
<0.439	16	29,041/104,041	34.1% (27.2-41.5)	99.8% (99.5-100)	<0.001	0.570
≥0.439	5	3,176/6,427	39.7% (24.4-56.1)	99.4% (99.1-99.8)	<0.001	
CSI during the survey‡						
Less stringent (<75)	3	1,121/2,051	58.4% (48.5-68.0)	NA	NA	<0.001
Moderate stringent (75-85)	11	22,107/77,808	30.9% (20.7-42.1)	99.8% (99.5-100)	<0.001	
Very stringent (>85)	8	9,216/30,990	36.4% (25.2-48.4)	99.7% (99.5-100)	<0.001	
Hospital beds per 10,000 people, 2010-2018§						
Low	5	3,176/6,427	39.7% (24.4-56.1)	99.4% (99.0-99.8)	<0.001	<0.001
Medium-low	NA	NA	NA	NA	NA	
Medium	2	3,153/4,364	72.3 (71.0-73.6)	NA	NA	
Medium-high	14	25,888/99,677	29.4% (24.6-34.3)	99.5% (99.1-99.9)	<0.001	
High	NA	NA	NA	NA	NA	
Current health expenditure (% of GDP), 2016§						
Low	6	6,092/10,431	45.4% (30.0-61.3)	99.6% (99.3-99.9)	<0.001	0.350
Medium-low	5	15,043/59,800	28.9% (12.6-48.6)	99.7% (99.5-99.9)	<0.001	
Medium	NA	NA	NA	NA	NA	
Medium-high	5	6,889/26,746	32.7% (23.5-42.7)	99.5% (99.1-99.8)	<0.001	
High	5	4,193/13,491	33.0% (23.3-43.6)	99.2% (98.9-99.4)	<0.001	

†Based on the 2019 Human Development Report by the United Nations Development Programme.

‡Based on the Oxford COVID-19 Government Response Tracker—highest value during the surveys.

§Based on the 2020 global preparedness and vulnerability to respond to COVID-19 pandemic by the United Nations Development Programme.

Abbreviations: CI, confidence interval; CSI, Coronavirus Disease-2019 Government Response Stringency Index; GDP, gross domestic product; GII, Gender Inequality Index; HDI, Human Development Index; NA, not applicable.

Table S11 Subgroup Analysis of Included Studies: Stress (Continued)

**Subgroup comparison**	**No. of studies**	**Cases/Total**	**Prevalence (95% CI)**	***I^2* (95% CI)**	***P* value for heterogeneity**	***P* value for difference**
Real GDP growth 2020, estimates percent change[¥]						
Below -3.0	12	14,225/44,622	38.4% (28.5-48.8)	99.8% (99.5-100)	<0.001	0.630
Above -3.0	10	18,219/66,227	34.2% (22.0-47.7)	99.7% (99.5-100)	<0.001	
Resilience of a country's business environment[£]						
First quartile	10	11,072/40,258	32.3% (26.2-38.7)	99.3% (99.0-99.4)	<0.001	0.220
Second quartile	3	589/1,733	39.3% (17.0-64.4)	NA	NA	
Third quartile	6	17,959/63,804	36.1% (14.8-60.7)	99.9% (99.7-100)	<0.001	
Fourth quartile	3	2,824/5,054	48.9% (34.6-63.3)	NA	NA	
Immediate economic vulnerability: inbound tourism expenditure (% of GDP 2016-2018)[§]						
Low	11	18,456/66,587	37.0% (24.8-50.2)	99.7% (99.5-100)	<0.001	0.600
Medium-low	NA	NA	NA	NA	NA	
Medium	6	9,045/34,074	30.1% (22.8-38.0)	99.5% (99.1-99.8)	<0.001	
Medium-high	2	1,548/4,523	34.1% (32.7-35.4)	NA	NA	
High	3	3,395/5,665	50.3% (14.6-85.7)	NA	NA	
Article type						
Published article	10	18,944/74,007	26.7% (21.6-32.1)	99.2% (98.8-99.4)	<0.001	<0.001
Preprint reporting data	11	12,704/35,670	43.0% (29.6-57.1)	99.8% (99.6-100)	<0.001	
Short communication/letter to editor/correspondence	1	796/1,172	67.9% (65.2-70.6)	NA	NA	
Study design						
Cross-sectional study	19	29,272/106,419	33.5% (28.2-39.1)	99.6% (99.5-99.9)	<0.001	<0.001
Longitudinal survey	3	3,172/4,430	57.6% (41.6-72.8)	NA	NA	
Country						
Mainland China	5	15,043/59,800	28.9% (12.6-48.6)	99.7% (99.5-99.9)	<0.001	0.360
Non-mainland China	17	17,401/51,049	38.8% (30.1-47.8)	99.7% (99.5-100)	<0.001	

[¥]Based on the World Economic Outlook, April 2020 by the International Monetary Fund.

[£]Based on the 2020 FM Global Resilience Index.

Abbreviations: CI, confidence interval; GDP, gross domestic product; NA, not applicable.

Table S11 Subgroup Analysis of Included Studies: Stress (Continued)

**Subgroup comparison**	**No. of studies**	**Cases/Total**	**Prevalence (95% CI)**	***I^2* (95% CI)**	***P* value for heterogeneity**	***P* value for difference**
Sample size						
<1,000	7	1,001/3,358	32.3% (15.6-51.6)	99.2% (98.8-99.4)	<0.001	0.090
1,000-5,000	11	10,260/22,305	42.5% (30.4-55.1)	99.7% (99.5-99.9)	<0.001	
>5,000	4	21,183/85,186	27.9% (22.9-33.2)	99.5% (99.3-99.7)	<0.001	
% Female						
<50	5	3,038/6,600	31.7% (11.9-55.9)	99.7% (99.5-99.9)	<0.001	<0.001
50-60	2	14,044/57,060	24.6% (24.2-24.9)	NA	NA	
>60	14	14,566/46,017	35.4% (26.7-44.6)	99.7% (99.5-100)	<0.001	
No data	1	796/1,172	76.9% (65.2-70.6)	NA	NA	
Measurement tools						
DASS-21: subscale stress	17	10,745/30,487	32.6% (25.9-39.8)	99.3% (99.0-99.4)	<0.001	0.010
PSS-10	4	7,882/23,683	57.0% (23.3-87.3)	99.9% (99.7-100)	<0.001	
Others	1	13,817/56,679	24.4% (24.0-24.7)	NA	NA	

Abbreviations: CI, confidence interval; DASS-21, Depression, Anxiety and Stress Scale-21; NA, not applicable; PSS-10, Perceived Stress Scale-10.

Table S12 Subgroup Analysis of Included Studies: Psychological Distress

Subgroup comparison	No. of studies	Cases/Total	Prevalence (95% CI)	I^2 (95% CI)	*P* value for heterogeneity	*P* value for difference
HDI groups, 2018†						
Low	NA	NA	NA	NA	NA	<0.001
Medium	1	350/505	69.3% (65.1-73.3)	NA	NA	
High	8	20,768/58,437	50.6% (33.6-67.6)	99.7% (99.5-100)	<0.001	
Very high	9	10,484/22,873	47.4% (35.4-59.4)	99.6% (99.5-99.9)	<0.001	
GII, 2018†						
<0.439	14	30,182/79,397	47.0% (38.4-55.6)	99.7% (99.5-99.9)	<0.001	0.420
≥0.439	4	1,420/2,418	60.8% (29.2-88.0)	99.6% (99.1-99.8)	<0.001	
CSI during the survey‡						
Less stringent (<75)	5	2,422/4,822	49.0% (32.8-65.2)	99.2% ()	<0.001	0.710
Moderate stringent (75-85)	6	24,149/68,511	45.8% (35.6-56.1)	99.7% ()	<0.001	
Very stringent (>85)	7	5,031/8,482	54.4% (36.8-71.5)	99.6% ()	<0.001	
Hospital beds per 10,000 people, 2010-2018§						
Low	1	350/505	69.3% (65.1-73.3)	NA	NA	<0.001
Medium-low	3	1,070/1,913	57.8% (16.3-93.5)	NA	NA	
Medium	5	6,647/16,343	54.2% (36.9-71.0)	99.6% (99.5-99.9)	<0.001	
Medium-high	8	23,156/62,388	41.2% (27.1-56.2)	99.8% (99.5-100)	<0.001	
High	1	379/666	56.9% (53.1-60.7)	NA	NA	
Current health expenditure (% of GDP), 2016§						
Low	1	350/505	69.3% (65.1-73.3)	NA	NA	<0.001
Medium-low	4	19,025/55,613	42.1% (22.5-63.0)	99.7% (99.5-99.9)	<0.001	
Medium	NA	NA	NA	NA	NA	
Medium-high	6	4,777/8,137	42.7% (26.0-60.4)	99.5% (99.1-99.8)	<0.001	
High	7	7,450/17,560	58.0% (42.9-72.4)	99.6% (99.2-99.9)	<0.001	

†Based on the 2019 Human Development Report by the United Nations Development Programme.
‡Based on the Oxford COVID-19 Government Response Tracker—highest value during the surveys.
§Based on the 2020 global preparedness and vulnerability to respond to COVID-19 pandemic by the United Nations Development Programme.
Abbreviations: CI, confidence interval; CSI, Coronavirus Disease-2019 Government Response Stringency Index; GII, Gender Inequality Index; NA, not applicable.

Table S12 Subgroup Analysis of Included Studies: Psychological Distress (Continued)

Subgroup comparison	No. of studies	Cases/Total	Prevalence (95% CI)	I^2 (95% CI)	*P* value for heterogeneity	*P* value for difference
Real GDP growth 2020, estimates percent change[¥]						
Below -3.0	14	12,581/26,078	54.6% (43.3-65.6)	99.6% (99.5-99.9)	<0.001	0.040
Above -3.0	4	19,021/55,737	34.5% (20.5-49.9)	99.5% (99.2-99.7)	<0.001	
Resilience of a country's business environment[£]						
First quartile	9	10,484/22,873	47.4% (35.4-59.4)	99.6% (99.5-99.8)	<0.001	<0.001
Second quartile	2	1,027/1,292	80.2% (78.0-82.3)	NA	NA	
Third quartile	4	19,025/55,613	42.1% (22.5-63.0)	99.7% (99.5-99.9)	<0.001	
Fourth quartile	3	1,066/2,037	47.3% (17.5-78.3)	NA	NA	
Immediate economic vulnerability: inbound tourism expenditure (% of GDP 2016-2018)[§]						
Low	8	20,764/58,561	46.7% (31.4-62.3)	99.7% (99.5-99.9)	<0.001	<0.001
Medium-low	2	4,858/12,740	38.1% (37.3-39.0)	NA	NA	
Medium	4	1,572/3,366	46.2% (27.6-65.3)	99.0% (98.6-99.3)	<0.001	
Medium-high	3	4,054/6,767	49.2% (23.9-74.7)	NA	NA	
High	1	354/381	92.9% (89.9-95.3)	NA	NA	
Article type						
Published article	6	5,144/10,142	37.4% (19.2-57.7)	99.8% (99.6-100)	<0.001	0.080
Preprint reporting data	8	19,375/55,730	64.6% (44.9-82.1)	99.7% (99.5-99.9)	<0.001	
Short communication/letter to editor/correspondence	4	7,083/15,943	39.8% (28.3-51.8)	99.1% (98.7-99.2)	<0.001	
Study design						
Cross-sectional study	15	26,473/67,308	54.2% (42.4-65.7)	99.8% (99.6-100)	<0.001	<0.001
Longitudinal survey	3	5,129/14,507	30.2% (22.5-38.4)	NA	NA	

[§]Based on the 2020 global preparedness and vulnerability to respond to COVID-19 pandemic by the United Nations Development Programme.

[¥]Based on the World Economic Outlook, April 2020 by the International Monetary Fund.

[£]Based on the 2020 FM Global Resilience Index.

Abbreviations: CI, confidence interval; GDP, gross domestic product; NA, not applicable.

Table S12 Subgroup Analysis of Included Studies: Psychological Distress (Continued)

Subgroup comparison	No. of studies	Cases/Total	Prevalence (95% CI)	I^2 (95% CI)	*P* value for heterogeneity	*P* value for difference
Country						
Mainland China	3	18,671/55,232	23.9% (12.6-37.4)	NA	NA	<0.001
Non-mainland China	15	12,931/26,583	55.6% (44.7-66.2)	99.6% (99.5-99.9)	<0.001	
Sample size						
<1,000	10	3,143/5,689	57.0% (38.5-74.5)	99.5% (99.2-99.8)	<0.001	0.040
1,000-5,000	6	5,825/11,322	43.4% (25.2-62.5)	99.8% (99.6-100)	<0.001	
>5,000	2	22,634/64,804	34.9% (34.6-35.3)	NA	NA	
% Female						
<50	4	1,736/4,217	44.6% (21.7-68.6)	99.6% (99.2-99.8)	<0.001	0.810
50-60	7	6,274/15,454	49.1% (32.4-66.0)	99.5% (99.2-99.7)	<0.001	
>60	7	23,592/62,144	54.1% (36.9-70.7)	99.8% (99.5-100)	<0.001	
Measurement tools						
CPDI	5	19,911/55,409	56.8% (33.3-78.7)	99.7% (99.5-99.9)	<0.001	0.390
Kessler-6	5	2,019/5,444	37.2% (21.1-54.8)	99.4% (99.1-99.7)	<0.001	
Kessler-10	3	923/1,547	65.2% (31.9-91.7)	NA	NA	
Others	5	8,749/19,415	47.2% (27.6-67.2)	99.8% (99.6-100)	<0.001	

Abbreviations: CI, confidence interval; CPDI, COVID-19 Peri-traumatic Distress Index, NA, not applicable.

Table S13 Subgroup Analysis of Included Studies: Sleep Problems

Subgroup comparison	No. of studies	Cases/Total	Prevalence (95% CI)	I^2 (95% CI)	*P* value for heterogeneity	*P* value for difference
HDI groups, 2018[†]						
Low	1	76/502	15.1% (12.1-18.6)	NA	NA	<0.001
Medium	NA	NA	NA	NA	NA	
High	10	22,421/76,543	27.1% (20.2-34.5)	99.6% (99.5-99.8)	<0.001	
Very high	4	3,140/22,489	32.2% (9.1-61.3)	99.9% (99.7-100)	<0.001	
GII, 2018[†]						
<0.439	14	25,561/99,032	28.6% (20.4-37.5)	99.8% (99.6-100)	<0.001	NA
≥0.439	NA	NA	NA	NA	NA	
CSI during the survey[‡]						
Less stringent (<75)	2	946/1,979	47.8% (45.6-50.0)	NA	NA	<0.001
Moderate stringent (75-85)	12	23,385/79,408	26.8% (20.7-33.4)	99.5% (99.1-99.8)	<0.001	
Very stringent (>85)	1	1,306/18,147	7.2% (6.8-7.6)	NA	NA	
Hospital beds per 10,000 people, 2010-2018[§]						
Low	NA	NA	NA	NA	NA	<0.001
Medium-low	NA	NA	NA	NA	NA	
Medium	2	2,417/4,673	51.7% (50.3-53.2)	NA	NA	
Medium-high	12	23,144/94,359	25.8% (17.8-34.7)	99.8% (99.6-100)	<0.001	
High	NA	NA	NA	NA	NA	
Current health expenditure (% of GDP), 2016[§]						
Low	3	2,493/5,175	35.0% (14.7-58.6)	NA	NA	0.610
Medium-low	9	20,264/72,539	24.2% (18.6-30.2)	99.3% (99.1-99.4)	<0.001	
Medium	NA	NA	NA	NA	NA	
Medium-high	3	2,880/21,820	30.0% (5.2-64.3)	NA	NA	
High	NA	NA	NA	NA	NA	

[†]Based on the 2019 Human Development Report by the United Nations Development Programme.
[‡]Based on the Oxford COVID-19 Government Response Tracker—highest value during the surveys.
[§]Based on the 2020 global preparedness and vulnerability to respond to COVID-19 pandemic by the United Nations Development Programme.
Abbreviations: CI, confidence interval; CSI, Coronavirus Disease-2019 Government Response Stringency Index; GDP, gross domestic product; GII, Gender Inequality Index; HDI, Human Development Index; NA, not applicable.

Table S13 Subgroup Analysis of Included Studies: Sleep Problems (Continued)

**Subgroup comparison**	**No. of studies**	**Cases/Total**	**Prevalence (95% CI)**	***I*² (95% CI)**	***P* value for heterogeneity**	***P* value for difference**
Real GDP growth 2020, estimates percent change[¥]						
Below -3.0	5	5,113/26,326	31.2% (9.0-59.5)	99.9% (99.7-100)	<0.001	0.680
Above -3.0	10	20,524/73,208	25.6% (20.2-31.3)	99.2% (98.8-99.4)	<0.001	
Resilience of a country's business environment[£]						
First quartile	2	1,992/19,457	9.2% (8.8-9.6)	NA	NA	<0.001
Second quartile	2	1,148/3,032	37.9% (36.1-39.6)	NA	NA	
Third quartile	10	22,421/76,543	27.1% (20.2-34.5)	99.6% (99.5-99.9)	<0.001	
Fourth quartile	1	76/502	15.1% (12.1-18.6)	NA	NA	
Immediate economic vulnerability: inbound tourism expenditure (% of GDP 2016-2018)[§]						
Low	10	20,340/73,041	23.2% (18.0-28.8)	99.2% (98.7-99.4)	<0.001	<0.001
Medium-low	NA	NA	NA	NA	NA	
Medium	2	1,992/19,457	9.2% (8.8-9.6)	NA	NA	
Medium-high	1	260/669	38.9% (35.2-42.7)	NA	NA	
High	2	3,045/6,367	47.8% (46.6-49.0)	NA	NA	
Article type						
Published article	8	19,640/69,040	29.1% (22.4-36.3)	99.3% (99.0-99.4)	<0.001	0.880
Preprint reporting data	4	3,799/23,322	26.6% (3.7-60.3)	99.9% (99.7-100)	<0.001	
Short communication/letter to editor/correspondence	3	2,198/7,172	24.4% (9.9-42.9)	NA	NA	
Study design						
Cross-sectional study	13	23,452/95,464	24.8% (17.3-33.2)	99.8% (99.5-100)	<0.001	<0.001
Longitudinal survey	2	2,185/4,070	53.7% (19.8-36.1)	NA	NA	
Country						
Mainland China	9	20,264/72,539	24.2% (18.6-30.2)	99.3% (99.0-99.4)	<0.001	0.500
Non-mainland China	6	5,373/26,995	32.5% (11.7-57.8)	99.9% (99.7-100)	<0.001	

[¥]Based on the World Economic Outlook, April 2020 by the International Monetary Fund.

[£]Based on the 2020 FM Global Resilience Index.

Abbreviations: CI, confidence interval; GDP, gross domestic product; NA, not applicable.

Table S13 Subgroup Analysis of Included Studies: Sleep Problems (Continued)

Subgroup comparison	No. of studies	Cases/Total	Prevalence (95% CI)	I^2 (95% CI)	*P* value for heterogeneity	*P* value for difference
Sample size						
<1,000	4	462/1,910	26.3% (13.3-41.8)	97.9% (96.0-99.1)	<0.001	0.280
1,000-5,000	9	7,305/22,798	30.8% (20.7-42.0)	99.7% (99.5-100)	<0.001	
>5,000	2	17,870/74,826	22.9% (22.6-23.2)	NA	NA	
% Female						
<50	2	174/1,175	14.8% (12.8-16.9)	NA	NA	<0.001
50-60	4	17,709/61,044	29.4% (24.0-35.1)	97.6% (96.1-99.4)	<0.001	
>60	7	6,884/31,157	36.4% (17.7-57.6)	99.9% (99.7-100)	<0.001	
No data	2	870/6,158	13.9% (13.1-14.8)	NA	NA	
Measurement tools						
ISI	8	21,894/86,069	22.3% (12.0-34.8)	99.9% (99.7-100)	<0.001	0.040
PSQI	5	2,595/10,433	32.4% (19.2-47.3)	99.5% (99.1-99.8)	<0.001	
Others	2	1,148/3,032	37.9% (36.1-39.6)	NA	NA	

Abbreviations: CI, confidence interval; ISI, Insomnia Severity Index; NA, not applicable; PSQI, Pittsburgh Sleep Quality Index.

Table S14 Sensitivity Analysis by Restricting the Analysis to Studies with a Low Risk of Bias

Primary outcomes	No. of studies	Cases/Total	Prevalence (95% CI)	I^2 (95% CI)	*P* value for heterogeneity
Depression	55	63,084/255,913	28.6% (24.1-32.3)	99.7% (99.5-99.9)	<0.001
Anxiety	57	61,857/264,968	27.4% (24.1-30.8)	99.7% (99.5-100)	<0.001
Post-traumatic stress symptoms	16	13,867/42,847	30.2% (20.3-41.1)	99.8% (99.5-100)	<0.001
Stress	17	31,079/106,575	40.1% (32.5-47.9)	99.8% (99.6-100)	<0.001
Psychological distress	8	24,165/63,776	45.4% (32.0-59.2)	99.6% (99.5-99.8)	<0.001
Sleep problems	13	25,511/98,795	27.7% (19.4-36.9)	99.8% (99.5-100)	<0.001

Abbreviation: CI, confidence interval.

Table S15 Univariable Meta-Regression of Included Studies: Depression

Covariate	No. of studies	Regression Equation β Coefficient (95% CI)*	*P* value
WHO Region (reference, Africa Region)	75	**-0.022 (-0.043 to -0.001)**	**0.036**
World Bank, by income groups (reference, lower middle income)	75	-0.041 (-0.088 to 0.007)	0.096
HDI groups, 2018† (per index unit)	75	-0.221 (-0.528 to 0.086)	0.155
GII, 2018† (per index unit)	72	**0.414 (0.196 to 0.632)**	**<0.001**
CSI during the survey‡ (per index unit)	74	0.003 (-0.001 to 0.006)	0.105
Hospital beds per 10,000 people, 2010-2018§ (per index unit)	71	**-0.003 (-0.005 to -0.001)**	**0.003**
Current health expenditure (% of GDP), 2016§ (per index unit)	73	-0.001 (-0.009 to 0.008)	0.915
Real GDP growth 2020, estimates percent change¥ (per index unit)	75	-0.001 (-0.009 to 0.007)	0.806
Resilience of a country's business environment£ (per index unit)	75	-0.001 (-0.002 to 0.001)	0.332
Immediate economic vulnerability: inbound tourism expenditure (% of GDP 2016-2018)§ (per index unit)	75	0.005 (-0.005 to 0.016)	0.341
Article type (reference, published article)	75	0.020 (-0.013 to 0.054)	0.235
Study design (cross-sectional vs longitudinal survey)	75	0.001 (-0.094 to 0.110)	0.870
Country (mainland China vs. non-mainland China)	75	**0.083 (0.016 to 0.150)**	**0.015**
Risk of bias (per 1 point)	75	-0.013 (-0.039 to 0.013)	0.329
Sample size (per number of participants)	75	-1.96e-06 (-5.46e-06 to 1.54e-06)	0.269
Age (mean, per 1 year)	51	-0.003 (-0.007 to 0.001)	0.109
Female (per %)	72	0.001 (-0.002 to 0.003)	0.622
Measurement tools (reference, PHQ-9)	75	0.009 (-0.008 to 0.026)	0.284

*β coefficient for natural logarithm of effect size for each variable of interest reflecting unit change.

†Based on the 2019 Human Development Report by the United Nations Development Programme.

‡Based on the Oxford COVID-19 Government Response Tracker—highest value during the surveys.

§Based on the 2020 global preparedness and vulnerability to respond to COVID-19 pandemic by the United Nations Development Programme.

¥Based on the World Economic Outlook, April 2020 by the International Monetary Fund.

£Based on the 2020 FM Global Resilience Index.

Abbreviations: CI, confidence interval; CSI, Coronavirus Disease-2019 Government Response Stringency Index; GDP, gross domestic product; GII, Gender Inequality Index; HDI, Human Development Index; NA, not applicable; PHQ-9, Patient Health Questionnaire-9.

Table S15 Univariable Meta-Regression of Included Studies: Anxiety (Continued)

Covariate	No. of studies	Regression Equation β Coefficient (95% CI)*	*P* value
WHO Region (reference, Africa Region)	75	**-0.038 (-0.060 to -0.015)**	**0.001**
World Bank, by income groups (reference, lower middle income)	75	-0.029 (-0.077 to 0.019)	0.236
HDI groups, 2018† (per index unit)	75	-0.208 (-0.530 to 0.114)	0.202
GII, 2018† (per index unit)	72	**0.279 (0.054 to 0.504)**	**0.016**
CSI during the survey‡ (per index unit)	73	0.001 (-0.002 to 0.004)	0.627
Hospital beds per 10,000 people, 2010-2018§ (per index unit)	72	**-0.003 (-0.004 to -0.001)**	**0.007**
Current health expenditure (% of GDP), 2016§ (per index unit)	73	0.002 (-0.008 to 0.012)	0.750
Real GDP growth 2020, estimates percent change¥ (per index unit)	75	-0.003 (-0.011 to 0.006)	0.496
Resilience of a country's business environment£ (per index unit)	75	-0.001 (-0.002 to 0.001)	0.339
Immediate economic vulnerability: inbound tourism expenditure (% of GDP 2016-2018)§ (per index unit)	75	-0.004 (-0.015 to 0.007)	0.448
Article type (reference, published article)	75	0.022 (-0.012 to 0.057)	0.204
Study design (cross-sectional vs longitudinal survey)	75	0.067 (-0.050 to 0.184)	0.259
Country (mainland China vs. non-mainland China)	75	0.064 (-0.006 to 0.134)	0.072
Risk of bias (per 1 point)	75	0.001 (-0.027 to 0.030)	0.931
Sample size (per number of participants)	75	-2.13e-06 (-5.76e-06 to 1.49e-06)	0.245
Age (mean, per 1 year)	50	-0.002 (-0.007 to 0.002)	0.307
Female (per %)	72	0.001 (-0.002 to 0.003)	0.583
Measurement tools (reference, GAD-7)	75	-0.008 (-0.017 to 0.032)	0.546

*β coefficient for natural logarithm of effect size for each variable of interest reflecting unit change.

†Based on the 2019 Human Development Report by the United Nations Development Programme.

‡Based on the Oxford COVID-19 Government Response Tracker—highest value during the surveys.

§Based on the 2020 global preparedness and vulnerability to respond to COVID-19 pandemic by the United Nations Development Programme.

¥Based on the World Economic Outlook, April 2020 by the International Monetary Fund.

£Based on the 2020 FM Global Resilience Index.

Abbreviations: CI, confidence interval; CSI, Coronavirus Disease-2019 Government Response Stringency Index; GAD-7, General Anxiety Disorder-7; GDP, gross domestic product; GII, Gender Inequality Index; HDI, Human Development Index; NA, not applicable.

Table S15 Univariable Meta-Regression of Included Studies: Post-Traumatic Stress Symptoms (Continued)

**Covariate**	**No. of studies**	**Regression Equation β Coefficient (95% CI)***	***P* value**
WHO Region (reference, Africa Region)	28	-0.024 (-0.086 to 0.038)	0.442
World Bank, by income groups (reference, lower middle income)	28	-0.014 (-0.134 to 0.108)	0.820
HDI groups, 2018† (per index unit)	28	-0.141 (-1.035 to 0.753)	0.748
GII, 2018† (per index unit)	27	0.212 (-0.603 to 1.027)	0.597
CSI during the survey‡ (per index unit)	28	0.005 (-0.006 to 0.016)	0.363
Hospital beds per 10,000 people, 2010-2018§ (per index unit)	27	-0.004 (-0.013 to 0.005)	0.387
Current health expenditure (% of GDP), 2016§ (per index unit)	28	0.001 (-0.028 to 0.031)	0.926
Real GDP growth 2020, estimates percent change¥ (per index unit)	28	-0.004 (-0.024 to 0.016)	0.689
Resilience of a country's business environment£ (per index unit)	28	-0.001 (-0.005 to 0.004)	0.829
Immediate economic vulnerability: inbound tourism expenditure (% of GDP 2016-2018)§ (per index unit)	28	0.026 (-0.014 to 0.065)	0.194
Article type (reference, published article)	28	-0.040 (-0.134 to 0.054)	0.385
Study design (cross-sectional vs longitudinal survey)	28	-0.171 (-0.430 to 0.087)	0.184
Country (mainland China vs. non-mainland China)	28	0.088 (-0.076 to 0.253)	0.280
Risk of bias (per 1 point)	28	**-0.072 (-0.137 to -0.006)**	**0.033**
Sample size (per number of participants)	28	5.66e-06 (-0.000 to 0.000)	0.650
Age (mean, per 1 year)	18	0.000 (-0.008 to 0.008)	0.993
Female (per %)	27	0.001 (-0.005 to 0.007)	0.813
Measurement tools (reference, IES-R)	28	-0.038 (-0.140 to 0.066)	0.461

*β coefficient for natural logarithm of effect size for each variable of interest reflecting unit change.
†Based on the 2019 Human Development Report by the United Nations Development Programme.
‡Based on the Oxford COVID-19 Government Response Tracker—highest value during the surveys.
§Based on the 2020 global preparedness and vulnerability to respond to COVID-19 pandemic by the United Nations Development Programme.
¥Based on the World Economic Outlook, April 2020 by the International Monetary Fund.
£Based on the 2020 FM Global Resilience Index.
Abbreviations: CI, confidence interval; CSI, Coronavirus Disease-2019 Government Response Stringency Index; GDP, gross domestic product; GII, Gender Inequality Index; HDI, Human Development Index; IES-R, Impact of Event Scale-Revised; NA, not applicable.

Table S15 Univariable Meta-Regression of Included Studies: Stress (Continued)

Covariate	No. of studies	Regression Equation β Coefficient (95% CI)*	*P* value
WHO Region (reference, Africa Region)	22	-0.043 (-0.104 to 0.017)	0.152
World Bank, by income groups (reference, lower middle income)	22	-0.045 (-0.152 to 0.063)	0.396
HDI groups, 2018† (per index unit)	22	-0.296 (-1.055 to 0.464)	0.426
GII, 2018† (per index unit)	21	0.382 (-0.070 to 0.834)	0.093
CSI during the survey‡ (per index unit)	22	-0.004 (-0.014 to 0.006)	0.458
Hospital beds per 10,000 people, 2010-2018§ (per index unit)	21	-0.004 (-0.011 to 0.002)	0.172
Current health expenditure (% of GDP), 2016§ (per index unit)	21	-0.010 (-0.041 to 0.020)	0.482
Real GDP growth 2020, estimates percent change¥ (per index unit)	22	0.001 (-0.018 to 0.020)	0.922
Resilience of a country's business environment£ (per index unit)	22	-0.002 (-0.006 to 0.002)	0.226
Immediate economic vulnerability: inbound tourism expenditure (% of GDP 2016-2018)§ (per index unit)	22	0.011 (-0.012 to 0.033)	0.336
Article type (reference, published article)	22	0.077 (-0.005 to 0.159)	0.065
Study design (cross-sectional vs longitudinal survey)	22	**0.244 (0.003 to 0.485)**	**0.047**
Country (mainland China vs. non-mainland China)	22	0.078 (-0.131 to 0.286)	0.448
Risk of bias (per 1 point)	22	-0.022 (-0.093 to 0.050)	0.539
Sample size (per number of participants)	22	2.99e-06 (-0.00 to 4.13e-06)	0.391
Age (mean, per 1 year)	18	-0.010 (-0.022 to 0.003)	0.118
Female (per %)	21	-0.001 (-0.006 to 0.004)	0.830
Measurement tools (reference, DASS-21)	22	0.078 (-0.079 to 0.235)	0.315

*β coefficient for natural logarithm of effect size for each variable of interest reflecting unit change.
†Based on the 2019 Human Development Report by the United Nations Development Programme.
‡Based on the Oxford COVID-19 Government Response Tracker—highest value during the surveys.
§Based on the 2020 global preparedness and vulnerability to respond to COVID-19 pandemic by the United Nations Development Programme.
¥Based on the World Economic Outlook, April 2020 by the International Monetary Fund.
£Based on the 2020 FM Global Resilience Index.
Abbreviations: CI, confidence interval; CSI, Coronavirus Disease-2019 Government Response Stringency Index; DASS-21, Depression, Anxiety and Stress Scale-21; GDP, gross domestic product; GII, Gender Inequality Index; HDI, Human Development Index; NA, not applicable.

Table S15 Univariable Meta-Regression of Included Studies: Psychological Distress (Continued)

Covariate	No. of studies	Regression Equation β Coefficient (95% CI)*	*P* value
WHO Region (reference, Africa Region)	**18**	**-0.093 (-0.181 to -0.004)**	**0.041**
World Bank, by income groups (reference, lower middle income)	18	-0.063 (-0.273 to 0.147)	0.533
HDI groups, 2018† (per index unit)	18	-0.480 (-1.849 to 0.889)	0.468
GII, 2018† (per index unit)	18	0.496 (-0.187 to 1.179)	0.143
CSI during the survey‡ (per index unit)	18	0.002 (-0.008 to 0.011)	0.676
Hospital beds per 10,000 people, 2010-2018§ (per index unit)	18	-0.003 (-0.011 to 0.004)	0.358
Current health expenditure (% of GDP), 2016§ (per index unit)	18	0.018 (-0.030 to 0.066)	0.439
Real GDP growth 2020, estimates percent change¥ (per index unit)	18	-0.007 (-0.042 to 0.027)	0.654
Resilience of a country's business environment£ (per index unit)	18	-0.000 (-0.005 to 0.005)	0.968
Immediate economic vulnerability: inbound tourism expenditure (% of GDP 2016-2018)§ (per index unit)	18	0.027 (-0.008 to 0.061)	0.119
Article type (reference, published article)	18	0.127 (-0.001 to 0.256)	0.052
Study design (cross-sectional vs longitudinal survey)	18	-0.234 (-0.550 to 0.081)	0.135
Country (mainland China vs. non-mainland China)	18	**0.303 (0.004 to 0.601)**	**0.047**
Risk of bias (per 1 point)	18	-0.002 (-0.112 to 0.108)	0.963
Sample size (per number of participants)	18	-3.52e-06 (-0.000 to 6.81e-06)	0.481
Age (mean, per 1 year)	9	-0.008 (-0.024 to 0.010)	0.332
Female (per %)	18	0.006 (-0.006 to 0.017)	0.329
Measurement tools (reference, CPDI)	18	-0.044 (-0.151 to 0.062)	0.387

*β coefficient for natural logarithm of effect size for each variable of interest reflecting unit change.

†Based on the 2019 Human Development Report by the United Nations Development Programme.

‡Based on the Oxford COVID-19 Government Response Tracker—highest value during the surveys.

§Based on the 2020 global preparedness and vulnerability to respond to COVID-19 pandemic by the United Nations Development Programme.

¥Based on the World Economic Outlook, April 2020 by the International Monetary Fund.

£Based on the 2020 FM Global Resilience Index.

Abbreviations: CI, confidence interval; CPDI, COVID-19 Peri-traumatic Distress Index; CSI, Coronavirus Disease-2019 Government Response Stringency Index; GDP, gross domestic product; GII, Gender Inequality Index; HDI, Human Development Index; NA, not applicable.

Table S15 Univariable Meta-Regression of Included Studies: Sleep Problems (Continued)

Covariate	No. of studies	Regression Equation β Coefficient (95% CI)*	*P* value
WHO Region (reference, Africa Region)	15	-0.010 (-0.070 to 0.049)	0.710
World Bank, by income groups (reference, lower middle income)	15	0.063 (-0.109 to 0.235)	0.442
HDI groups, 2018† (per index unit)	15	0.515 (-0.559 to 1.590)	0.319
GII, 2018† (per index unit)	14	0.773 (-0.357 to 1.903)	0.162
CSI during the survey‡ (per index unit)	15	**-0.013 (-0.026 to -0.001)**	**0.040**
Hospital beds per 10,000 people, 2010-2018§ (per index unit)	14	-0.008 (-0.019 to 0.002)	0.104
Current health expenditure (% of GDP), 2016§ (per index unit)	15	0.002 (-0.048 to 0.053)	0.924
Real GDP growth 2020, estimates percent change¥ (per index unit)	15	-0.010 (-0.029 to 0.009)	0.267
Resilience of a country's business environment£ (per index unit)	15	0.002 (-0.005 to 0.010)	0.476
Immediate economic vulnerability: inbound tourism expenditure (% of GDP 2016-2018)§ (per index unit)	15	**0.021 (0.002 to 0.039)**	**0.029**
Article type (reference, published article)	15	-0.006 (-0.109 to 0.097)	0.900
Study design (cross-sectional vs longitudinal survey)	15	0.237 (-0.008 to 0.482)	0.056
Country (mainland China vs. non-mainland China)	15	0.094 (-0.076 to 0.263)	0.254
Risk of bias (per 1 point)	15	-0.010 (-0.096 to 0.076)	0.803
Sample size (per number of participants)	15	-1.01e-06 (-7.15e-06 to 5.13e-06)	0.728
Age (mean, per 1 year)	10	-0.021 (-0.041 to 0.000)	0.051
Female (per %)	13	0.003 (-0.003 to 0.010)	0.279
Measurement tools (reference, ISI)	15	0.074 (-0.041 to 0.190)	0.189

*β coefficient for natural logarithm of effect size for each variable of interest reflecting unit change.
†Based on the 2019 Human Development Report by the United Nations Development Programme.
‡Based on the Oxford COVID-19 Government Response Tracker—highest value during the surveys.
§Based on the 2020 global preparedness and vulnerability to respond to COVID-19 pandemic by the United Nations Development Programme.
¥Based on the World Economic Outlook, April 2020 by the International Monetary Fund.
£Based on the 2020 FM Global Resilience Index.
Abbreviations: CI, confidence interval; CSI, Coronavirus Disease-2019 Government Response Stringency Index; GDP, gross domestic product; GII, Gender Inequality Index; HDI, Human Development Index; ISI, Insomnia Severity Index; NA, not applicable.

Table S16 Meta-Analysis of Included Studies with Calibration for Publication Bias[†]

**Outcomes**	**No. of studies**	***P* value for Begg's test**	***P* value for Egger's test**	**Prevalence estimates (95% CI)**	**I^2 (95% CI)**	***P* value for heterogeneity**
Depression	75	0.862	0.252	Not detected	NA	NA
Anxiety	75	0.405	0.515	Not detected	NA	NA
Post-traumatic stress symptoms	28	0.921	0.785	Not detected	NA	NA
Stress	22	0.652	0.914	Not detected	NA	NA
Psychological distress	18	0.544	0.783	Not detected	NA	NA
Sleep problems	15	0.488	0.296	Not detected	NA	NA
Suicide ideation	4	1.000	0.401	Not detected	NA	NA
Loneliness	3	0.296	0.414	Not detected	NA	NA
Somatic symptoms	3	1.000	0.399	Not detected	NA	NA
Low wellbeing	3	1.000	0.589	Not detected	NA	NA
Alcohol drinking problems	2	NA	NA	Not detected	NA	NA
Obsessive compulsive symptoms	2	NA	NA	Not detected	NA	NA
Panic disorder	1	NA	NA	NA	NA	NA
Phobia anxiety	1	NA	NA	NA	NA	NA
Adjustment disorder	1	NA	NA	NA	NA	NA
Suicide attempts	1	NA	NA	NA	NA	NA

[†]Calibration for publication was carried out, if indicated in trim and fill analysis.
Abbreviations: CI, confidence interval; NA, not applicable.

Figure S1 Study Selection

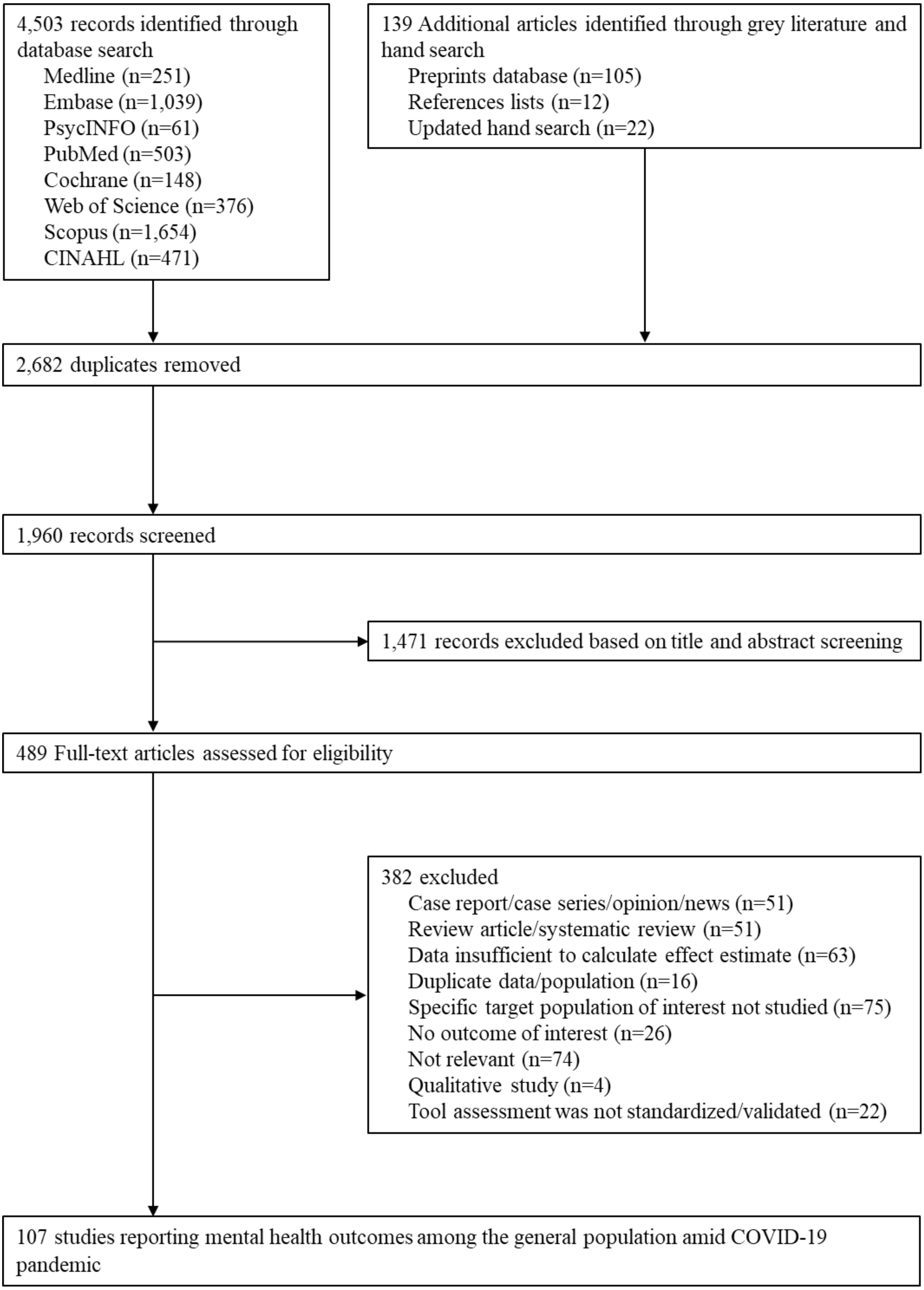

Abbreviation: COVID-19, coronavirus disease-2019.

Figure S2 Funnel Plot of Included Studies in the Meta-Analysis

(A) Depression

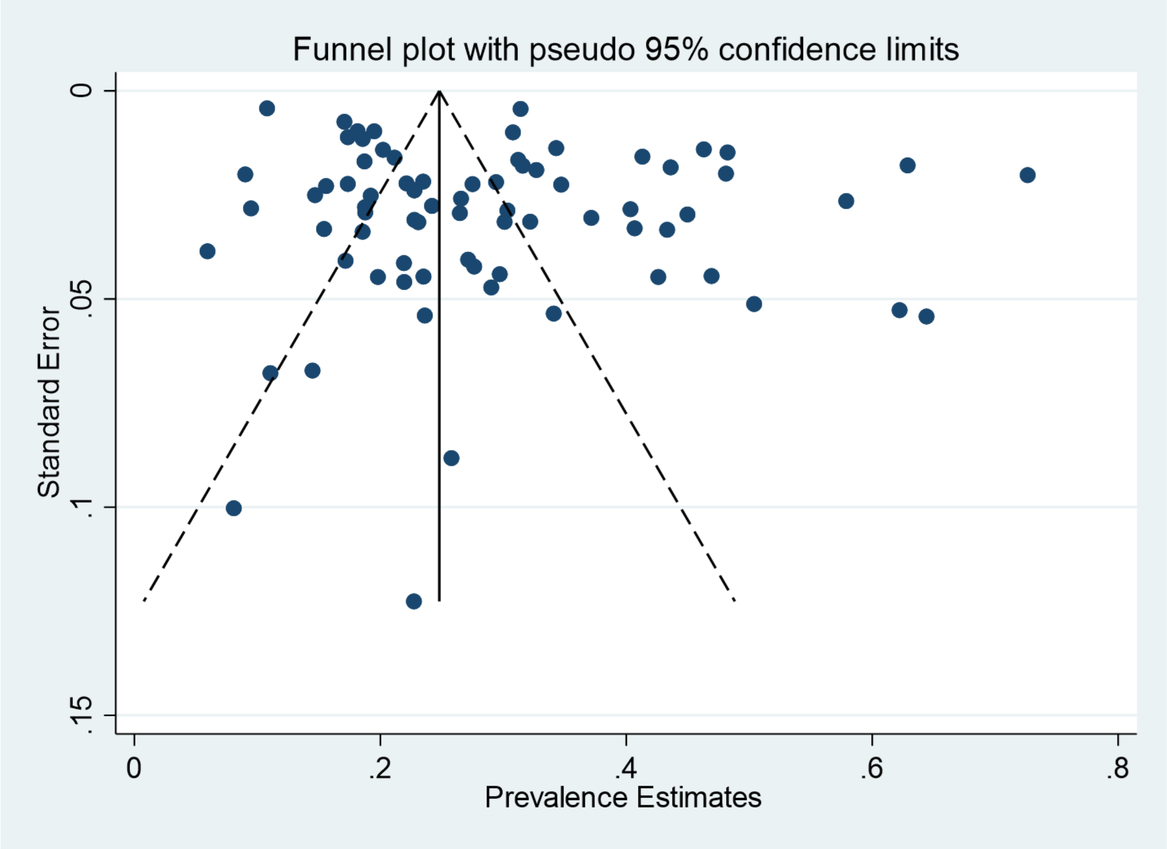

(B) Anxiety

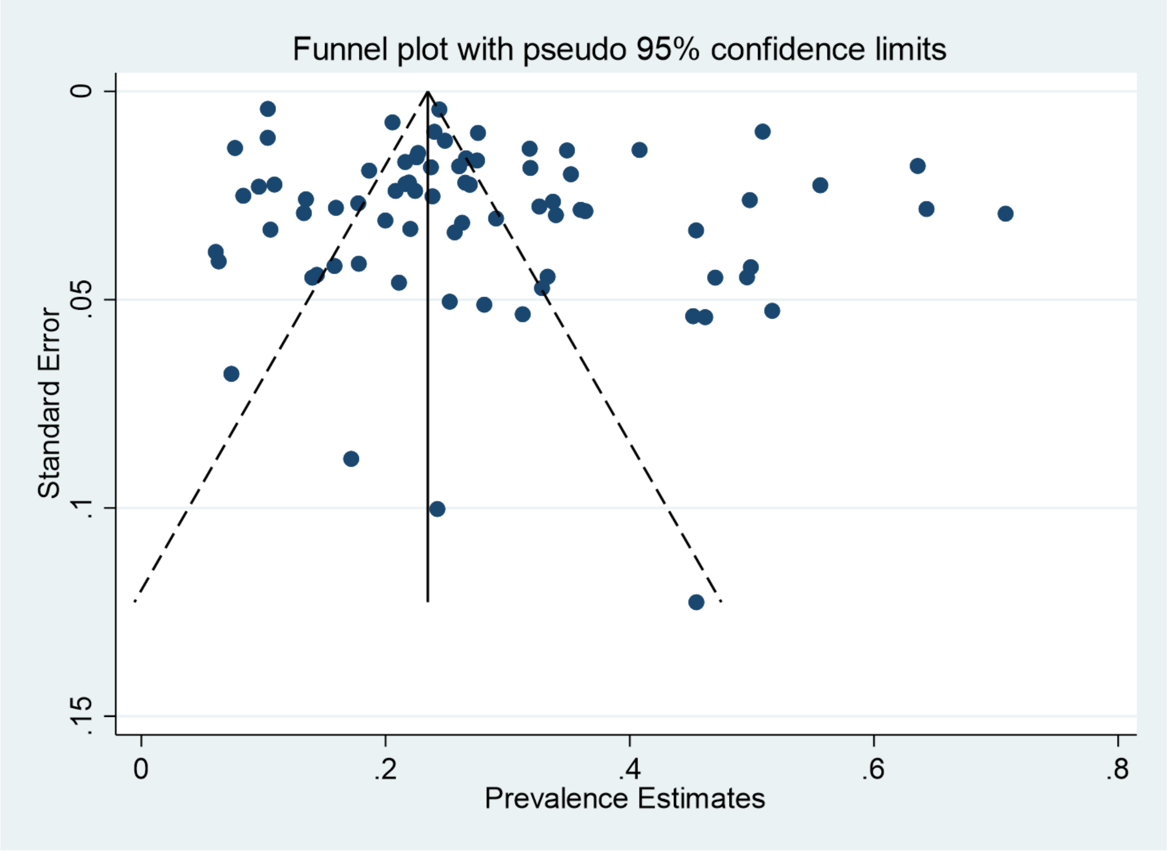

Figure S2 Funnel Plot of Included Studies in the Meta-Analysis (Continued)

(C) Post-traumatic stress symptoms

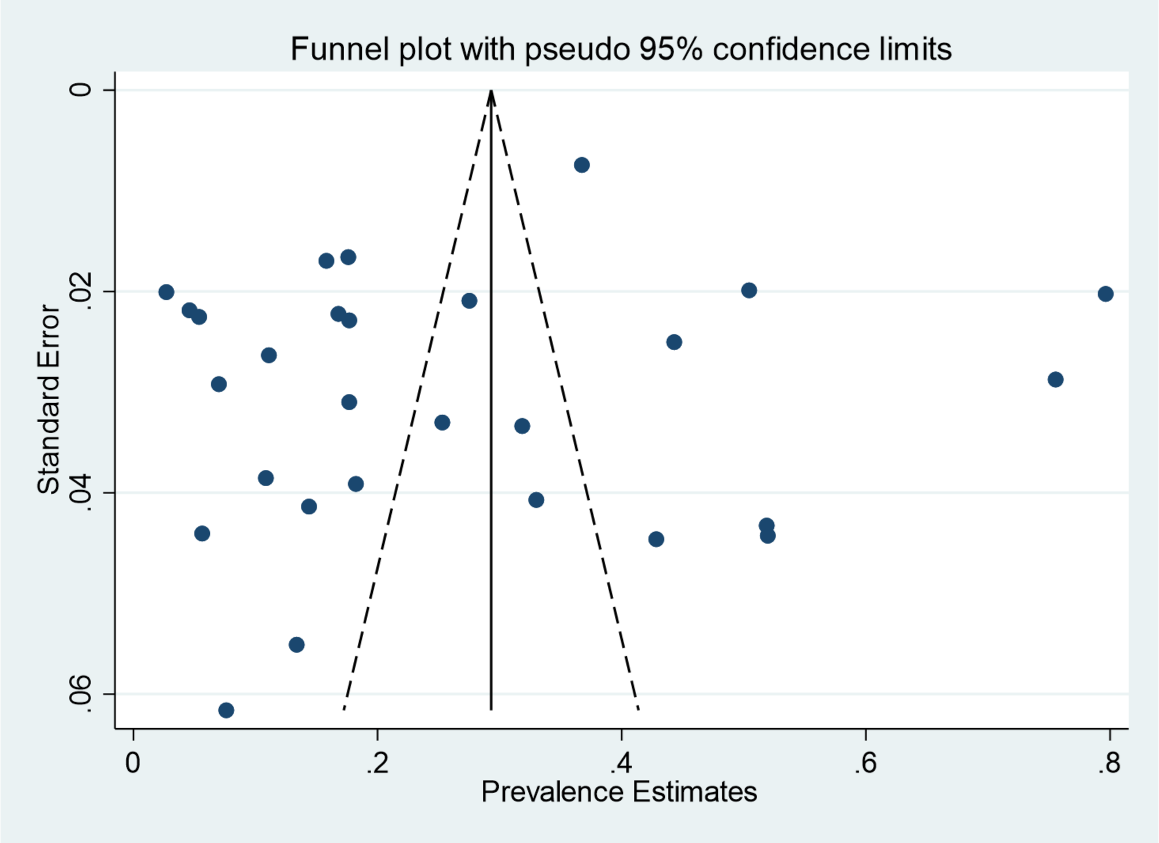

(D) Stress

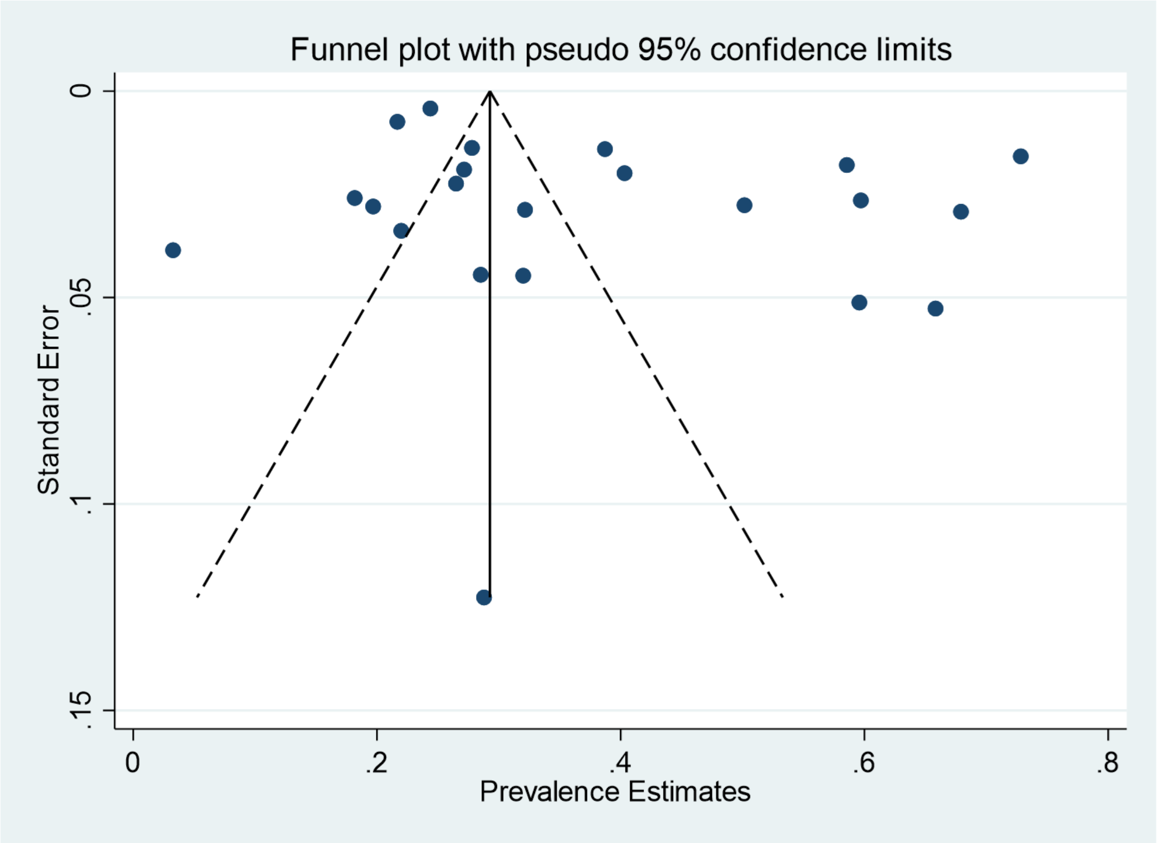

Figure S2 Funnel Plot of Included Studies in the Meta-Analysis (Continued)

(E) Psychological distress

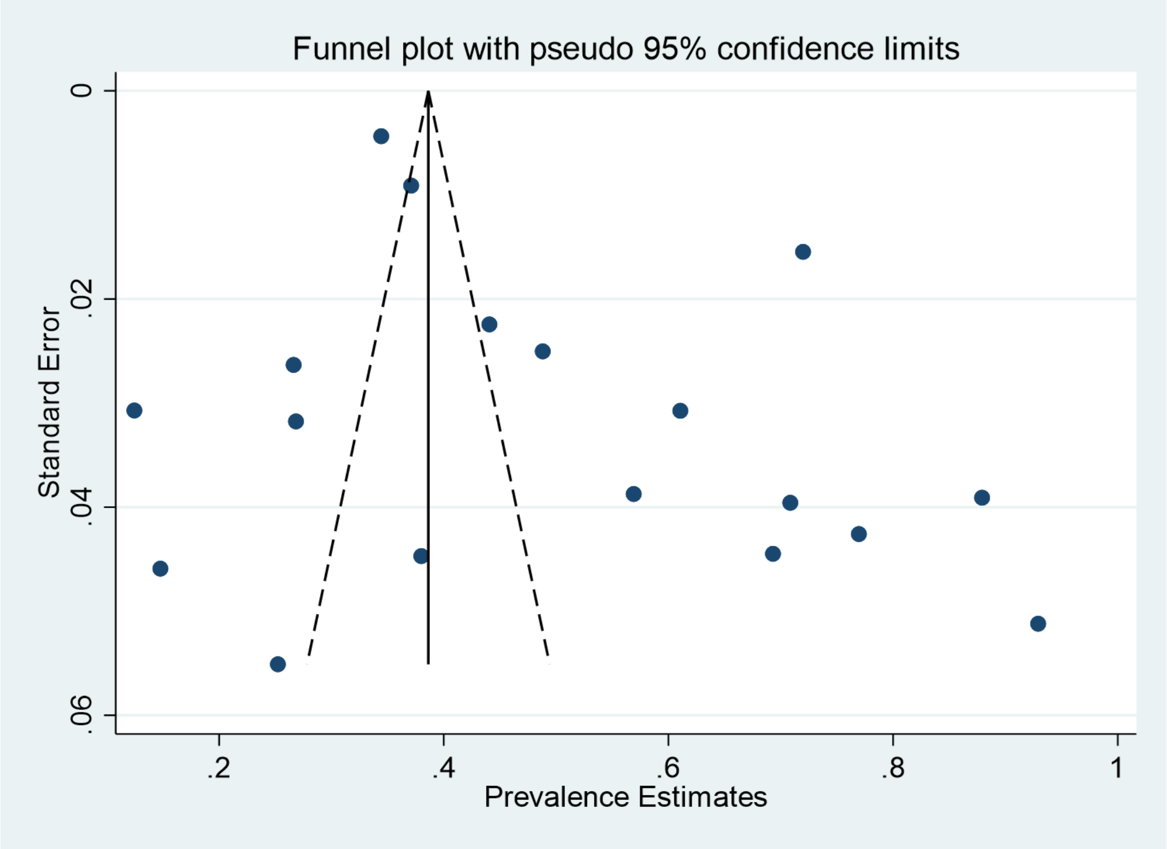

(F) Sleep problems

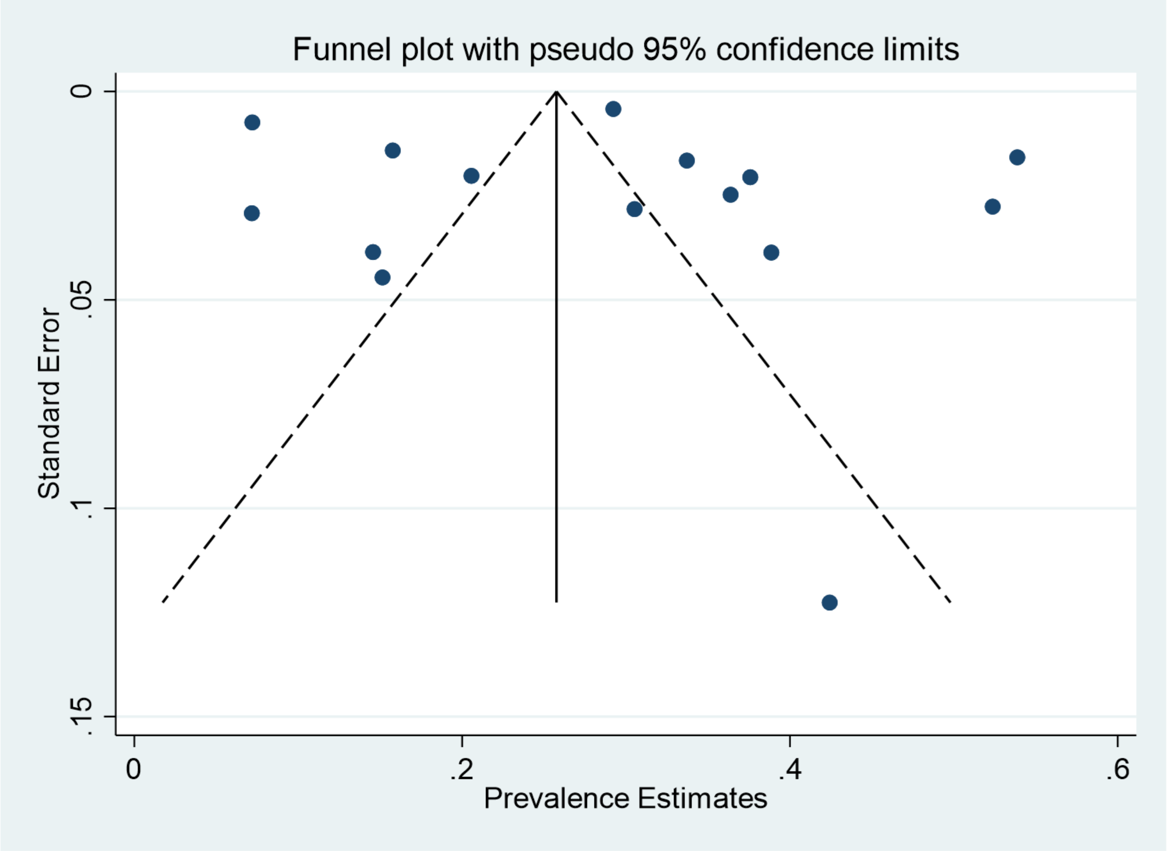

Supplement: Supplementary file 1 — Supplementary Figures and Tables. [file 41598_2021_89700_MOESM1_ESM.pdf]
